# Large-scale transcript variants dictate neoepitopes for cancer immunotherapy

**DOI:** 10.1126/sciadv.ado5600

**Published:** 2025-01-31

**Authors:** Shiliang Ji, Feifan Wang, Yongjie Wu, Haoran Hu, Zhen Xing, Jie Zhu, Shi Xu, Tiyun Han, Guilai Liu, Zengding Wu, Caiyi Fei, Lingming Kong, Jiangning Chen, Zhi Ding, Zhen Huang, Junfeng Zhang

**Affiliations:** ^1^ State Key Laboratory of Pharmaceutical Biotechnology, School of Life Sciences, Nanjing University, Nanjing 210023, China.; ^2^ Nanjing Chengshi Biomedical Technology Co. Ltd., Nanjing 210031, China.

## Abstract

Precise neoepitope discovery is crucial for effective cancer therapeutic vaccines. Conventional approaches struggle to build a repertoire with sufficient immunogenic epitopes. We developed a workflow leveraging full-length ribosome–nascent chain complex–bound mRNA sequencing (FL-RNC seq) and artificial intelligence–based predictive models to accurately identify the neoepitope landscape, especially large-scale transcript variants (LSTVs) missed by short-read sequencing. In the MC38 mouse model, we identified 22 LSTV-derived neoepitopes encoded by a synthesized mRNA lipid nanoparticle vaccine. As a standalone therapy and combined with anti–PD-1 immunotherapy, the vaccine curbed tumor progression, induced robust T cell–specific immunity, and modulated the tumor microenvironment. This underscores the multifaceted potentials of LSTV-derived vaccines. Our approach expands the neoepitope source repertoire, offering a method for discovering personalized cancer vaccines applicable to a broader tumor range. The results highlight the importance of comprehensive neoepitope identification and the promise of LSTV-based vaccines for cancer immunotherapy.

## Teaser

The success of cancer therapeutic vaccines hinges on precise and efficient discovery of tumor neoepitopes.

## INTRODUCTION

Personalized cancer therapeutic vaccines have yielded encouraging results in several ongoing clinical trials and may represent a promising strategy for cancer immunotherapy (*1*–*3*). Neoepitopes, immunogenic parts recognized by T cell receptors (TCRs) on neoantigens formed by tumor-specific mutations, are pivotal for developing cancer vaccines. Their specificity stems from their tumor-exclusive presence, reducing the likelihood of an immune response against normal tissues and therefore could overcome the conventional obstacles of immune tolerance. This unique feature makes neoepitopes the most “favorable” immunogenic targets of T cell (*4*). Personalized cancer vaccines based on neoepitopes have demonstrated good safety and efficacy profile. Neoepitopes act not only as potential therapeutic targets but also as useful biomarkers to monitor and predict the efficacy of immunotherapies.

Clinical studies in recent years have revealed the importance of precise neoepitope identification in eliciting a strong tumor-specific T cell response in patients with favorable therapeutic outcomes, including partial response and even complete response (*5*, *6*). However, the neoepitope identification methods in these clinical studies were primarily tailored for cancers with high tumor mutation burden (TMB), such as melanoma and pancreatic ductal adenocarcinoma (PDAC) (*3*, *7*). Such methods are obviously not ideal for cancers with low TMB. For instance, neoepitope vaccines against glioblastoma have yielded suboptimal results with low response rates (*8*). Traditional short-read sequencing techniques are considered unable to identify sufficient neoepitopes in low TMB tumors (*9*), which largely limit effective vaccine development across multiple cancer indications. Therefore, establishing more powerful neoepitope discovery method is imperative for broadening the application of cancer vaccines to cancers with low TMB.

An intricate mosaic of genomic aberrations gives rise to neoantigens, of which immunogenic parts presented by major histocompatibility complex (MHC) and recognized by TCRs are neoepitopes. Previous studies mainly focused on somatic mutations such as small single-nucleotide variations (SNVs) and small insertions/deletions based on short-read sequencing methods (*10*), which have intrinsic drawbacks on detecting mutations on longer sequence scope. In addition, a considerable portion of neoepitopes originates from unannotated proteins, a type of brand-new proteins that may result from alternative splicing, nontraditional start sites, or read-through translation events and have not been documented yet in current protein databases (*11*, *12*). Unannotated proteins represent a more complex landscape of somatic mutations and can contribute to the diversity of potential neoepitopes for immunotherapy targets. Consequently, the spotlight has now shifted toward innovative methods that can seamlessly capture this broader spectrum of mutations. Full-length ribosome–nascent chain complex–bound mRNA sequencing (FL-RNC seq) allows for the identification of mRNA transcripts that are actively being translated by ribosomes, enabling researchers to achieve a more holistic alignment with the proteome and mitigating the inaccuracies originated from selective translation events (*13*, *14*). Moreover, FL-RNC seq can accurately identify the large-scale transcript variants (LSTVs) originating from large insertions, deletions, or rearrangements in both the coding sequence of a gene and noncoding region. Therefore, FL-RNC seq is a valuable tool for breaking the bottleneck of neoepitope discovery. We applied FL-RNC seq on tumor tissues and matched control tissues from the MC38 mouse syngeneic model and identified a pool of LSTV, based on which we further predicted MHC-I– and MHC-II–restricted epitopes using computational predictive tools named FIONA2.

We developed an mRNA therapeutic vaccine based on selected LSTV sequences rich in MHC-I– and MHC-II–restricted epitopes. The mRNA vaccine formulated as mRNA lipid nanoparticle (LNP) elicited notable cellular immune response and demonstrated strong tumor inhibition efficacy. When used in synergy with PD-1 blockade, the efficacy was further augmented, suggesting a potential paradigm shift in colorectal cancer therapy. Our findings also suggest that immunogenic MHC class II–restricted neoepitopes may dictate the vaccine efficacy. Further investigation using advanced techniques such as single-cell RNA sequencing (scRNA-seq) unveiled notable rejuvenation in the tumor microenvironment after vaccination. LSTV-based mRNA vaccine generated a durable T cell memory response and prevented tumor relapse efficiently. These findings unveiled that the potential of mRNA vaccines based on LSTV-derived neoepitopes may have substantial implications on the development of personalized RNA vaccine for human cancers.

## RESULTS

### LSTVs serve as crucial sources of tumor neoepitopes

**Fig. 1.**
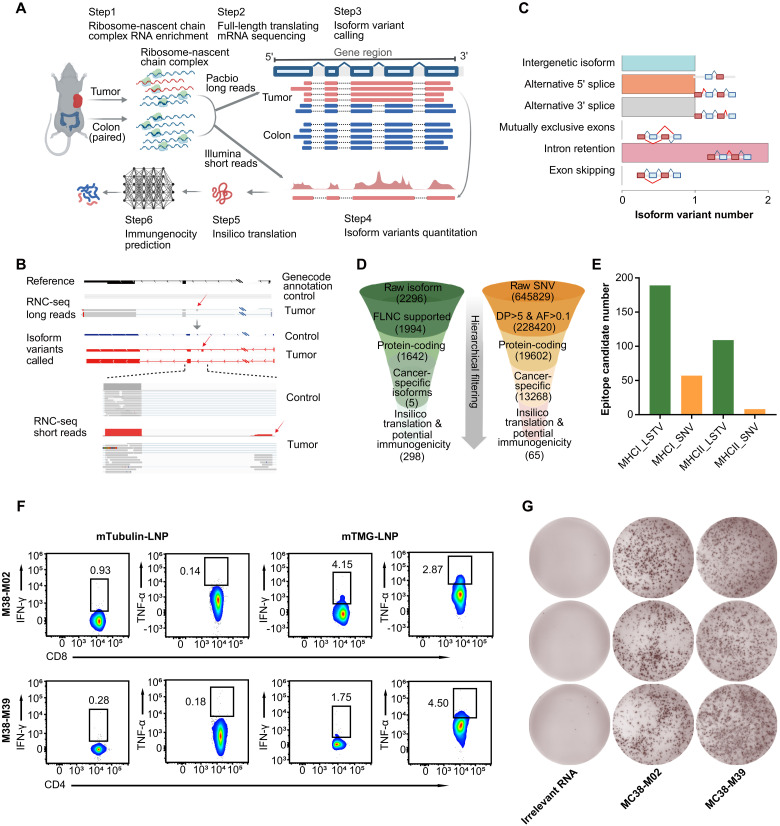
LSTVs identified from long-read RNC-seq contain abundant neoepitopes. (**A**) Pipeline for rapid tumor neoepitope identification through the integration of comprehensive RNC-seq. This innovative approach involves full-length transcript profiling from both tumor and matched normal tissues, combining long-read and short-read sequencing technologies. (**B**) A representative isoform variant, PB.1528.1, identified through long-read RNC-seq, revealed an additional exon (indicated by the red arrow) in the tumor sample compared to the reference annotation. (**C**) In the context of the MC38 allograft tumor model, the identified isoform variants exhibited a diverse distribution spanning four distinct categories: intergenic isoforms, alternative 5′ splice variants, alternative 3′ splice variants, and intron retention events. Notably, no instances of exon skipping or mutually exclusive exons were detected. (**D** and **E**) Comparative analysis of the quantity of potentially immunogenic epitopes derived from SNVs versus LSTVs was performed using identical prediction algorithms. (**F** and **G**) Splenocytes from MC38 tumor-burdened mice immunized with antigen-encoding RNA vaccines (mTMG-LNP) or irrelevant controls were evaluated for the neoepitope-specific T cells on day 21. Flow cytometry analysis (F) examined IFN-γ^+^ or TNF-α^+^ cells within the CD8^+^/CD4^+^ T cell population (*n* = 5 mice), while ELISpot assay (G) detected IFN-γ secretion from splenocytes restimulated with representative neoepitopes MC38-M02 and MC38-M39 (*n* = 3 mice).

To determine tumor suppression potential of neoepitopes generated from LSTVs, we selected MC38 tumor model because previous studies have provided ample evidence that MC38 mice have several well-defined neoepitopes originating from point mutations detected by short-read sequencing (*9*). This allows us to simultaneously use LSTV-derived neoepitopes for comparison in terms of quantity and quality. We harvested tumors from C57BL/6J mice subcutaneously inoculated with MC38 cells. Ribosome–nascent chain complexes (RNCs) were enriched from these tumors and matched normal tissues, enabling comprehensive neoepitope identification through RNC-sequencing (RNC-seq) (*15*). Data analysis was performed using the GRCm39 genome and transcripts as references. Our bioinformatics pipeline (Fig. 1A) identified a total of 2296 raw transcripts isoforms, with 1994 full-length nonchimeric transcripts and 131 unannotated protein-encoding isoforms. Comparative analysis with normal tissue revealed five tumor-specific LSTVs: PB.1596.5, PB.349.2, PB.458.1, PB.1517.1, and PB.1528.1 (fig. S1A and tables S1 and S7).

A meticulous manual case-by-case review of the identified LSTVs revealed an intriguing feature of the PB.1528.1 isoform variant, which presents an extra exon not found in standard Genecode annotations or normal colorectal transcript, indicating its tumor-specific presence (Fig. 1B). This extra exon originally belonged to the intron region, thus categorized as intron retention. Similar analysis conducted for the remaining transcripts revealed two examples of intron retention and one each of intergenic isoform, alternative 5′ splice, and alternative 3′ splice, with no exon skipping or mutually exclusive exons detected (Fig. 1C). Further, the transcriptional expressions of these LSTVs were validated by quantitative polymerase chain reaction (qPCR) analysis (fig. S1, C to G, and table S1) and exhibited similar patterns as observed for RNC-seq.

For the prediction of neoepitopes derived from these LSTVs, we used FIONA2, a machine learning tool tailored for mouse MHC genotypes (H2-Kb, H2-Db, IA-b). This optimized version improved performance of antigen presentation prediction compared to its original version FIONA (*16*) especially in mouse. Finally, a total of 298 epitopes were predicted based on the five identified LSTVs, with 189 epitopes for MHC-I and 109 for MHC-II, respectively (Fig. 1D).

To compare with small-scale variants, such as single-nucleotide variants (SNVs), we used next-generation sequencing data from the same sample. After aligning to the reference genome and obtaining the BAM file, we used VarDict (*17*) for variant calling, yielding 645,829 raw SNVs. Similar to the LSTVs, hierarchical filtering was applied, resulting in a total of 65 potential epitopes, including 57 MHC-I and 8 MHC-II epitopes (Fig. 1E). Compared to SNV, LSTV provided much larger amounts of epitopes, implying that LSTVs may harbor a wealth of tumor-specific neoepitopes. To validate the immunogenicity of the predicted epitopes derived from LSTVs, a tandem minigen mRNA (mTMG) vaccine was designed, which encompassed a total of 49 epitopes, comprising 32 MHC-I and 17 MHC-II epitopes. Notably, M38-M02 and M38-M39 exhibited potent immunogenic responses (fig. S3), thus selected as representative MHC-I and MHC-II epitopes for further investigation. T cell response was determined by cytokine release assay (Fig. 1F), and immunogenic epitopes were identified by interferon-γ (IFN-γ) enzyme-linked immunospot (ELISpot) of splenocytes (Fig. 1G). Consequently, the epitopes originated from LSTVs were highly immunogenic and elicited robust antigen-specific T cell responses. To address the robustness of the immune response induced by LSTV, we performed experiments evaluating the immunogenicity of LSTV-derived neoepitopes against classical SNV neoepitopes using the same mRNA-LNP delivery in the MC38 model. Our results demonstrated that LSTV-derived epitopes conferred superior tumor control compared to the SNV-derived epitopes, illustrating the potential of LSTV-derived epitopes in eliciting potent anticancer immunity (fig. S18). Collectively, these data suggested that LSTVs may serve as crucial potential sources of abundant neoepitopes.

### FIONA2 enables precise prediction of presented antigen, which may contain neoepitopes based on LSTVs obtained from RNC long-read sequencing

**Fig. 2.**
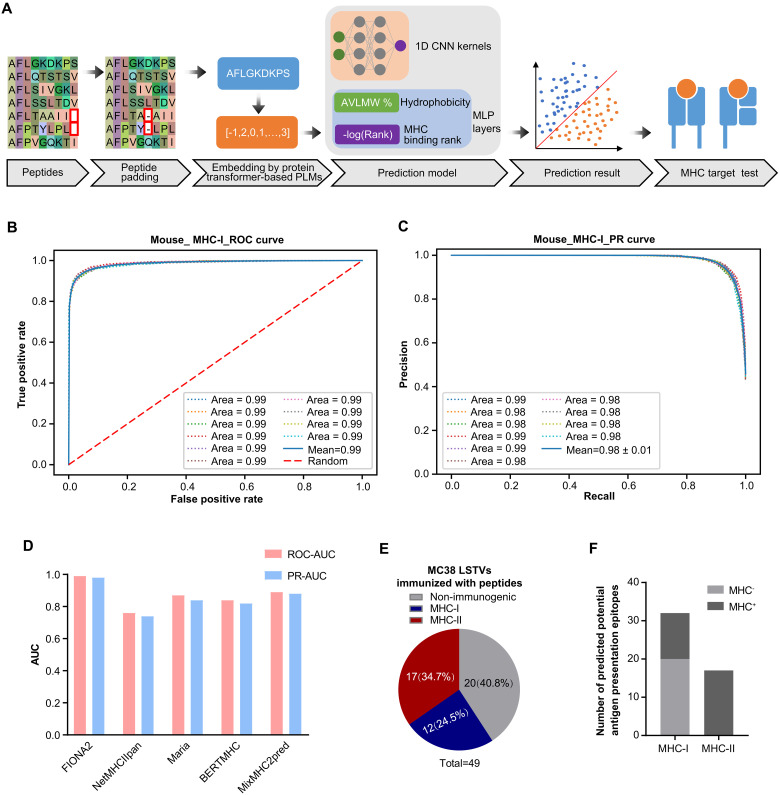
An original algorithm FIONA2 enables precise prediction of neoepitopes based on LSTVs obtained from RNC long-read sequencing. (**A** to **C**) Workflow of the MHC epitope prediction model FIONA2. Peptides are uniformly padded to a consistent length and embedded at contact positions using a transformer-based PLM. For prediction, the model integrates 1D CNN kernels, MHC binding affinity, and hydrophobicity features through an MLP architecture, enabling the generation of MHC binding rank and the subsequent epitope prediction through MLP layers. The schematic diagram of method (A). (B) The ROC curve depicts the performance of the model on unbalanced data for mouse MHC-I epitope prediction. (C) The PR curve evaluates the model’s performance on unbalanced data for mouse MHC-I epitope prediction. (**D**) Comparison of the area under the ROC curve (ROC-AUC) and area under the PR curve (PR-AUC) for FIONA2 and other prediction tools across available MHC-II subtypes. (**E**) The 49 peptides predicted from the MC38 LSTVs were validated one by one by ELISpot assays. The pie chart represents the prevalence of validated immunogenic neoepitopes, including nonimmunogenic, MHC-I–restricted, or MHC-II–restricted neoepitopes. (**F**) Bar graph depicting the number of potential antigen presentation epitopes predicted by the FIONA2 model for MHC-I and MHC-II, with the potentially immunogenic peptides shown as (MHC^+^) and the remaining epitopes as (MHC^−^).

To improve the accuracy of epitope prediction, we developed an improved algorithm named FIONA2 by incorporating a transformer-based model for peptide-MHC presentation prediction on the data basis of FIONA (*16*) and algorithm structure of TRAP (*18*) (Fig. 2A shows the entire workflow). In brief, peptides are padded to the same length and then represented at contact positions by transformer-based pretrained language model (PLM) amino acid embeddings. Also included is a model of one-dimensional (1D) convolutional neural networks (CNNs) with MHC binding rank and hydrophobicity as multilayer perceptron (MLP) for training and prediction. The ROC (receiver operating characteristic) curve and PR (precision and recall) curve of mouse unbalanced data [area under the curve (AUC) = 0.99, PR = 0.98] (Fig. 2, B and C) show effectively high performance. To test the ability of the presentation prediction of several existing MHC-II epitope tools [Maria (*19*), NetMHCIIpan4.0 (*20*), BERTMHC (*21*), and MixMHC2pred (*22*)], we used an independent dataset from the University of Tübingen (*23*) that contains 142,625 naturally eluted ligands from 29 tissues across 42 MHC-II subtypes. The independent dataset is deduplicated by sequence and the corresponding MHC-II subtypes compared with the training dataset. All the supported MHC-II subtypes that overlap the MHC-II subtypes of the independent dataset are tested. For all tools, our FIONA2 model achieved the best performance for 25 of the 32 MHC-II subtypes, excelling particularly in subtypes with abundant eluted peptides (Fig. 2D).

To validate the accuracy of FIONA2, we designed an mTMG vaccine encoding the predicted antigens. Mice were vaccinated with mTMG-LNP (fig. S2 and table S1), and immunogenic epitopes were identified by IFN-γ ELISpot of splenocytes. Approximately 59.2% of MHC-restricted epitopes identified by FIONA2 were proved to induce neoepitope-reactive cytokine-secreting T cells (Fig. 2E). It is highly consistent with epitopes initially screened by our prediction model for potential antigen presentation (Fig. 2F). All MHC-II predicted epitopes (17 of 17, 100%) elicited robust CD4^+^ T cell responses (fig. S3 and table S2). Together, these data indicated that the algorithm FIONA2 enabled successful prediction of MHC-I and MHC-II epitopes with high accuracy.

### LSTV-based mRNA vaccine demonstrated robust therapeutic efficacy against cancer

**Fig. 3.**
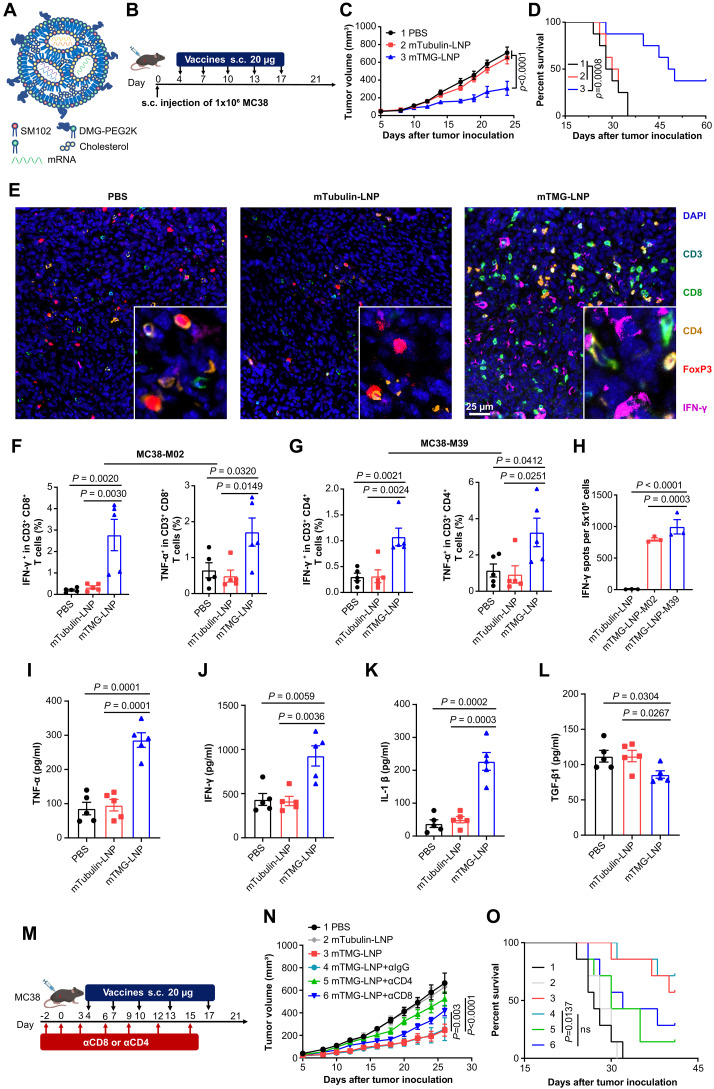
mRNA vaccine incorporating predicted epitopes shows robust tumor control. (**A**) Schematic illustration of the mTMG-LNP vaccine. (**B** to **D**) MC38-bearing female C57BL/6J mice were subcutaneously injected with five doses of neoepitope vaccine [20 μg, subcutaneously (s.c.)] on days 4, 7, 10, 13, and 17 after tumor inoculation (*n* = 8 to 10 mice). Tumor volume was measured every other day. Experimental design (B), tumor growth curve (C), and percentage of survival (D) were shown. (**E**) Tumor tissues were collected on day 21 at the end of the treatment period and multiplex immunohistochemistry investigation of tumor tissues for the following markers: CD3, CD4, CD8, FoxP3, IFN-γ, and DAPI (4′,6-diamidino-2-phenylindole) (*n* = 3 mice). Scale bar, 25 μm. (**F** and **G**) Flow cytometry analysis of IFN-γ^+^ or TNF-α^+^ cells in the CD3^+^CD8^+^ T cell (F) or the CD3^+^CD4^+^ T cell (G) in splenocytes restimulated with MC38-M02 and MC38-M39 antigens (*n* = 5 mice). (**H**) ELISpot assay detected IFN-γ secretion from splenocytes restimulated with representative neoepitopes MC38-M02 and MC38-M39 (*n* = 3 mice). (**I** to **L**) ELISA measurements of proinflammatory cytokine TNF-α (I), IFN-γ (J), and IL-1β (K) and anti-inflammatory cytokine TGF-β1 (L) concentrations in the tumor of the mice (*n* = 5 mice). (**M** to **O**) Average tumor growth curves of specific immune cell subsets (CD8^+^ T cells or CD4^+^ T cells) were depleted to explore their relative contribution to the observed efficacy. Experimental design (M), tumor growth (N), and survival (O) in irrelevant RNA (control) or mTMG-LNP immunized C57BL/6J mice (*n* = 7 mice) inoculated subcutaneously with MC38. Data were shown as mean ± SEM. Statistical analysis was performed by two-way ANOVA [(C) and (N)], one-way ANOVA [(F) to (I)] with Tukey’s multiple comparisons test, and log-rank test [(D) and (O)]. ns, no significance.

To confirm the therapeutic efficacy of neoepitopes predicted by FIONA2, we designed an mTMG vaccine encapsulated by LNP (Fig. 3A). The therapeutic efficacy of neoantigen mRNA vaccines were first investigated in an MC38 mouse model. Mice were vaccinated on days 4, 7, 10, 13, and 17 after tumor cell inoculation (Fig. 3B). As shown in Fig. 3 (C and D) and fig. S4, mRNA vaccine mTMG-LNP significantly inhibited tumor growth and prolonged survival time compared with phosphate-buffered saline (PBS) control and mTubulin-LNP, which contains irrelevant RNA. Impressively, a subcutaneous regimen of five doses of the neoantigen vaccine led to tumor elimination in about 30% of the mice.

Vaccine-induced CD8^+^ and CD4^+^ T cell responses were analyzed by fluorescent multiplex immunohistochemistry (mIHC) and flow cytometry. In the vaccine-treated group, both CD8^+^ and CD4^+^ T cells increased at the tumor site, with an elevated secretion of the cytotoxic mediator IFN-γ. Notably, the FoxP3/CD4 ratio was notably reduced after vaccination when compared to the irrelevant control (Fig. 3E and fig. S15), indicating the elicitation of a tumor-specific adaptive immune response after vaccination. Previous studies have linked the proliferation of peripheral effector T cells to favorable outcomes in immunotherapy, and an increase in tumor-specific CD8^+^ and CD4^+^ effector T cells within peripheral tissues is advantageous for inhibiting tumor progression (*24*). To evaluate the induction of peripheral tumor-specific T cells by vaccine treatment, we investigated splenocytes’ reactivity to MHC-I–restricted peptide–MC38-M02 and MHC-II–restricted peptide–MC38-M39, respectively, via flow cytometry. Consistent with the mIHC studies, fluorescence-activated cell sorting (FACS) analysis indicated an increase in cytotoxic T cells by vaccine treatment (Fig. 3, F and G). ELISpot analyses demonstrated that mTMG-LNP resulted in a strong increase in the numbers of IFN-γ^+^ cytotoxic T lymphocytes and IFN-γ production (Fig. 3H and fig. S3), confirming the potent cellular immune response induced by mTMG-LNP.

To assess the immune responses within tumors, we first performed vaccine treatment on murine colorectal tumor model MC38 and enzyme-linked immunosorbent assay (ELISA) assays are used to measure cytokine levels in the tumor microenvironment. Treatment with mTMG-LNP notably increased levels of proinflammatory cytokines [tumor necrosis factor–α (TNF-α), IFN-γ, and interleukin-1β (IL-1β)] (*P* = 0.0001, *P* = 0.0036, and *P* = 0.0003, respectively) (Fig. 3, I to K) and decreased the anti-inflammatory cytokine transforming growth factor–β1 (TGF-β1) (Fig. 3L), compared to controls. These changes suggest that mTMG-LNP could shift the tumor environment toward an antitumor profile by enhancing both innate and adaptive immune responses. As both MHC-I and MHC-II epitopes could shape vaccine-induced antitumor immunity, we sought to demonstrate their respective contributions to the antitumor immune response elicited by vaccine. By using monoclonal antibodies for CD8^+^ or CD4^+^ T cell depletion (fig. S6), we observed that while CD8^+^ T cell depletion blunted approximately 48% antitumor effects of vaccination, CD4^+^ T cell depletion resulted in around 82% decrease of vaccination response, further highlighting the importance of MHC-II neoepitopes in shaping antitumor immunity (Fig. 3, M to O, and fig. S5C). These data are in accordance with previous reports that showed that tumor rejections during immunotherapy require the presence of both immunogenic MHC-I and MHC-II neoepitopes (*25*). Together, these data demonstrate combinatorial effects of CD4^+^ and CD8^+^ T cells on the antitumor immune response during vaccine treatment.

### scRNA-seq analysis revealed reinvigoration of tumor microenvironment by LSTV-based mRNA vaccine administration

**Fig. 4.**
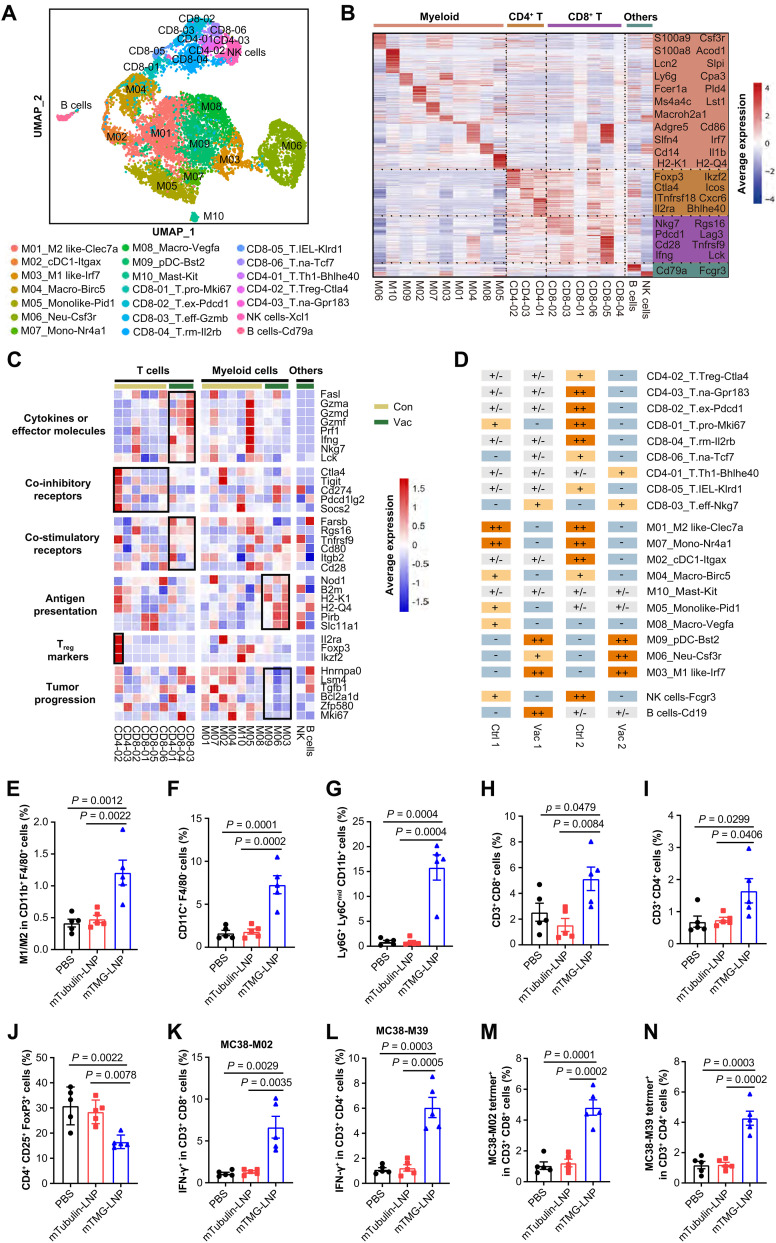
scRNA-seq analysis revealed remodeling of tumor microenvironment following mRNA vaccine administration. (**A**) UMAP plot showing Seurat-guided unsupervised clustering and distribution of 16,945 CD45^+^ cells from the tumor tissue of MC38-bearing mice (*n* = 4 mice). Each dot denotes an individual cell; same color indicates the same cluster. There are 21 main clusters, including 6 CD8^+^ clusters, 3 CD4^+^ clusters, 10 myeloid cell clusters, and another 2 smaller clusters of immune cells, NK cells, and B cells. We selected one representative signature gene to name each cluster and indicate the potential function (bottom). (**B**) Heatmap displaying normalized expression values of differential gene sets for myeloid cells, CD4^+^ T cells, CD8^+^ T cells, NK cells, and B cells in tumor tissue. (**C**) Heatmap showing the average expression of representative cell function–related genes in five major cell clusters in the different treatment groups. (**D**) Sample preference of each cluster estimated by odds ratios (ORs) (Materials and Methods). ++ (ORs ≥ 3, *P* < 0.05) represents highly enriched; + (1.5 ≤ ORs < 3, *P* < 0.05) represents enriched; +/− (0.5 ≤ ORs < 1.5, *P* > 0.05) represents nonsignificant; − (0 < ORs < 0.5, *P* < 0.05) represents not to distribute in tissue. (**E** to **J**) Flow cytometry analyses of the ratio of M1/M2 in CD11b^+^F4/80^+^ cells (E) and CD11C^+^F4/80^−^ cells (F) and Ly6G^+^Ly6C^mid^ CD11b^+^ in CD45^+^ cells (G), CD8^+^ T cells (H), and CD4^+^ T cells (I) in CD3^+^ T cells and the ratio of CD25^+^FoxP3^+^ in CD4^+^ cells (J) in tumor from immunized mice. (**K** to **N**) T cell responses in TILs restimulated with MC38-M02 (K) and MC38-M39 (L) peptides were measured by flow cytometry. The percentage of neoepitope-specific T cells in TILs was detected by MC38-M02 (M) and MC38-M39 (N) tetramer staining (*n* = 5). Data were shown as mean ± SEM. Statistical analysis was performed by one-way ANOVA [(E) to (N)] with Tukey’s multiple comparisons test.

To comprehensively understand the immune networks within the tumor microenvironment induced by vaccination, we collected CD45^+^ cells by fluorescence-activated cell sorting (FACS) from tumors of control and vaccinated mice. scRNA-seq analysis was performed via a droplet-based 10x Genomics platform to obtain the transcriptomes of individual cells. After quality control and filtering, 16,945 transcriptomes of single cells were identified and applied for graph-based clustering (*26*). Ultimately, we identified 21 different cell types and states within the immune cell populations, including T cells, myeloid cells, natural killer (NK) cells, and B cells (Fig. 4A, fig. S12, and data S1). Heatmap showed differentially expressed genes (DEGs) in each cell cluster (Fig. 4B). The immune cell clusters that exhibit high similarities were confirmed as the same subsets. All cell clusters could be visualized by a combined Uniform Manifold Approximation and Projection (UMAP) analysis.

For defining tumor-infiltrating lymphocytes (TILs), six CD8^+^ T cell subsets (CD8-01 to CD8-06) were identified, including proliferative (T.pro), exhausted (T.ex), effector (T.eff), residential memory (T.rm), and naïve (T.na), given their specific expression of Mki67, Pdcd1, Gzmb, Klrd1, Il2rb, and Tcf7 genes, respectively. Three CD4^+^ T cell subsets (CD4-01 to CD4-03) were identified, further distinguished by the specific expression of Cxcr6, FoxP3, and Gpr183 genes and annotated as T helper 1 (T_H_1), regulatory T (T_reg_), and naïve CD4^+^ T cell population, respectively (Fig. 4A and fig. S13). Of note, both CD8^+^ T.eff and CD4^+^ T_H_1 cells exhibited high expression of effector molecules or cytokines, such as Gzma, Ifng, Prf1, and Lck (Fig. 4C), and costimulatory molecules (Rgs16 and Tnfrsf9), indicating that MHC-I and MHC-II neoepitopes involved in cancer vaccine elicited robust CD8^+^ and CD4^+^ T cell responses. With the induction of CD4^+^ T cells after vaccination, activation of T_reg_ cells was concerning. To our surprise, enrichment of T_reg_ cells and expression of their effector molecules (Ctla4, Tigit, etc.) were drastically decreased in the tumors of vaccinated mice, a phenomenon also observed in patients receiving cancer vaccine (*27*). T.ex population was less enriched in vaccinated tumors in comparison to control (Fig. 4D and data S2), showing rejuvenation of immune response after vaccination, which is possibly due to the decreased suppression by T_reg_ cells and some other immunosuppressive cells. Nevertheless, these data collectively demonstrated robust induction of an effective CD8^+^ and CD4^+^ T cell immune response by cancer vaccine.

In addition to lymphocytes, the functional status of myeloid cells was markedly altered following vaccination and thus broadly defined. We identified 10 myeloid cell clusters, among which 4 clusters relate to macrophage (M01, M03, M04, and M08). These macrophage clusters were characterized by high expression of Itgam and further annotated as M1-like, M2-like, M04-macro, and M08-macro for their specific expression of Clec7a, Irf7, Birc5, and Vegfa genes (fig. S11). While protumor M2-like macrophages were highly enriched in unvaccinated tumors, antitumor M1-like macrophages were preferentially enriched after vaccination, showing induction of a phenotypic switch in macrophage polarization by cancer vaccine. Notably, SPP1^+^ macrophages, a subset that primarily mediates protumor effects, considerably declined after vaccine, further supporting the functional switch of tumor-associated macrophages (*28*).

Two dendritic cell (DC) subsets (M02_cDC1 and M09_pDC) characterized by high expression of CD11c were identified and further distinguished by the specific expression of Itgax and Bst2, respectively. Notably, both M1-like and DC2 subsets exhibit enhanced antigen-presenting capacity. It is likely that these cells with improved antigen presentation capabilities may contribute to the formation of tertiary lymphoid structures (TLSs) within tumors and present tumor-associated antigens released upon immunogenic cell death (ICD) induced by therapeutic vaccination (*29*). Corroborating this hypothesis, we observed colocalization of T cells, B cells, and DCs within TLS in the tumor (fig. S14), implying that these antigen-presenting cells may reside within TLS and facilitate the presentation of tumor antigens, thereby triggering antitumor T cell immune response to cancer vaccine.

Among the myeloid clusters, neutrophils seem to be the most pronouncedly changed after vaccination. Of note, neutrophils robustly enrich after vaccination, largely consistent with previous reports that have demonstrated that successful immunotherapy is associated with acute tumor neutrophil expansion (*30*). These neutrophils might be recruited to eradicate antigen escape variants and cooperate with vaccine-induced antigen-specific T cells to eliminate antigenically heterogeneous tumors.

Overall, scRNA-seq analysis revealed that cancer vaccine induced extensive remodeling of tumor microenvironment, including decreased T_reg_ levels, a switch of macrophage polarization, enhanced antigen-presenting capacity of DC, and neutrophil accumulation. All these signatures would further potentiate vaccine-elicited T cell immune response and exert profound impacts on cancer progression.

To confirm these observations defined by scRNA-seq, we applied flow cytometry to characterize the immune cell subsets within the tumor microenvironment (fig. S10). In line with the findings by scRNA-seq, we observed that the M1 to M2 ratio was significantly increased after vaccine treatment (Fig. 4E), showing the polarization switch of tumor-associated macrophages in response to vaccine. Also increased was DC subsets (Fig. 4F), which may enhance the presentation of tumor antigens locally and expand the TCR repertoire reactive to tumor cells. Activated neutrophils, which are characterized with CD11b^+^Ly6G^+^Ly6C^mid^, showed an increase after vaccination (Fig. 4G). The balance between antitumor T cell response and immunosuppressive T_reg_ cells was remarkably tilted toward an antitumor state after vaccine treatment (Fig. 4, H to J). Both CD8^+^ and CD4^+^ T cells displayed elevated release of IFN-γ after vaccine treatment (Fig. 4, K and L), and tetramer labeling indicated the specificity of the T cell response (Fig. 4, M and N). Together, these data indicated that successful vaccine therapy could not only trigger antigen-specific T cell responses but also create a favorable environment for T cell functioning properly by remodeling tumor microenvironment (TME).

### Clonal dominance in TCR repertoire was shifted after LSTV-based vaccine treatment

**Fig. 5.**
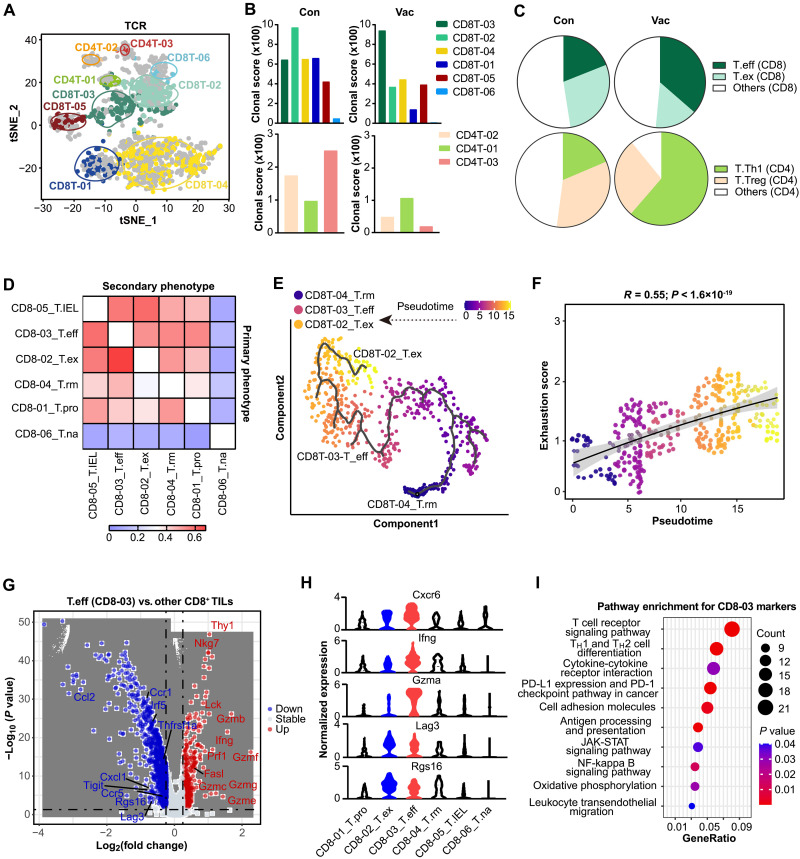
Lineage tracking of clonal T cell subsets and Identification of T cell landscape in response to vaccine treatment. (**A**) The clonal T cells (*n* = 1770 cells from *n* = 4 mice) were highlighted in the t-SNE plot. The enriched clusters are indicated by colored oval. (**B**) Clonal diversity levels of nine tumoral T cell clusters quantified by clonal score for each tumor sample. (**C**) Dynamic changes of inhibitor-like T cell cluster clones and cytotoxicity-like T cell clones in the proportion of other T cell clusters after vaccine treatment. (**D**) Heatmap showing the fraction of T cells with clonotypes belonging to a primary phenotype cluster that are shared with other secondary phenotype clusters. (**E**) Monocle-guided cell trajectory of three major CD8^+^ T cell clusters with high migration relationship. The arrow indicates the direction of the inferred pseudotime. The different functional states identified by monocle were sorted along the artificial pseudotime. (**F**) The inferred pseudotime is correlated with the exhaustion feature of CD8^+^ T cells. The solid line represents the LOESS fitting of the relationship between the pseudotime and exhaustion scores. Dots were colored by their cluster origin. (**G**) Volcano plot showing DEGs between T cells of CD8-03-T.eff and other CD8^+^ tumor-infiltrating T cells. (**H**) Violin plots showing the expression levels of Cxcr6, Ifng, Gzma, Lag3, and Rgs16 across the tumoral CD8^+^ T cell clusters. (**I**) Differential pathways enriched for the discriminative markers of CD8-03 T cell subset by KEGG. *P* values were calculated by using the two-sided Pearson’s correlation coefficient test (F).

To characterize TCR repertoire diversity in TILs after vaccine treatment, we applied single-cell TCR sequencing (scTCR-seq) to CD3^+^ T cells obtained from control and vaccinated tumors. Through coupled scRNA-seq and TCR repertoire analyses at single-cell resolution, we observed an enrichment of effector memory T cell cluster and a decrease of TCR repertoire diversity in both CD8^+^ and CD4^+^ T cells after vaccination (Fig. 5, A to D). In addition, vaccine treatment further skewed clonal dominance in the dynamic TCR repertoire, given the evidence that while unvaccinated tumor showed TCR clonotypes dominated by exhausted CD8^+^ T cells, tumors undergoing vaccination exhibited clonal dominance in effector CD8^+^ T cells. Clonality of CD4^+^ T cells was featured with a significant decrease of T_reg_ cells (Fig. 5C). These clonal fluctuations aligned with the changes observed for corresponding cell clusters by scRNA-seq and flow cytometry analysis. These data collectively suggest that TCR clone diversity declined after vaccine treatment and the clonal dominance tended to be skewed toward effector T cells.

To delineate the transcriptional characteristics of antigen-specific T cells, we performed pseudotime analysis, which revealed three transcriptional trajectories emanating from an activated T cell population based on transcriptional similarities: an effector T cell state, an exhausted T cell state, and a T cell state exhibiting a residential memory (T.rm) signature (Fig. 5, E and F). The T.rm signature likely reflects a natural local immune defense mechanism. To further provide molecular insights into effector T cells responding to vaccine administration, we performed pathway enrichment analysis and found that these cell subsets were characterized by high expression of Cxcr6, Ifng, Gzma, and Rgs16 and enriched with TCR signaling pathway and T_H_1 response (Fig. 5, G to I, and data S5), consistent with the findings as observed by scTCR-seq. Nevertheless, these data collectively indicated that vaccine administration produced a shift in the clonal dominance of TCR repertoire, with a preferential enrichment in effector T cells.

### A long-term immune memory response was elicited by LSTV-based mRNA vaccine in vivo

**Fig. 6.**
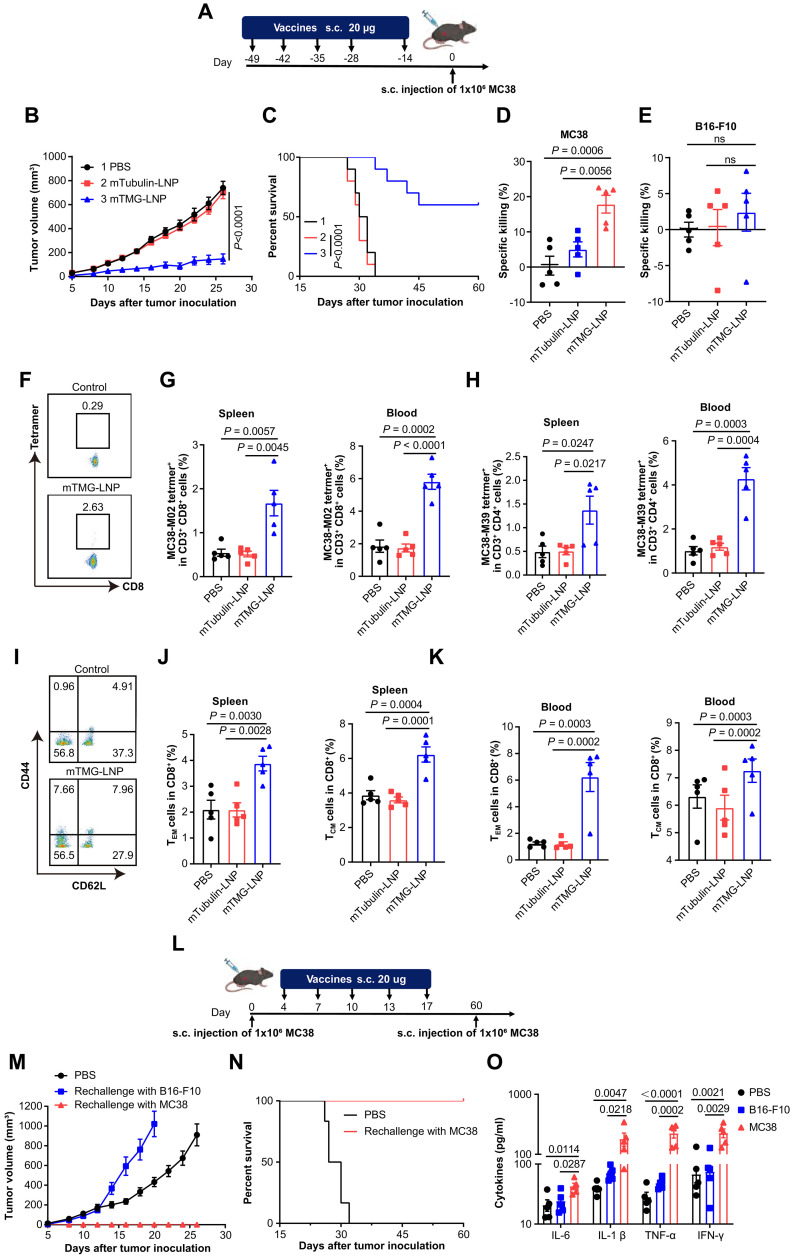
The long-term immune memory in vivo elicited by mRNA vaccine. (**A**) Timeline (days) of vaccination and tumor inoculation in MC38 cancer. (**B** and **C**) Tumor growth curves and survival curves of mice preventatively treated with vaccines for MC38 cancer (*n* = 10). (**D** and **E**) Specific killing ability of splenocytes collected on day 50 toward MC38 cells with multiple-epitope antigen (D) and B16-F10 cells without multiple-epitope antigen (E) analyzed by CCK8 assay. (**F**) Representative scatterplots and the percentage of the tetramer^+^ T cell subsets among the CD8^+^ T cells (%). (**G** and **H**) Quantitative analysis of MC38-M02 tetramer^+^ T cells (G) or MC38-M39 tetramer^+^ T cells (H) in splenocytes and blood on day 60 through flow cytometry (*n* = 5). (**I**) Representative scatterplots and the percentage of the CD44^+^ CD62L^+^ T cell subsets among the CD8^+^ T cells (%). The proportion of effector memory T cells (T_EM_) cells (CD8^+^CD44^+^CD62L^−^) or central memory T cells (T_CM_) cells (CD8^+^CD44^+^CD62L^+^) in splenocytes (**J**) and blood (**K**) on day 50 (*n* = 5). (**L**) Schema of tumor rechallenge model. The mice were inoculated with MC38 cells and treated with mTMG-LNP vaccine. Then, the survived animals (tumor elimination) were rechallenged with subcutaneous injection of B16-F10 and MC38 cells on day 60. Tumor growth curves (**M**) and survival curves (**N**) of mice vaccinated with mTMG-LNP. ELISA measurements of proinflammatory cytokine IL-6, IL-1β, TNF-α, and IFN-γ (**O**) concentrations in the serum of the mice inoculated with B16-F10 or MC38 cells. Data were shown as mean ± SEM. Statistical analysis was performed by two-way ANOVA (B) and one-way ANOVA [(D), (E), (G), (H), (J), (K), and (O)] with Tukey’s multiple comparisons test. ns, no significance. The experiments were performed at least three times.

Establishing robust immune memory is pivotal for the sustained efficacy of cancer vaccines. To evaluate the vaccine-induced memory response, prophylactic protection studies were carried out and the mice were vaccinated on days 14, 28, 35, 42, and 49 before tumor challenge (Fig. 6A). About 60% of the immunized mice remained tumor-free after MC38 tumor cell inoculation in the prophylactic protection study (Fig. 6, B and C, and fig. S5A). On day 50 after inoculation, splenocytes were collected and their cytotoxic effects on B16-F10 and MC38 cells were evaluated. As shown in Fig. 6D, splenocytes from mice immunized with mTMG-LNP showed increased cytotoxicity against MC38 cells compared to those from other groups. No prophylactic protection was observed in mice inoculated with B16-F10 cells that lacked epitopes encoded by the mRNA therapeutic vaccine. These data provided strong evidence for the antigen specificity of the immune response (Fig. 6E and fig. S5B).

Next, we quantified antigen-specific T cells in splenocytes and blood by staining with pMHC tetramers. mTMG-LNP vaccination generated a greater number of antigen-specific T cells (tetramer^+^ T cells) and IFN-γ^+^ cytotoxic T lymphocytes than mTubulin-LNP (Fig. 6, F to H, and figs. S7 and S8). Furthermore, we investigated the vaccine-induced memory T cell response in the lymph nodes, spleen, and peripheral blood using flow cytometry. We noted a sustained elevation in the populations of both CD8^+^ central memory (T_CM_) and effector memory T cells (T_EM_) within the spleen and peripheral blood, persisting over 50 days. These findings suggest that mTMG-LNP vaccines based on LSTV-derived neoepitopes could effectively foster strong and durable immune memory responses (Fig. 6, I to K, and fig. S9).

To further investigate the vaccine-induced immunological memory response, we performed the tumor rechallenge in mice that had previously cleared MC38 cells after mTMG-LNP vaccination. The mice were rechallenged with subcutaneous injection of B16-F10 or MC38 on day 60 (Fig. 6I). Remarkably, mTMG-LNP provided complete protection (100%) when mice were reexposed to MC38 cells (Fig. 6, M and N). Moreover, the mice in the mTMG-LNP group exhibited no detectable tumors over a 90-day period. In contrast, mice inoculated with B16-F10 tumor cells developed tumors reaching up to 1000 mm^3^ after 3 weeks, highlighting the specificity of the mTMG-LNP–driven immune response to the tumor antigens. In addition, the serum levels of proinflammatory cytokines, including IL-6, IL-1β, TNF-α, and IFN-γ, were elevated in the MC38 cell–inoculated mice compared with the B16-F10 cell inoculation group (Fig. 6O). These results collectively demonstrate that mTMG-LNP induces a durable and highly antigen-specific immune memory response.

In the studies conducted on mice, treatment did not result in any notable reduction in body weight, and hematoxylin and eosin staining of various tissues after vaccination revealed no abnormalities (fig. S16). To rigorously assess the safety profile of our vaccine, we selected three representative MHC-I–restricted peptides and predicted peptides with high similarities to self-antigens. The ELISpot assay results indicated that these self-antigen–derived peptides did not induce any detectable immune response, underscoring the favorable safety profile of our mRNA-LNP vaccine (fig. S3 and table S3). Overall, these findings support the reliability of our approach in designing therapeutic vaccines for a broad spectrum of cancers, especially those with low TMB.

### LSTV-based therapeutic mRNA vaccine synergizes with PD-1 blockade for immunotherapy of colorectal cancer

**Fig. 7.**
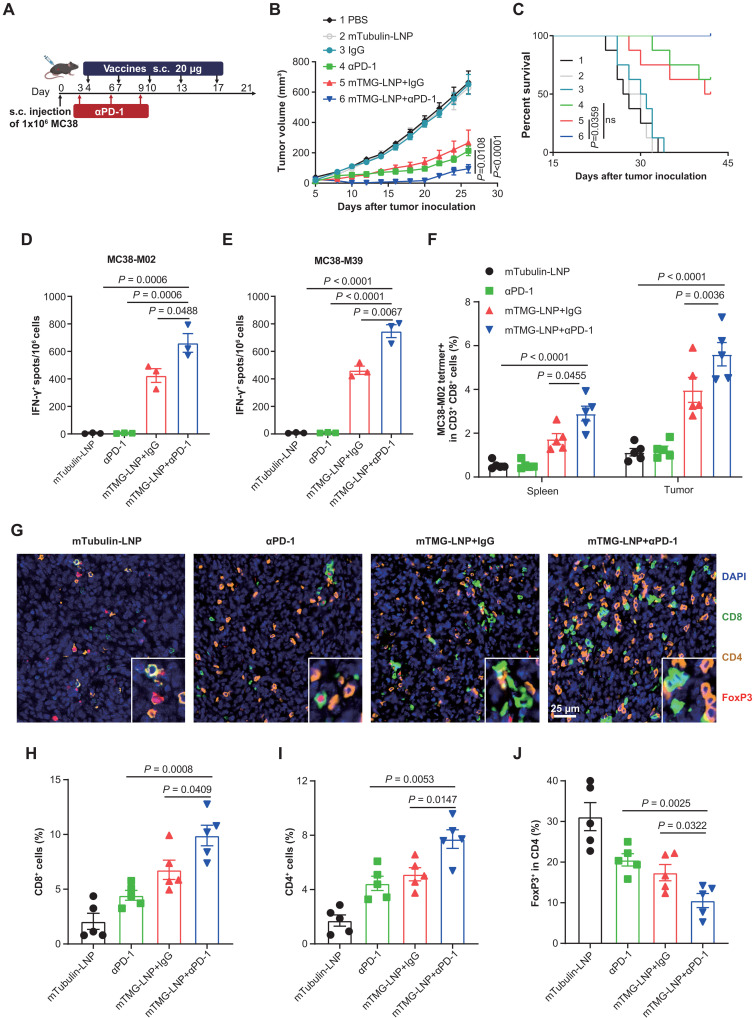
Therapeutic mRNA vaccine synergizes with PD-1 blockade for immunotherapy of colorectal cancer. (**A** to **F**) Female C57BL/6J (*n* ≥ 8) mice were subcutaneously inoculated with 1 × 10^6^ MC38 tumor cells and treated with the combination of neoantigen vaccine and αPD-1. Experimental design (A), tumor growth curve (B), and percentage of survival (C) were shown. [(D) and (E)] T cell responses in splenocytes restimulated with MC38-M02 (D) and MC38-M39 (E) peptides were measured ex vivo via ELISpot (*n* = 3). (F) The percentage of neoepitope-specific CD8^+^ T cells in the tumor tissue was detected by MC38-M02 tetramer staining (*n* = 5). (**G**) Multiplex immunohistochemistry investigation of tumor tissues after combination treatment for the following markers: CD8, CD4, FoxP3, and DAPI. Scale bar, 25 μm. (**H** to **J**) Proportion of infiltrating cells in sections from different treatment groups. Five nonoverlapping visions were randomly selected for each section, with the Halo software (3.5, Indica Labs, USA) automatically collecting positive staining cells and the total number of cells (n = 3 mice); CD8 (H), CD4 (I), and FoxP3 (J) were involved. Data were shown as mean ± SEM. Statistical analysis was performed by two-way ANOVA (B) and one-way ANOVA [(D) to (F) and (H) to (J)] with Tukey’s multiple comparisons test and log-rank test (C).

The neoantigen vaccine primed the immune system to recognize and target tumor-specific antigens, but achieving a complete response remains a challenge. Since PD-1 inhibitors lift the immunosuppression often imposed by tumors and unleash the full effector functions of T cells, we sought to determine the combinatorial effects of mTMG-LNP vaccine and PD-1 blockade. To this end, we combined vaccines with αPD-1 to treat MC38 tumor and achieved synergistic suppression on tumor growth (Fig. 7, A to C, and fig. S5D) and an improved cure rate of ≈40%, underscoring the effectiveness of this combined approach.

To further unravel the intricacies of the resulting immune response after combination therapy, we meticulously characterized the immune phenotypes of antigen-specific T cells. ELISpot analysis revealed that the combination of the vaccine with PD-1 blockade resulted in a substantial increase in IFN-γ release compared to the vaccine alone (Fig. 7, D and E), highlighting the superior efficacy of the combination strategy. Further insights into the antigen-specific T cell population were gained through FACS analysis using tetramer labeling for precise identification. The vaccine alone successfully elicited antigen-specific T cells, and this response was significantly potentiated when combined with PD-1 blockade (Fig. 7F). Perhaps PD-1 blockade induced clonal expansion of antigen-specific T cells, contributing to the observed improvement in the context of combination therapy. Overall, these findings affirmed the role of the combined strategy in fostering antigen-specific immunity, providing a promising avenue for optimizing vaccine-induced immune responses.

To dissect the complex immune microenvironment after combination therapy, we used multiple immunofluorescence staining techniques for immune cell analysis. The combination therapy not only potentiated the infiltration of both CD8^+^ and CD4^+^ T cells but also resulted in a substantial increase in IFN-γ release (Fig. 7, G to I). This combination approach led to a notable decrease in FoxP3^+^ T_reg_ cells, suggesting a favorable shift in the immune microenvironment toward a more proinflammatory state (Fig. 7J). These results collectively support the rationale behind combining the vaccine with PD-1 blockade, which demonstrated enhanced antigen-specific responses and a favorable modulation of the immune landscape. This therapeutic regimen has demonstrated favorable safety and efficacy profile in a phase Ib clinical trial (*1*), making it a promising strategy for the treatment of advanced solid tumors. We would speculate that LSTVs can expand the neoepitope pool personally identified in patients and thereby potentiate the clinical efficacy of the combined regimen for the treatment of solid tumors.

### Personalized vaccine-induced effector T cell gene signatures are associated with better clinical outcomes in human cancers

**Fig. 8.**
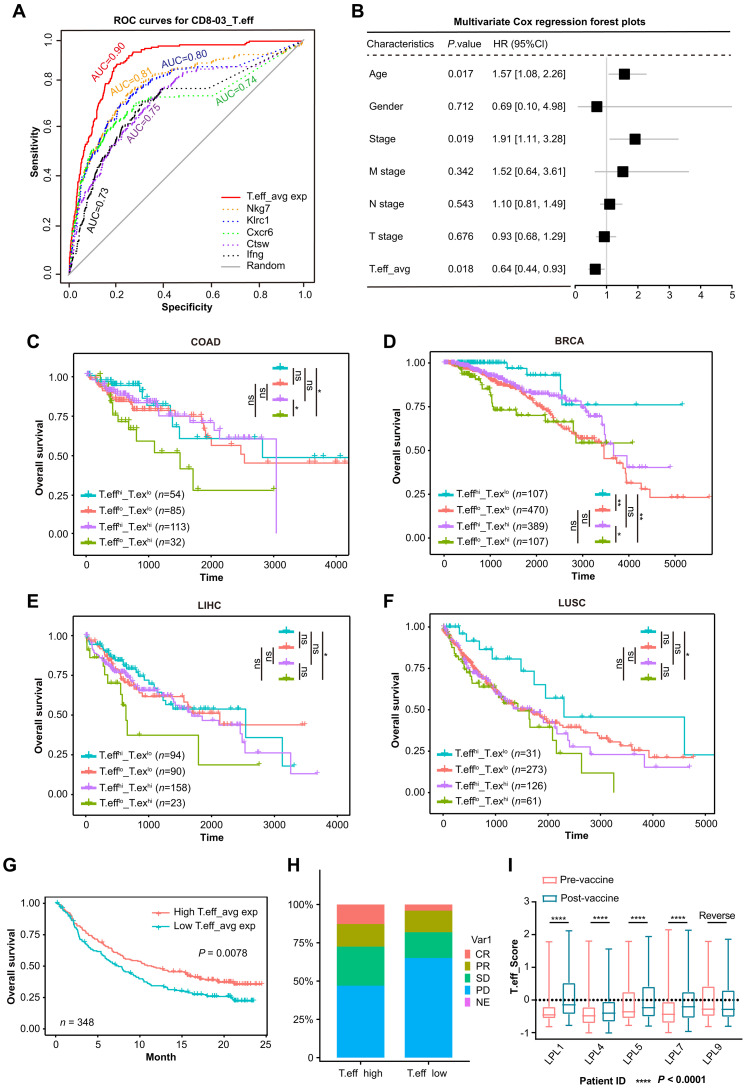
The discriminative marker of antigen-specific T cells are associated with better survival in human tumors. (**A**) ROC plots showing the performance in prediction of CD8-03 effector T cells. (**B**) A multivariate Cox regression analysis was conducted on the BRCA dataset, incorporating the average expression of representative marker genes from CD8-03 T.eff cells (with five selected genes serving as representatives based on their average expression to signify this cell population) alongside common factors such as gender, age, and TNM staging. (**C** to **F**) Kaplan-Meier survival curves of patients from colon adenocarcinoma (COAD, *n* = 284) (C), breast cancer (BRCA, *n* = 1073) (D), liver cancer (LIHC, *n* = 365) (E), and lung squamous cell carcinoma (LUSC, *n* = 491) (F) with respect to high or low T.eff/T.ex score within tumor specimens. (**G**) Survival analyses for low and high average expression of T.eff patient groups in the anti–PD-L1 immunotherapy cohort using Kaplan-Meier curves (IMvigor210 cohort). (**H**) Bar plots show that patients with high expression of T.eff are more likely to respond to anti–PD-L1 treatment. Patients expressing low levels of T.eff showed a low response [complete response (CR)] and more progressive disease (PD) than patients with low average expression of T.eff. (**I**) Analysis of data from a first-in-human clinical trial evaluating therapeutic DNA idiotype neoantigen vaccine in nine patients with asymptomatic LPL. Four of five LPL patients showed higher T.eff scores of CD8^+^ T cells in post-tumor vaccine samples when compared to pretreatment counterparts [GSE243545, one-sided Wilcoxon rank sum test, LPL1 (*P* = 2.6 × 10^−15^), LPL4 (*P* = 1 × 10^−16^), LPL5 (*P* = 1 × 10^−15^), LPL7 (*P* = 1 × 10^−16^), LPL9 (*P* = 1 × 10^−15^)]. Reverse indicates the opposite T.eff score change. Survival distributions were compared using the log-rank test [(C) to (F)]. *P < 0.05, **P < 0.01, ****P < 0.0001.

To explore if the personalized vaccine-induced effector T cell gene signature in the murine MC38 model could be translational in human cancers, we first defined the specific gene signature associated with the personalized vaccine-induced T cells. Specifically, we selected DEGs for CD8-03, which are homologous to the human genome. Using each DEG as a predictor of CD8-03 against all other CD8^+^ T cells, we performed supervised feature selection. The average expression of the top 5 most discriminative markers achieved a high AUC of 0.902 (Fig. 8A). Consequently, we defined the average expression of these five markers as vaccine-induced effector T cell score (T.eff), serving as a surrogate indicator of the proportion of vaccine-induced effector T cells. Similarly, we defined the average expression of another set of five markers as the vaccine-induced exhausted T cell score (T.ex), a surrogate indicator of the proportion of vaccine-induced exhausted T cells (fig. S17A). Then, we estimated the scores of the five signature genes and T.eff in the BRCA database of human colorectal cancer and selected common sex and stage factors to investigate their prognostic value. Hazard ratios for the five characterized genes and T.eff scores were estimated using multivariate Cox models with common gender, age, and tumor node metastasis (TNM)-staging factors. We found that T.eff scores had lower risk representation in the human colorectal cancer BRCA database (Fig. 8B and fig. S17B). The T.eff/T.ex score significantly predicts survival benefit in patients with colon cancer, breast cancer, liver cancer, and lung squamous cell carcinoma. The overall survival was longer in the T.eff^high^/T.ex^low^ score population compared with the T.eff^low^/T.ex^high^ score population (Fig. 8, C to F, and fig. S17, C to F). Therefore, the discriminative features of T cells induced by personalized vaccination in mice may have clinical relevance in human cancer and correlate with favorable prognosis.

Since most The Cancer Genome Atlas samples were not treated with immunotherapy, we next investigated the performance of the T.eff score using human samples subjected to immune checkpoint blockade. A recent study reported that non-small cell lung cancer showing a complete pathological response to immunotherapy exhibits a stronger immune infiltrate, characterized by higher levels of IFNG, GZMB, NKG7, and M1 macrophages (*31*). Effector T cells (CD8-03) up-regulated several cytotoxic markers (Fig. 5G), potentially indicating genes predictive of immunotherapy effectiveness. To test whether T.eff score predicts the efficacy of immunotherapy in human tumors, we analyzed a public dataset of patients with advanced bladder cancer (BLCA) before and after anti–PD-1 therapy. T.eff scores were significantly correlated with immunotherapy prognosis (Fig. 8, G and H). Furthermore, tumor vaccine treatment increased the T.eff scores in four of five lymphoplasmacytic lymphoma (LPL) patients (Fig. 8I). Consistently, three-fifths of these patients who received the cancer vaccine experienced reduced T.ex scores (fig. S17G), confirming the role of immunotherapy in expanding personalized vaccine-induced T cells in the TME.

## DISCUSSION

Personalized cancer therapeutic vaccines offer a tailored approach as neoepitopes serve as precise targets across multiple cancer types and may represent an optimal solution of next-stage cancer immunotherapy. These vaccines circumvent immune tolerance and off-target effects, leveraging mRNA’s flexibility and ease of use to swiftly satisfy individual patient needs (*32*, *33*). Despite promising results of early trials in high TMB cancers like melanoma and PDAC, broadening their application to cancers with low TMB remains challenging (*34*). One straightforward approach to enhance vaccine efficacy is integrating a broader and more precise spectrum of cancer-specific neoepitopes to elicit stronger cellular immune response, cover a greater proportion of tumor cells to address tumor heterogeneity, and reduce the risk of immune evasion during subsequent treatment processes.

Cancer cells are inevitably associated with genomic variations, such as inversions, translocations, deletions, and insertions. Genomic variations may profoundly assist tumor cell growth and proliferation, but may also create LSTVs that could provide immunogenic neoepitopes for immunotherapy targets (*11*, *35*, *36*). Specifically, LSTVs could originate from intergenic isoform, alternative splicing, intron retention, and exon skipping, with a preference for intron retention. Our study further revealed that LSTVs can provide a larger repertoire of potential MHC-I– and MHC-II–restricted epitopes, nearly four to five times more than SNVs in the case of MC38 tumor. These findings suggest that LSTVs might provide a major source of neoepitopes for immunotherapy.

We successfully identified immunogenic epitopes derived from LSTVs based on FL-RNC seq. Our study indicated that LSTVs could largely expand the usable repertoire of MHC-I– and MHC-II–restricted epitopes in cancer. Notably, mRNA vaccines incorporating these neoepitopes demonstrated notable tumor inhibition either in monotherapy or in combination with PD-1 blockade. Our study reveals the therapeutic potential of mRNA vaccine targeting LSTV-derived neoepitopes. Moreover, mRNA vaccine therapy elicited long-term memory response and provided strong immunologic protection from tumor relapse after rechallenge. scRNA-seq analysis revealed that mRNA vaccine therapy not only elicited tumor-specific T cell responses but also remodeled the whole tumor microenvironment, including DC activation, neutrophil infiltration, as well as a shift of macrophage polarization from protumor M2-like to antitumor M1 phenotype. Collectively, these findings indicate that LSTVs dictate useful antigens for immunotherapy and are similar to previous studies (*30*, *37*).

Recent work has established that MHC class II–restricted neoepitopes are pivotal for antitumor cellular immune response and of high clinical relevance in predicting therapeutic response to immune checkpoint inhibitors (*25*, *38*, *39*). However, accurate prediction of immunogenic MHC-II neoepitopes remains challenging. We improved our MHC-restricted epitope prediction algorithm FIONA (*16*) to FIONA2 with reformed algorithm structure, which could predict both MHC-I– and MHC-II–restricted epitopes including mouse, enabling more accurate neoepitope prediction by adopting a refined hierarchical MHC naming method and an innovative negative data generation algorithm that better reflects the natural process of proteins in antigen-presenting cells. Epitope validation verified the high accuracy of FIONA2 in identifying epitopes for CD4^+^ T cell response. By using a monoclonal antibody approach for CD4^+^ T cell depletion, our data further confirm the importance of MHC-II neoepitopes in shaping antitumor immunity elicited by cancer vaccine. However, the ratio of MHC-II to MHC-I epitopes that would be optimal for anti-immune response warrants further investigation.

To further understand the vaccine-induced T cell immune response, we integrated coupled transcriptome and V(D)J analysis at single-cell resolution to characterize the patterns of gene expression and clonal dynamics in TILs after vaccination. T.eff and T.ex cell populations predominantly respond to vaccine, with enrichment of T.eff cells within tumors and a notable decrease in T.ex cell population. As expected, the clonal dominance in TCR repertoire was shifted toward T.eff cells after LSTV-based vaccine treatment, which might be attributed to the following mechanisms: (i) Vaccine treatment triggered ICD, reducing tumor-imposed suppression on T cells and alleviating T cell exhaustion. (ii) Cancer cells undergoing ICD released danger-associated molecular patterns (DAMPs) and tumor-associated antigens and remodeled the tumor microenvironment (*40*), particularly enhanced antigen-presenting capacity of DCs, decreased T_reg_ cell levels, and shifted myeloid cell populations, creating a favorable environment for antigen-specific T cells to function. Together, our data indicated that T.eff and T.ex cells respond to cancer vaccine efficiently, which induced ICD in cancer cells and consequently activated the tumor immune microenvironment to boost antigen-specific T cell immune response.

Our study has certain limitations. One primary limitation lies in the relatively large sample volume required for FL-RNC sequencing. This requirement exceeds that of short-read sequencing, which could potentially curb the clinical applicability of our approach due to the scarcity and procurement challenges of substantial clinical samples. Second, although our FIONA2 prediction model was meticulously designed to ensure accuracy in MHC-antigen presentation, it still has limitations in predicting immunogenicity due to the limited and somewhat ambiguous nature of the training data sourced from the Immune Epitope Database (IEDB) database. Furthermore, our methodology demonstrates superiority in identifying neoepitopes over traditional methods, in terms of quantity, sequence length, and dissimilarity. However, the practical loading capacity of mRNA vaccines is often insufficient due to manufacturing limitations. Prospective advancements in this field could potentially address these challenges. Improvements in sequencing protocols and the application of contrastive learning for more effective data mining may provide avenues to circumvent these limitations, thereby enhancing our understanding and practical application of these discoveries.

In summary, based on FL-RNC seq and a proprietary MHC prediction algorithm, we identified MHC-I– and MHC-II–restricted neoepitopes from LSTVs and further validated their application as cancer vaccine targets. We present a comprehensive solution for developing therapeutic cancer vaccines based on mRNA technology. This solution encompasses a complete workflow from upstream detection methods, intermediate data analysis and downstream neoepitope prediction, to final vaccine preparation. This universal approach notably expands the number of potential targets by several folds, indicating availability to cancers with lower TMB. The neoepitopes identified from LSTVs have higher immunogenicity due to their distinctness from the protein sequences produced by the coding regions in the genome. Additionally, compared to classic point mutation neoepitopes with a length of around 25 amino acids, LSTVs have longer lengths, potentially covering more MHC subtypes. Owing to these advantages, we believe that our work paves the way for the development and feasibility of personalized cancer vaccines in the future and can be applied to clinical studies in more challenging and clinically urgently needed cancer types such as acute myeloid leukemia and glioblastoma.

## MATERIALS AND METHODS

### Study design

The objective of this study was to comprehensively define tumor-specific and potentially immunogenic neoantigens produced from LSTVs. The FL-RNC seq technology for the identification of LSTV neoantigens in cancers is presented as a new strategy to enrich neoepitopes for therapy. To accurately predict potentially immunogenic neoantigens from LSTVs, an original algorithm FIONA2 was self-developed and applied to curated FL-RNC seq dataset obtained from an MC38 xenograft tumor model. We further developed an mTMG-LNP vaccine encoding these neoepitopes and then evaluated its therapeutic efficacy using the established murine MC38 xenograft model. Linking our findings with clinical studies, we performed retrospective analysis of public datasets from patient samples. Notably, the sample size for these bulk RNA-seq and scRNA-seq datasets is dependent on the original study design. All experiments performed in this study had at least three replicates to demonstrate biological reproducibility and to ensure adequate statistical power for comparisons. The study was not blinded, and no statistical methods were used to predetermine the sample size. Details for in vivo experiments, number of cells used, duration, and statistical tests are described below, in the Supplementary Materials, and in the figure legends.

### Animals and cells

Female C57BL/6 (6 to 8 weeks old) were obtained from Vital River Laboratory Animal Technology Co. Ltd. (Beijing, China). All animal experiments were approved by the Ethics Committee of the Nanjing University of China (no. 2203005). Mice were housed in a room at 20° to 22°C with a 12-hour light/dark cycle and at 35 to 70% humidity. B16-F10 melanoma and MC38 murine colon cancer cell lines were obtained from the American Type Culture Collection (Manassas, USA). B16-F10 and MC38 cells were cultured in Dulbecco’s modified Eagle’s medium (DMEM) containing 10% fetal bovine serum (FBS), penicillin G sodium (100 U/ml), and streptomycin (100 μg/ml) at 37°C in a humidified environment with 5% CO_2_.

### FL-RNC seq and data processing

To perform FL-RNC seq, MC38 tumors were pretreated with cycloheximide (Acmec, Shanghai, China), followed by prechilled PBS buffer washes and the addition of 2 ml of cell lysis buffer [1% Triton X-100 in ribosome buffer (RB buffer): 20 mM Hepes-KOH (pH 7.4), 15 mM MgCl_2_, 200 mM KCl, cycloheximide (100 μg/ml), and 2 mM dithiothreitol]. After a 30-min ice bath, cell lysates were collected and transferred to prechilled 1.5-ml tubes. After that, cell debris was removed by centrifuging at 4°C for 10 min. Supernatants were transferred to the surface of sucrose buffer (30% sucrose in ribosome buffer) and then ultracentrifuged at 4°C by T-865 fixed angle rotor (Thermo Fisher Scientific, Germany) to pellet RNCs. Isolation of RNC-RNA was carried out by TRIzol Reagent (Vazyme, China) according to the manufacturer’s instructions. RNC-RNA was extracted using TRIzol Reagent (Vazyme, China) per the manufacturer’s protocol. PolyA^+^ mRNA from RNC-RNA was isolated using VAHTS mRNA Capture Beads 2.0 (Vazyme, China). The cDNA library was prepared with VAHTS Universal V8 RNA-seq Library Prep Kit for Illumina (Vazyme, China) and sequenced on the Illumina NovaSeq 6000 at Chi Biotech Co. Ltd.

### Model building for MHC-presented and T cell–recognized neoepitope prediction

MHC multimer analysis is a widely used approach for detecting an antigen-specific T cell response (*41*). We focused on four key parameters related with neoepitope presentation and recognition: peptide length, amino acid residues at the interface, hydrophobicity, and MHC binding rank (*42*). We performed feature engineering to scale every feature to the same range considering the biologic value. First, the peptides coming from 9–amino acid peptides were padded to account for peptides of different lengths. Our study uses a prevalent padding approach, adding to the front of 6-mer peptides for alignment with 7-mer hotspots (*18*). The peptide sequences at contact positions were encoded from a protein transformer–based PLM, prot_t5_xl_uniref50 (https://huggingface.co/Rostlab/prot_t5_xl_uniref50). This model is based on T5 and was pretrained on a large corpus of protein sentences.

The prediction model FIONA2 (fiona.therarna.cn) combined a 1D CNN model and an MLP layer involving NetMHCpan and NetMHCIIpan rank and hydrophobicity. The 1D CNN had kernel sizes of 1, 3, 5, and 7, with each max pooled and concatenated to a layer. In parallel, −log transformed rank and hydrophobicity metrics (i.e., the proportion of A, V, L, M, and W) have been added as an MLP layer (*20*). 1D CNN and MLP layers were concatenated and merged into a dense layer of 256 units for classification. The hyperparameters of the final 1D CNN models were fine-tuned via grid search. The parametric model’s final settings are as follows: learning rate = 1 × 10^−5^, weight decay = 1 × 10^−6^, dropout rate = 0.1, batch size = 50, dense layer node = 2000, and dense layer node = 256, giving ROC-AUC of 0.764 by 10-fold cross-validation. The final hyperparameters for self-antigen model are as follows: learning rate = 0.001, weight decay = 0.01, dropout rate = 0.2, batch size = 100, MLP dense = 1500, and dense layer node = 512. All deep learning architectures are implemented using Python TensorFlow v2.8.0 package. Peptides with high-confidence prediction will be selected for MHC target test.

Training data and testing data were gathered from IEDB (www.iedb.org). The ROC and PR curve (Fig. 2, B and C) yielded an ROC-AUC of 0.98 and a PR-AUC of 0.99, as determined by 10-fold cross-validation.

### Immunization

mRNA-LNPs, encoding either the LSTV-derived neoepitopes or irrelevant RNA tubulin, were prepared for mouse immunization. The mRNA-LNP solution containing encapsulated mRNA (0.1 mg/ml) was stored at 4°C. Mice were immunized by subcutaneous injection of a dose of 20 μg of mRNA-LNPs near the inguinal lymph nodes at the indicated time. The tumor volume was monitored every 2 to 3 days after immunization and calculated using an ellipsoid formula (π/6 × height × length × width).

In the prophylactic model, the splenocytes were collected on day 50 to analyze specific tumor killing. Then, the splenocytes were cocultured with MC38 cells and B16-F10 cells at a ratio of 10:1 for 24 hours. Nonadherent cells were removed, and adherent cells were washed with PBS. The CCK8 assay was carried out to evaluate the percentage of specific lysis.

For CD4^+^ and CD8^+^ T cell depletion in the MC38 mouse model, mice received either αCD8 (100 μg/mice) or αCD4 (250 μg/mice) or αIgG (100 μg/mice) antibodies 2 days before tumor inoculation, on the day of tumor inoculation, and every 4 days after tumor inoculation for the duration of the experiment. The blood samples were collected and centrifuged at 1500*g* at 4°C for 10 min. CD8^+^ T and CD4^+^ T cell depletion efficiencies were then validated by flow cytometry.

In the αPD-1 combination model, the mRNA-LNP vaccine was administered five doses (20 μg, s.c.) on day 4, 7, 10, 13 and 17 post-tumor inoculation. The additional procedure was that αPD-1 or αIgG (5 mg/kg) was intraperitoneally injected into the mice on days 3, 6, and 9.

### Flow cytometry analysis

Single-cell suspensions from either spleen, blood, or tumor were incubated with TruStain FcX (clone 93) for 15 min to block nonspecific binding before staining with the conjugated antibodies or tetramer. 7-AAD Viability Staining Solution or Fixable Viability Zombie Violet was used to exclude dead cells. IFN-γ, TNF-a, Foxp3, and CD206 were stained intracellularly by using Cytofix/Cytoperm W/Golgi Stop Kit (BD) following the manufacturer’s instructions. All staining steps were conducted at 4°C in the dark. Data were collected on CytoFLEX flow cytometer (Beckman Coulter Inc.) and analyzed by FlowJo (Tree Star Inc., Ashland, OR) software. Antibodies used were listed in table S6.

### ELISpot assays

Mouse IFN-γ ELISpot assays were performed with a Mouse IFN-γ Precoated ELISpot Kit according to the manufacturer’s instructions (Dakewe). A total of 1 × 10^5^ splenocytes per well were restimulated ex vivo with single peptides (5 μg/ml for each), negative control [dimethyl sulfoxide (DMSO)], or positive control [phorbol 12-myristate 13-acetate (PMA) (50 ng/ml) + ionomycin (1 μg/ml)]. Twenty-four hours after restimulation, biotinylated antibody and streptavidin–horseradish peroxidase (HRP) were added successively after cell lysis. Then, AEC peroxidase substrate was added, and spots were counted using an ELISpot plate reader (Mabtech IRIS Fluorospot Reader, Mabtech). Spot numbers were evaluated using Mabtech Apex software v.1.1.52.121.

### Single-cell suspension preparation and sorting

MC38 tumors were harvested on day 21 after inoculation. Tumor tissues were cut into approximately 1-mm^3^ pieces in the RPMI 1640 medium (Invitrogen) with 2% FBS and enzymatically digested with MACS Tumor Dissociation Kit (Miltenyi Biotec) for 40 min on gentleMACS Dissociator according to the manufacturer’s instruction. The dissociated cells were subsequently filtered through a 70-μm cell strainer, centrifuged at 500*g* for 10 min, and resuspended in MACS buffer (1× PBS with 2 mM EDTA and 0.5% bovine serum albumin) for counting. CD45^+^ cells were isolated by flow cytometry, washed, and resuspended in FACS buffer (1× PBS with 2% FBS).

### Unsupervised clustering analysis of scRNA-seq dataset

The Seurat pipeline was applied to the combined expression data (*26*). Principal components analysis (PCA) was performed on the scaled data of all 16,092 genes. Then, a UMAP dimensional reduction was performed based on the first 35 PCA components to obtain a 2D projection of the cell states. Clusters were identified using shared nearest neighbor (SNN)–based clustering based on the same principal components. We used the function Find Clusters for clustering with resolution from 0.1 to 1.5, leading to 6 to 32 clusters. For each resolution, the silhouette values were calculated. Such value was used to determine the optional cluster number *k*. We performed the first round of clustering with low resolution 0.1 and annotated each cluster by known markers. We identified eight major cell types, including seven immune cell types and one nonimmune cell type. After removing the nonimmune cell population, we selected the higher resolution of 1 by maximizing the average silhouette coefficient, which led to a total number of 23 clusters. We also excluded two additional clusters of cells because they showed little immune cell signature gene expression, indicating that they may be a mixed stromal cell subset (epithelial cells, endothelial cells, fibroblasts, and malignant cells). After a series of filtering described above, 21 clusters corresponding to 16,945 cells were retained for downstream analyses (tables S4 and S5).

### Statistical analysis

Data are presented as indicated in the figure legends as means ± SEM and analyzed by Student’s *t* test or analysis of variance (ANOVA) with Holm-Šídák test for multiple comparisons correction. Survival analyses were conducted with the use of the Kaplan-Meier and Cox proportional hazards regression methods. Survival distributions were compared across groups with the use of the log-rank test. GraphPad Prism 9 software was used for all statistical analysis, and all *P* values from multiple tests were corrected by Benjamini-Hochberg method using the p.adjust function. Differences were considered significant at *P < 0.05, **P < 0.01, ***P < 0.001, and ****P < 0.0001.

## Supplementary Materials

### The PDF file includes:

Supplementary Methods Figs. S1 to S18 Tables S1 to S7 Legends for data S1 to S5 References
