# Supplementary material for: Large-scale transcript variants dictate neoepitopes for cancer immunotherapy
Source: Sci Adv. 2025 Jan 31;11(5):eado5600. doi: 10.1126/sciadv.ado5600 (PMC11784853; doi:10.1126/sciadv.ado5600)
Supplement: Supplementary file 1 — Supplementary Methods Figs. S1 to S18 Tables S1 to S7 Legends for data S1 to S5 References [file sciadv.ado5600_sm.pdf]

Science Advances
AAAS

Supplementary Materials for

Large-scale transcript variants dictate neoepitopes for cancer immunotherapy

Shiliang Ji *et al.*

Corresponding author: Zhi Ding, dingzhi@nju.edu.cn; Zhen Huang, zhenhuang@nju.edu.cn; Junfeng Zhang, jfzhang@nju.edu.cn

Sci. Adv. **11**, eado5600 (2025)
DOI: 10.1126/sciadv.ado5600

The PDF file includes:

Supplementary Methods
Figs. S1 to S18
Tables S1 to S7
Legends for data S1 to S5
References

Other Supplementary Material for this manuscript includes the following:

Data S1 to S5

Supplementary materials contain Supplementary methods, Supplementary Figures and Supplementary Tables

Supplementary methods

RNA synthesis

The coding sequences (CDS) harboring the predicted neoepitopes were selected as an antigen in this work. The associated amino acid sequences are provided herein (fig. S2). N1-methyl pseudouridine-modified mRNAs corresponding to these sequences were generated in vitro through a conventional T7 RNA polymerase-mediated transcription reaction, capped, and subsequently purified via fast protein liquid chromatography. RNA integrity was assessed by microfluidic capillary electrophoresis (Agilent Fragment Analyzer), and the concentration, pH, osmolality, endotoxin level and bioburden of the solution were determined.

mRNA- LNP preparation and characterization

mRNA-LNPs were prepared by mixing mRNA in an aqueous phase with lipid mixtures in an ethanol phase using a T-junction mixing device as reported previously (*43*). In brief, mRNA was dissolved in citrate buffer (100 mM, pH 4.0). The lipid mixtures, consisting of ionizable lipid, 1,2-distearoyl-sn-glycero-3-phosphocholine (DSPC), cholesterol, and PEG-lipid, were dissolved in anhydrous ethanol at a molar ratio of 46.3:9.4:42.7:1.6. The N/P ratio was maintained at 6:1. Subsequently, the ethanol and aqueous phases were mixed in the T-junction device at a volume ratio of 3:1. Then mRNA-LNPs underwent dialysis against a buffer at pH 7.4 for 18 h, followed by sterilization through a 0.22-μm filter and storage at 4 °C for future use. The NS300 (Malvern Panalytical) was employed to measure the average diameter, polydispersity index (PDI), and zeta potential. The concentration of leaked mRNA (Cleak) was determined using a RiboGreen (Thermo Fisher, R11490) following the manufacturer's protocols. Additionally, mRNA-LNPs were lysed with 2% Triton X-100 to determine the concentration of total mRNA (Ctotal). The encapsulation efficiency (EE) was calculated using the following equation: Encapsulation efficiency(EE)=(Ctotal-Cleak) /Ctotal ×100%.

Enzyme-linked immunosorbent assay (ELISA)

Ninety-six-well ELISA microplates (Greiner) were coated with 2 ng/μL different primary antibody in coating buffer (Dakewe) at 4 °C for 15 h. After washing and blocking, serially diluted mouse sera were incubated in plates at 4 °C for 2 h. Then the secondary antibody, goat anti-mouse IgG H&L-conjugated HRP (Abcam), was incubated in plates at room temperature for 1 h. 3′5′-tetramethylbenzidine (TMB) (Dakewe) was used as the substrate to detect antibody responses. Details of the ELISA kits are provided in Table.S5. Data were collected using a microplate reader (Molecular Devices) and SoftMax Pro software version 7.1.0.

Preparation of scRNA-seq libraries and sequencing

Single-cell RNA-seq was performed by the 10x Genomic single cell 5' VDJ library platform. FACS-sorted cells were adjusted to 900-1100 cells/μL in suspension. Cells were loaded between 10,000 and 17,000 cells/chip position using the Chromium Single cell 5' VDJ Library, Gel Bead & Multiplex Kit and Chip Kit (10x Genomics, V1 barcoding chemistry). Single-cell gene expression profiling and TCR libraries were generated according to the manufacturer's instructions. Purified libraries were analyzed by Nextseq sequencer with 150-bp paired-end reads at a targeted median read depth of 50,000 reads per cell from total gene expression libraries and 5,000 reads per cell for TCR libraries (cycle specifications150:8:0:150 [R1: i7: i5: R2]).

Data processing of single-cell RNA-seq and scTCR-seq libraries

The Cell Ranger (v2.1.1) Software Suite was used to process the raw sequence data into gene expression profiles. Fastq files were aligned to the mm10 reference genome, filtered, and quantified using Cell Ranger count, with the 'recovered-cells' parameter set to 10,000 per library. The output files of Cell Ranger for each sample included feature barcode and gene expression (in UMI count). Gene expression data were obtained using the cellranger aggr, which performed an inter-sample normalization and merged the results of all samples into one file, to remove the potential technique variability of samples.

For scTCR-seq data, TCR reads were aligned to the mm10 reference genome and TCR annotation was performed using the 10x cellranger VDJ pipeline. Approximately

87% of T cells in scRNA-seq data were assigned a TCR, with over 80% having at least one full-length productive CDR3 for both TRA and TRB. The clonotype of each T cell was represented by the paired alpha and beta CDR3 sequences, and clone sizes ranged from 49 cell to 622 cells.

Pre-processing and quality control

The remaining count table of cells was log-normalized and implemented in the R package Cell Ranger™ R Kit. A total of 16,092 protein-coding genes and 16,945 cells were retained in the final expression table. Post-alignment counts were processed with the R package Seurat. Low-quality cells were filtered out based on UMI count criteria: either fewer than 200 UMIs or genes expressed in less than three cells. CD45 expression was defined as the average UMI counts of Ptprc, and those cells with Ptprc expression above zero were retained for further analysis. We identified the status of each cluster for each cell based on the gene expression data. A small subset of non-immune ($CD45^-$) cells, likely false positives from flow sorting, were excluded.

Identification of Cluster-Specific Genes

We used the FindAllmakers and FindMarkers functions with the Wilcox ran-sum test to identify the marker genes. For each cluster, only the genes that were expressed by more than 30% of total cells with p-value < 0.05 (Benjamin-Hochberg method) were considered (data S1). The function of each cluster was annotated and manually confirmed based on the expression of well-known cell-type-specific marker genes.

Characterization of cell cluster enrichment and TCR clonotypes

To characterize the tissue distribution of meta-clusters, odds ratios (OR) were calculated and used to indicate preferences (*44*). Specifically, for each meta-cluster i and tissue j, a 2 x 2 table included cell counts of meta-cluster i in tissue j, in other tissues, and counts of other meta-clusters in tissue j and other tissues. Then Fisher's exact test was applied on this contingency table to obtain OR value and corresponding p-value. We found that all ORs > 1.5 or ORs < 0.5 had adjusted p-values < 1e-10. Hence, a higher OR with a value > 1.5 indicated a higher preference of meta-cluster i in tissue j, while a lower OR with a value < 0.5 indicated that meta-cluster i was preferred not to distribute in tissue j.

We integrated the sequencing data of both scRNA and scTCR, if at least two cells shared an identical productive Alpha-Beta pair, those Alpha-Beta pairs were defined as clonal TCRs. Clonal T cells were defined as T cells with clonal TCR. Finally detected 616 TCR groups, corresponding to 1,770 different sorted T cells (data S3).

We defined the Clonal score (CS) to quantify the degree of clonal expansion for T cell clusters. The CS is calculated on the well-described TCR clonality measurement, which uses normalized Shannon entropy (*45*). The clonotype is determined by identical paired Alpha and Beta TCR chains. A higher CS (ranging from 0 to 1) indicates higher clonal expansion. T cells that have TCRs in the same group are expected to recognize the same antigen. We only retained the TCR groups that corresponded to more than 20 T cells for further analysis (data S4).

For T cells with identical TCR clonotypes, they often presented in different clusters. Thus, they represented a potential transition from one phenotypic state to another through clonal expansion. In this work, we used TS method (*46*) to characterize the transition potential between two specific clusters.

Pseudotime trajectory inference

To determine the potential lineage differentiation between those T cell populations with high TCR sharing, we studied the transcriptional and developmental trajectories of different $CD8^+$ T cell populations using Monocle v.2.0 (*47*). Raw count data and clustering annotations are used as input to Monocle. Monocle skews gene expression dynamics by using a reverse graph embedding method and each cell was placed on the inferred pseudotime trajectory. Functional ‘states’ of trajectories are identified based on the assumption that trajectories have a tree-like structure based on the tree structure of segments.

Gene set enrichment analysis

The R packages fgesa and msigdbr were used to perform the gene set enrichment analysis. The pathway gene sets we used were extracted from online databases KEGG, BIOCARTA and curated Hallmark gene sets (*48*). For a given cluster, KEGG was performed using each pathway gene set against ranked gene lists from comparison with other clusters. Pathways were considered as enriched if the BH adjusted fdr < 0.1, and

the normalized enrichment scores (NES) were calculated to represent the enrich level (data S5).

Supplementary Figures

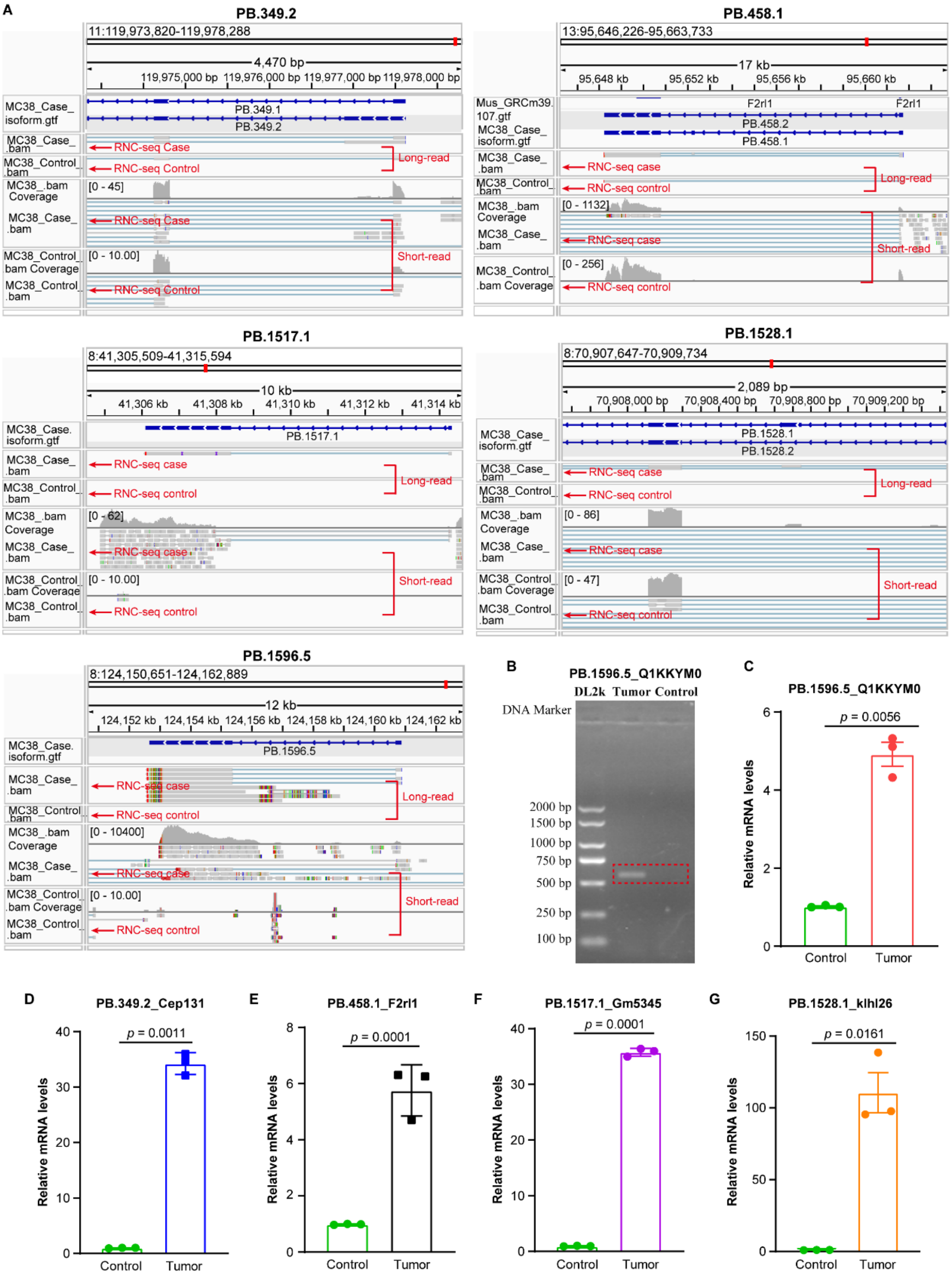

Fig. S1. Detection and validation of large-scale transcriptional variants in tumor tissue of MC38 bearing mice. Mice were grafted with 1 x 10^6 MC38 tumor cells and then tumors were harvested when the volume reached approximated 1200 mm^3. RNC-mRNA complexes from MC38 xenografts and C57BL/6J mouse normal colon tissue were sequenced in long-read PacBio HiFi and

Short-read Illumina. **(A)** Subsequent transcript isoform analysis using paired normal colon tissue revealed five tumor-specific LSTVs: PB.1596.5 (Q1KKYM0), PB.349.2 (Cep131), PB.458.1 (F2rl1), PB.1517.1 (Gm5345) and PB.1528.1 (Klhl26). **(B)** Representative gel electrophoresis plots show the expression of the longest tumor-specific transcript PB.1596.5 in the MC38 subcutaneous tumor and control samples. **(C** to **G)** The expression levels of five tumor-specific LSTVs in MC38 subcutaneous tumor and normal colon tissues were verified by qPCR. Data represents mean ± SEM (n = 3).

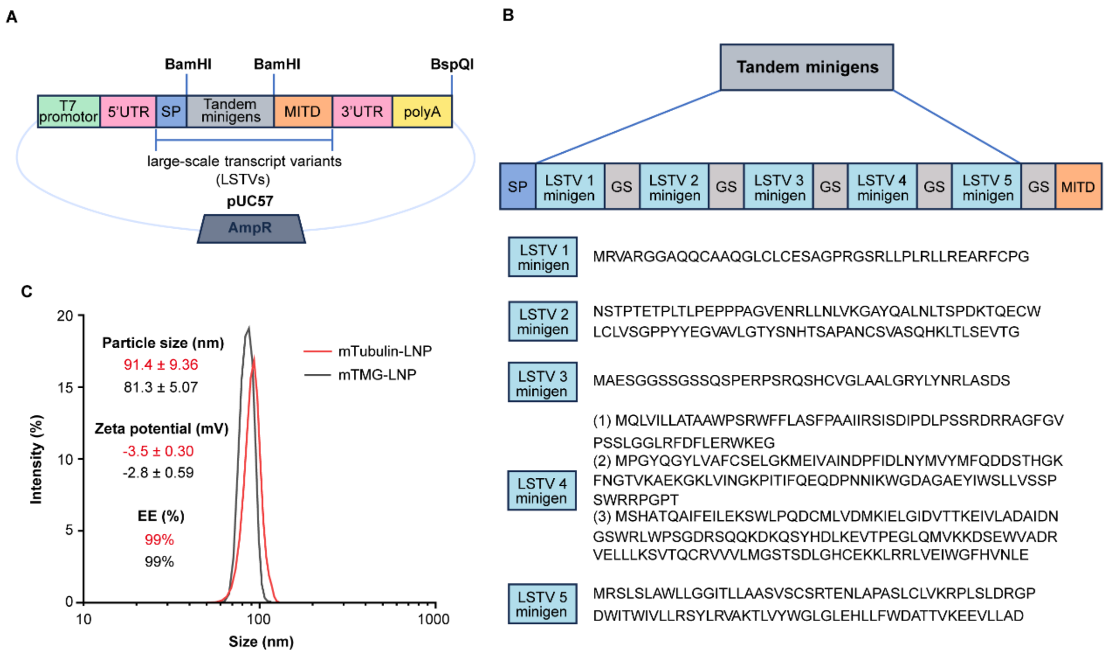

Fig. S2. Vaccine design, synthesis and characterization of mRNA-LNP. According to the Self-developed epitope prediction model FIONA2, the neoepitope coding sequences (CDS) with the best coverage and immunogenicity were selected as mRNA vaccine sequences. **(A)** Schematic representation of concatemer vaccine. Locations of restriction enzyme recognition sites are indicated. **(B)** Design of the tandem of minigenes and Amino acid sequences of five tumor-specific LSTVs containing MHC-I/II-restricted model epitopes (amino acid sequences were screened by the epitope prediction model). **(C)** mRNA-LNP was prepared by microfluidic system. Particle size was measured by DLS in PBS. Zeta potential was measured by electrophoretic Light Scattering in PBS. EE: Encapsulation efficiency. Data represents mean ± SEM (n = 3). GS, glycine serine; AmpR, ampicillin resistance gene; ORFs, open reading frames; UTR, untranslated region; SP, signal peptide; MITD, MHC class I trafficking domain. LSTV1: PB.349.2_ORF1; LSTV2: PB.1596.5_ORF5; LSTV3: PB.1528.1_ORF4; LSTV4: PB.1517.1_ORF11/12/21; LSTV5: PB.458.1_ORF7.

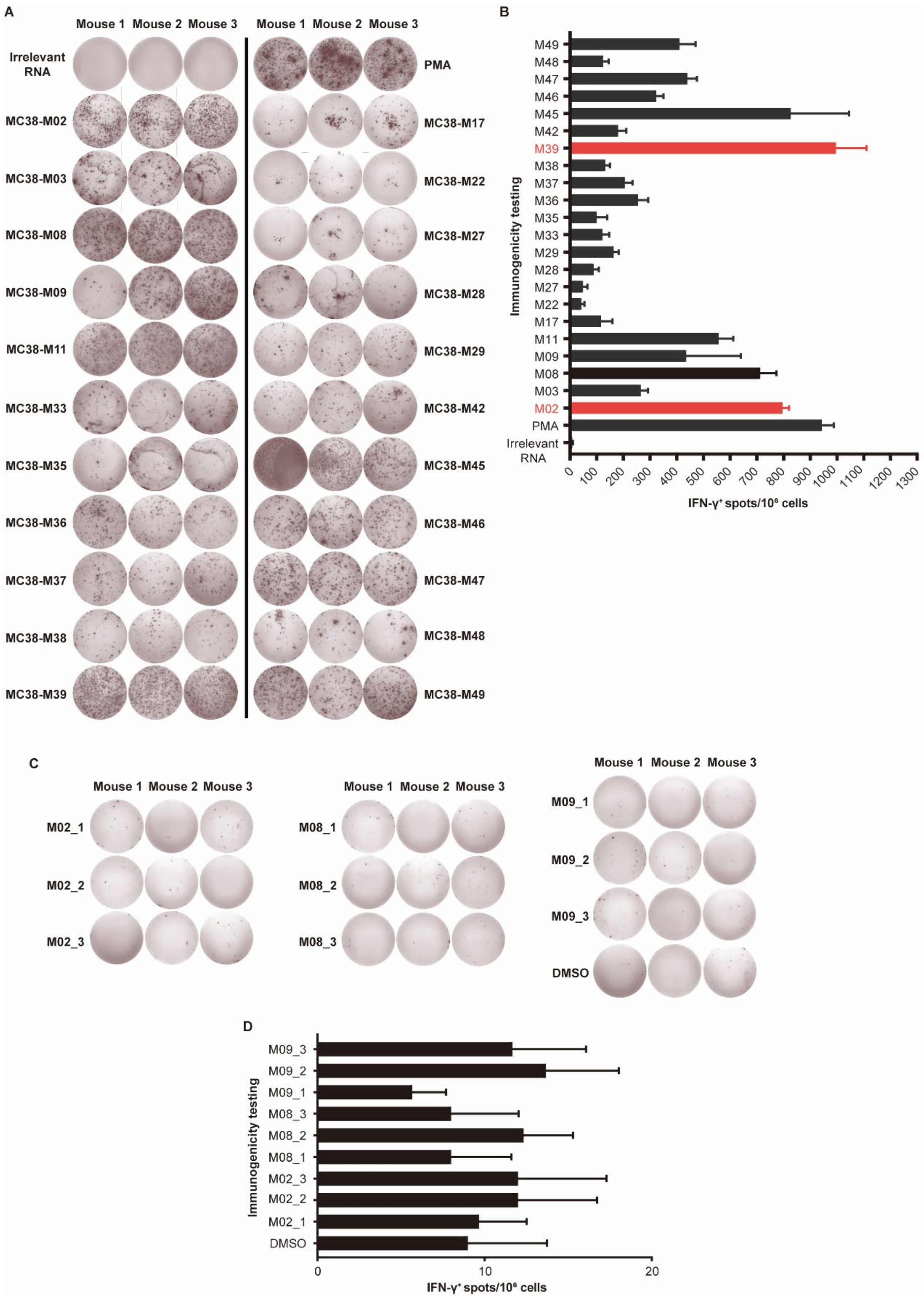

Fig. S3. Immunogenicity of predicted epitopes identified by ELISPOT in MC38 subcutaneous tumors. Naïve C57BL/6J mice were immunized with five doses of neoepitope vaccine (20 μg/ dose) of mTMG-LNP or irrelevant RNA vaccine (Control). Spleen cells from immunized mice were collected and incubated with each of the predicted potential epitopes, or PMA (positive control) or

DMSO. **(A)** Representative spot plots show the frequencies of Interferon (IFN)-γ-producing CD8$^+$ T or CD4$^+$ T cells from spleen cells. **(B)** Statistics of positive spot plots of IFN-γ secretion. MC38-M02 and MC38-M39 are representative MHC-I-restricted epitopes and MHC-II-restricted epitopes, respectively (red marked). **(C-D)** To verify whether the neoantigen vaccine produces any immune response against the self-antigens that may be encoded in its longer antigens, mice were administered as described above. Representative self-antigens source similar peptides elicit any immune response detected by ELISPOT assay (C) and statistics of positive spot plots of IFN-γ secretion (D). Data represents mean ± SEM (n = 3 mice).

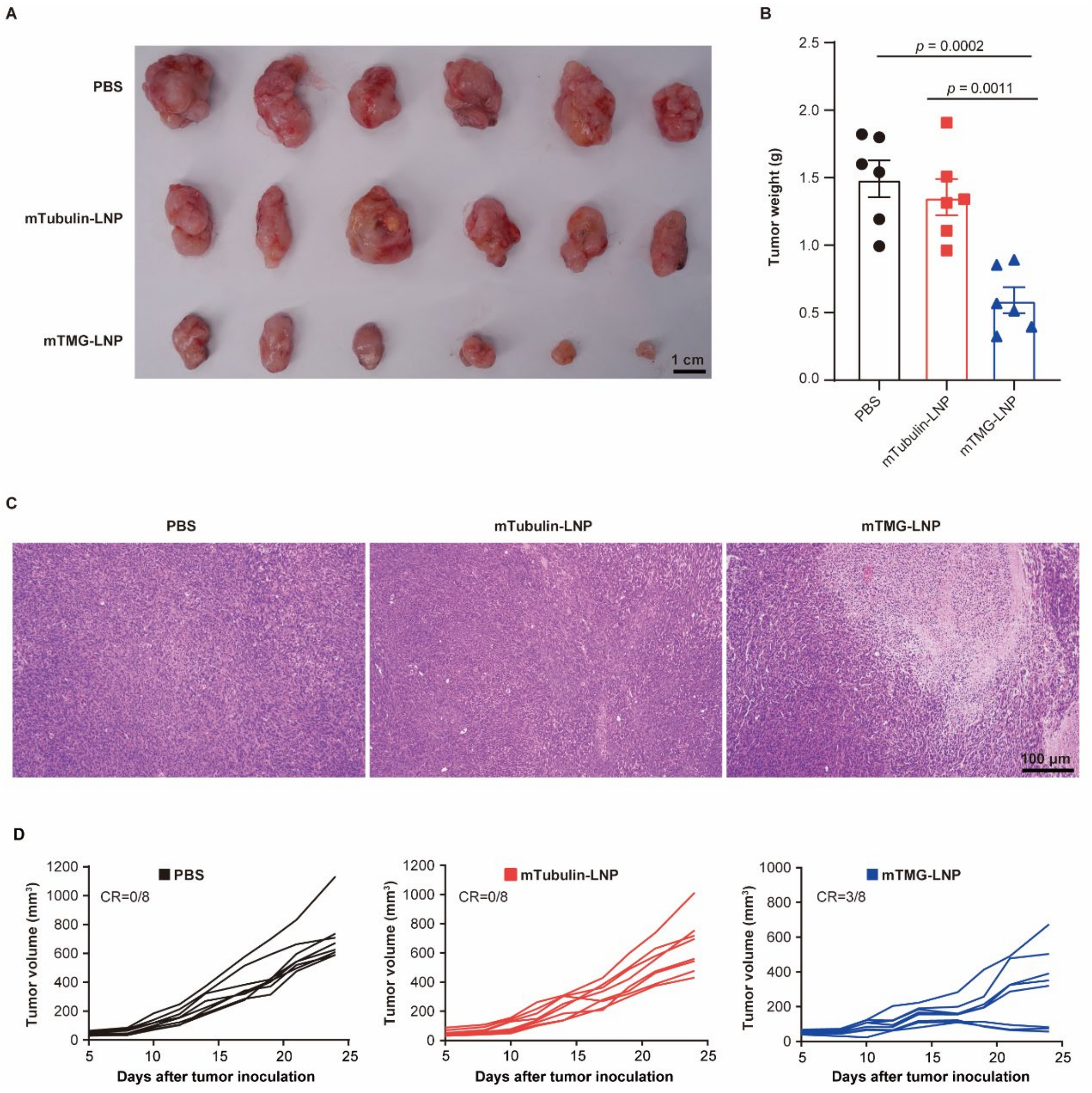

Fig. S4. Antitumor effects of neoantigen vaccines in an MC38 xenograft model. Mice were grafted with 1 x 10^6 MC38 tumor cells and s.c. immunized with five doses of neoepitope vaccine (20 μg/dose) of mTMG-LNP. Control mice were vaccinated with 20 μg of irrelevant RNA vaccine (mTubulin-LNP). **(A)** Representative images show isolated tumors from vaccinated mice on day 21. **(B)** Tumor weight was measured (n = 6 mice). **(C)** H&E staining of tumor tissue at the end of the experiment in the subcutaneous MC38 tumor model. **(D)** Individual tumor growth curves of mice in the indicated groups (n = 8 mice). The data are presented as the mean ± SEM and were analysed by one-way two-sided ANOVA with GraphPad Prism software.

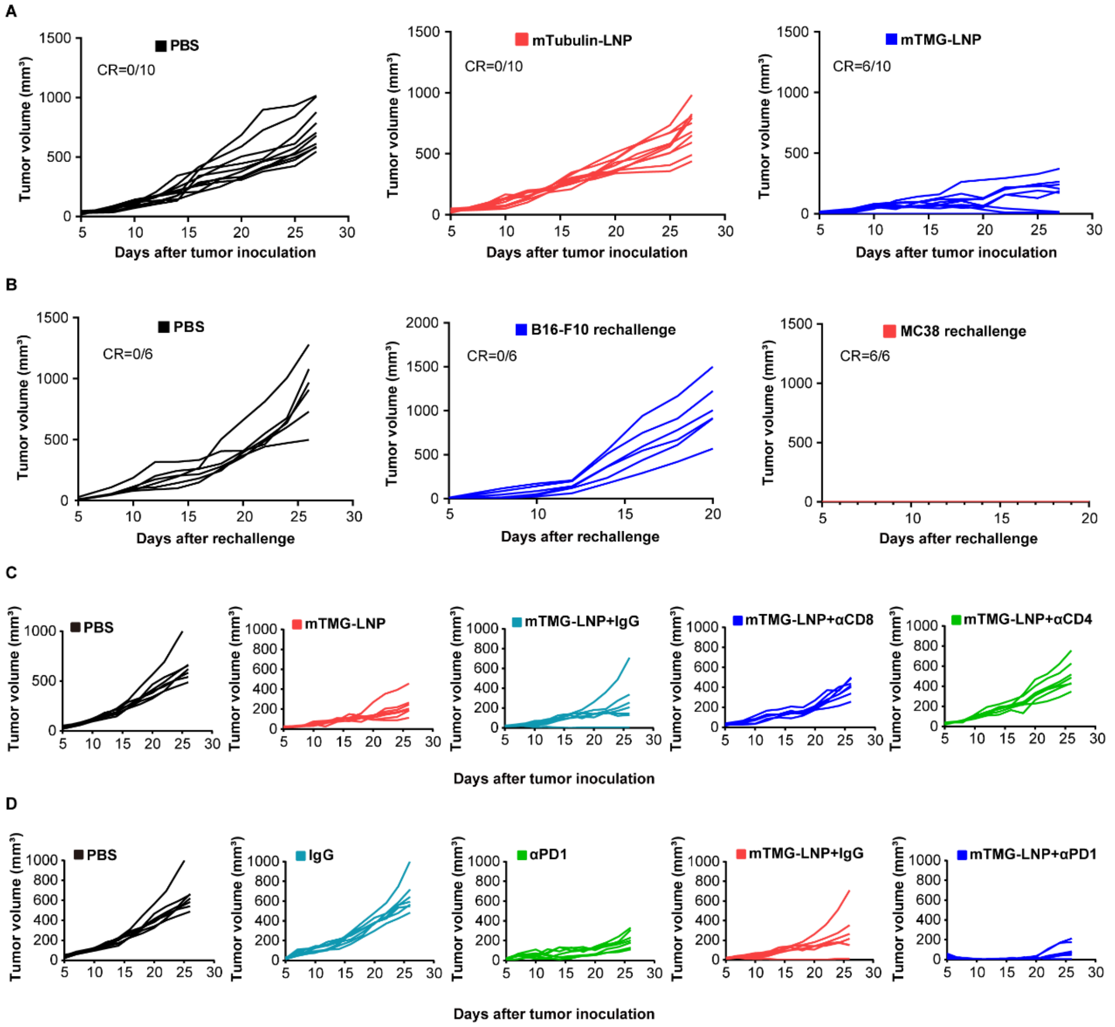

Fig. S5. Tumor growth curves of individual mice in therapeutic and preventative studies of neoantigen vaccine in colorectal cancer and melanoma. (A) Tumor growth curves of individual mice in preventative studies of mTMG-LNP vaccine in colorectal cancer (n = 10 mice). **(B)** Tumor growth curves of individual mice in rechallenge studies of mTMG-LNP vaccine in colorectal cancer. In the melanoma model group, MC38 tumor-bearing mice cured by mTMG-LNP vaccine were selected to be inoculated with 1.5 x 10^5 B16-F10 cells again. Naïve C57BL/6J mice were grafted with 1 x 10^6 MC38 tumor cells as control (n = 6 mice). **(C)** Tumor growth curves of individual colorectal cancer mice treated with mTMG-LNP vaccine while $CD4^+$ or $CD8^+$ cells were depleted (n = 6 mice). **(D)** Tumor growth curves of individual colorectal cancer mice treated synergistically with mTMG-LNP vaccine and aPD-1(n = 7 mice).

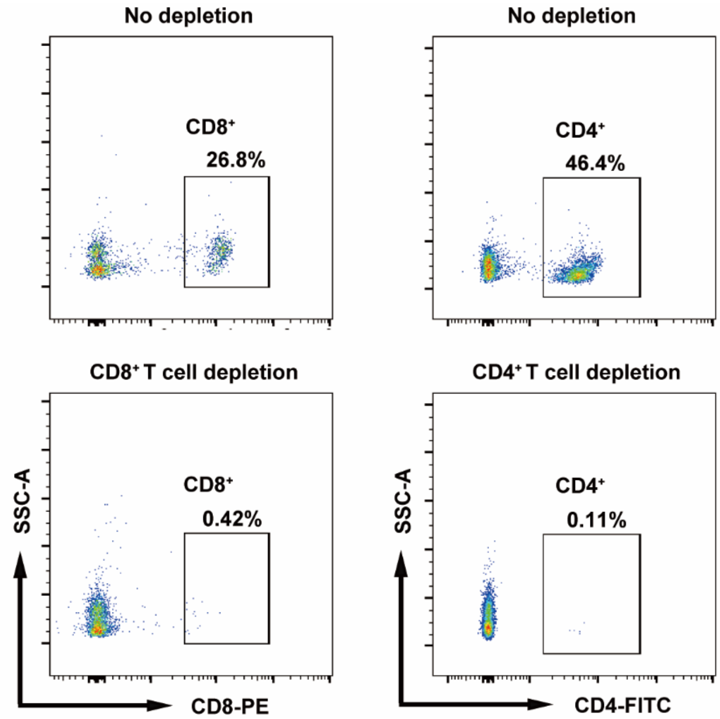

Fig. S6. Depletion of selected cell subsets during LNP-mTMG therapy. Cell subsets were depleted by administering depleting antibodies i.p. beginning two days prior to initiation of LNP-mTMG therapy, as detailed in Method. Depletion of $CD8^+$ T cells (CD8), or $CD4^+$ T cells (CD4) was confirmed by analyzing the peripheral blood of the mice by flow cytometry (n = 5 mice, biologically independent replicates).

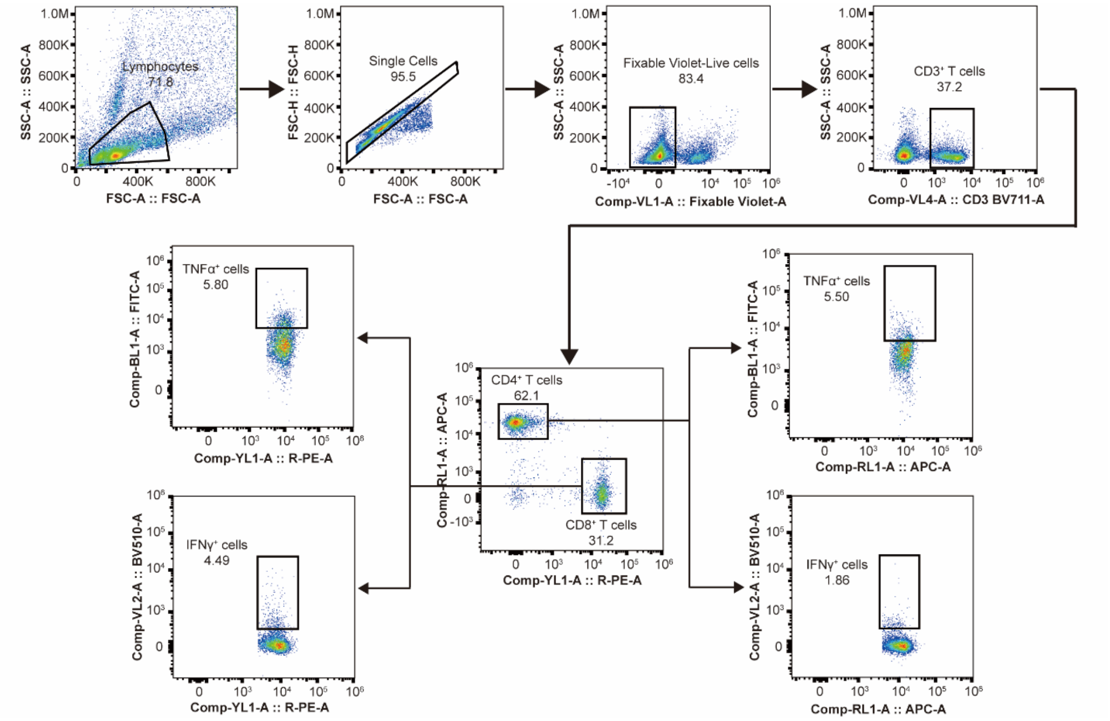

Fig. S7. Gating strategy for flow cytometric analysis of Intracellular cytokine in T lymphocytes in the spleen after treatment with neoantigen vaccine.

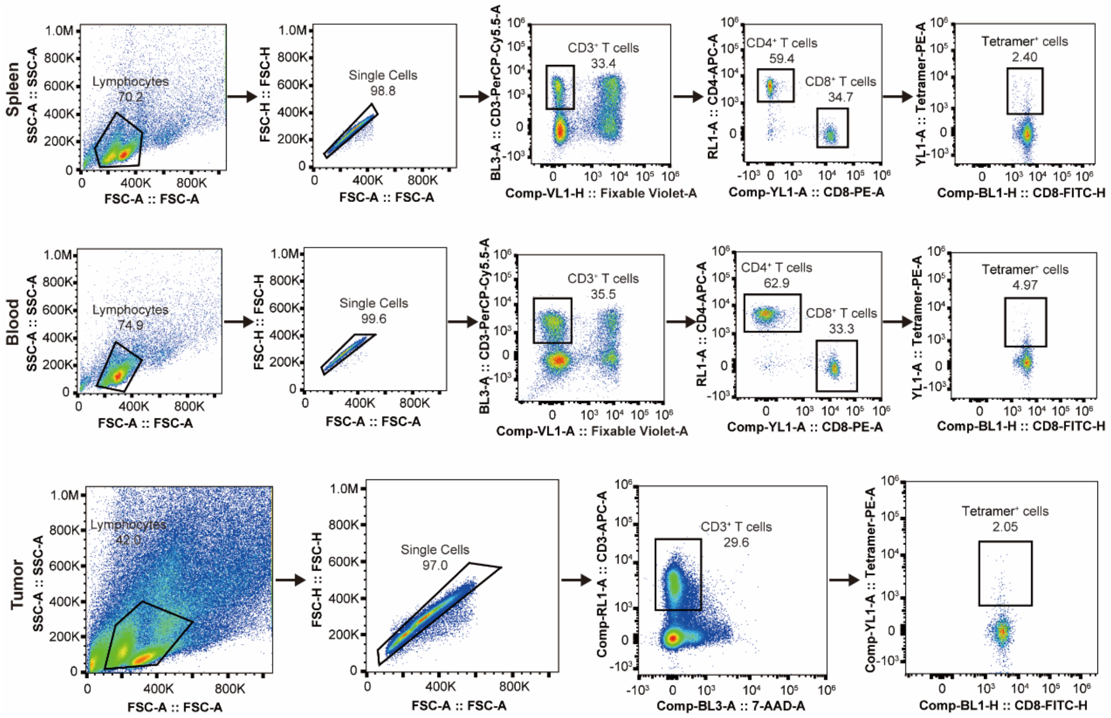

Fig. S8. Gating strategies for flow cytometric analysis of neoantigen-specific T cell (tetramer^{+}) in different organs after treatment with neoantigen vaccine.

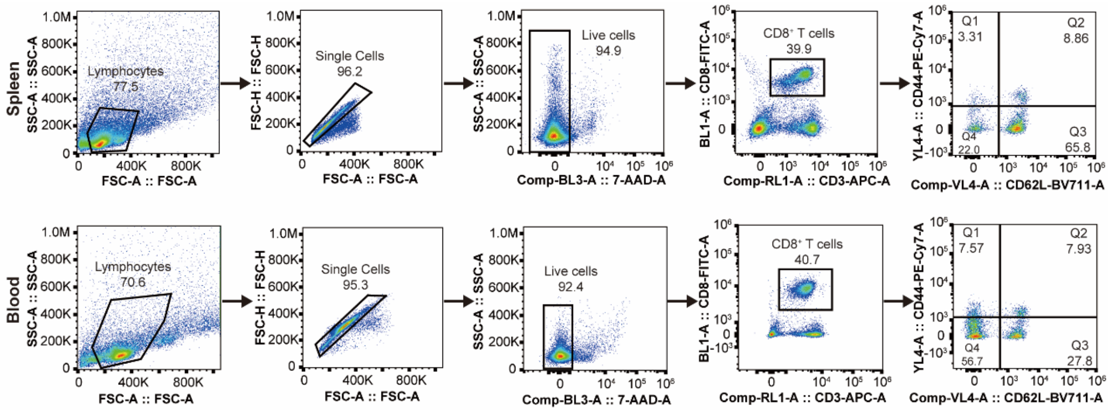

Fig. S9. Gating strategy for flow cytometric analysis of various memory T cells in different organs after treatment with neoantigen vaccine.

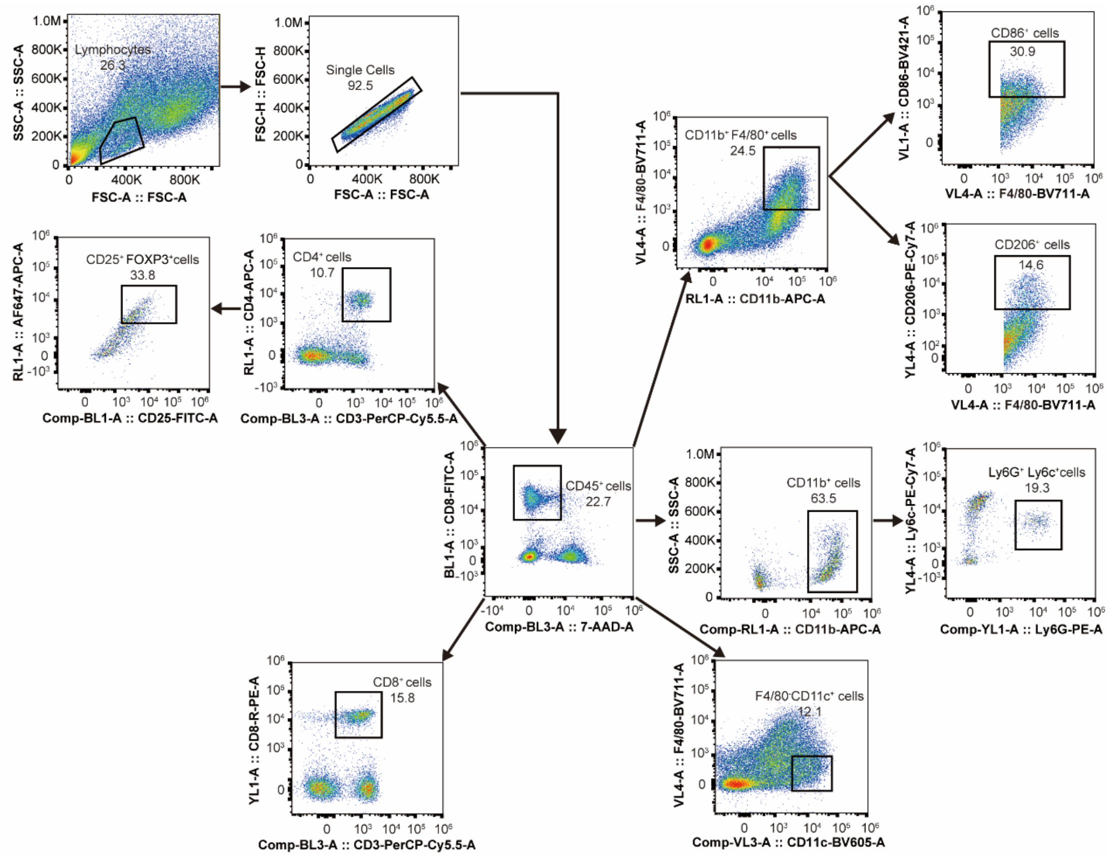

Fig. S10. Gating strategy for flow cytometric analysis of TME changes in tumor tissue after treatment with neoantigen vaccine.

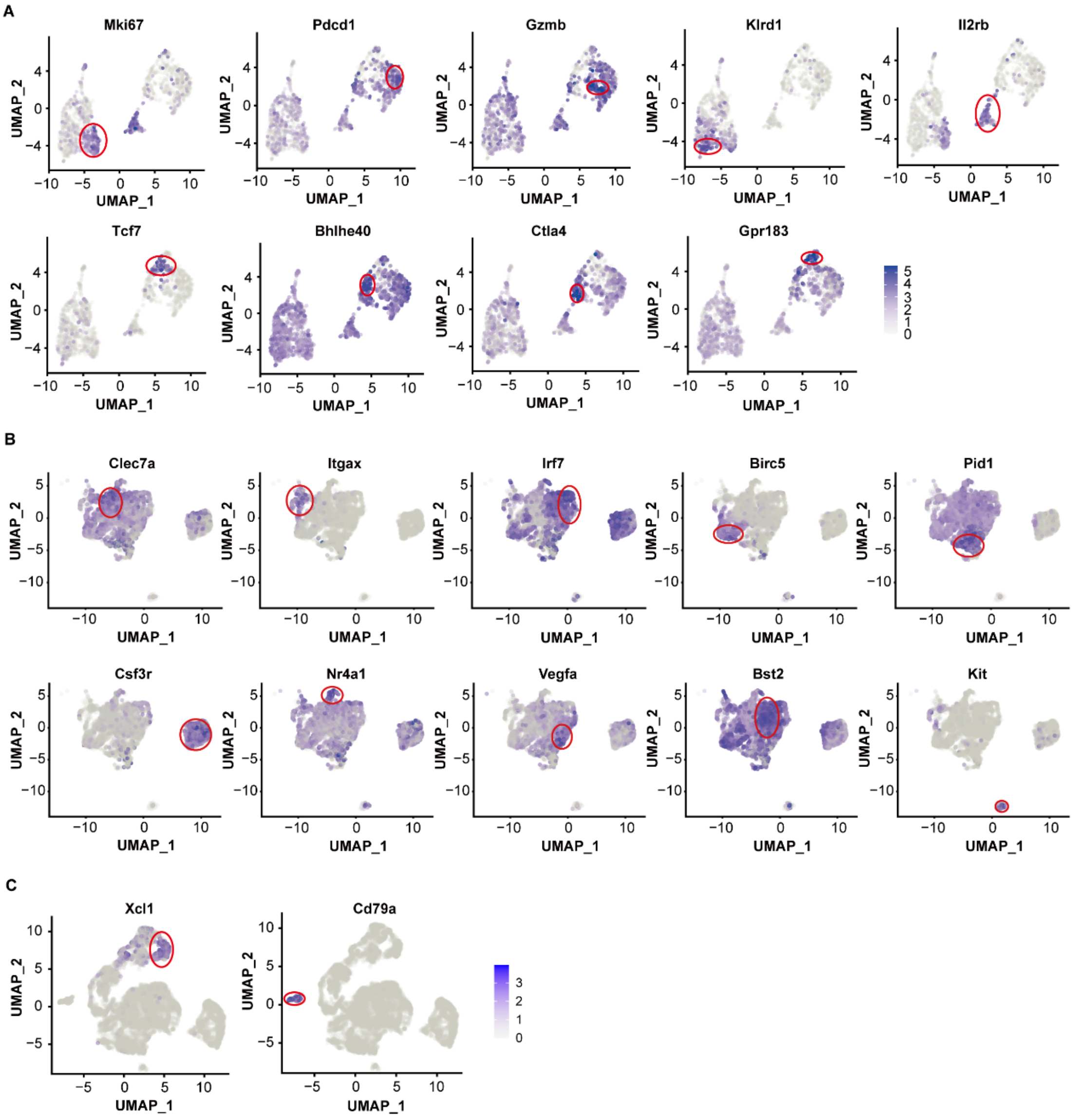

Fig. S11. Expression levels of signature genes in each cell cluster. UMAP plot of expression levels of selected genes in different clusters indicated by the red oval, corresponding to the 21 main clusters in Figure 4A. Each dot denotes an individual cell. **(A)** Representative 9 T cell clusters, including 6 CD8^{+} T cell clusters and 3 CD4^{+} T cell clusters. **(B)** Representative 10 myeloid cell clusters. **(C)** Another 2 smaller clusters of immune cells NK and B cells (n = 4 mice).

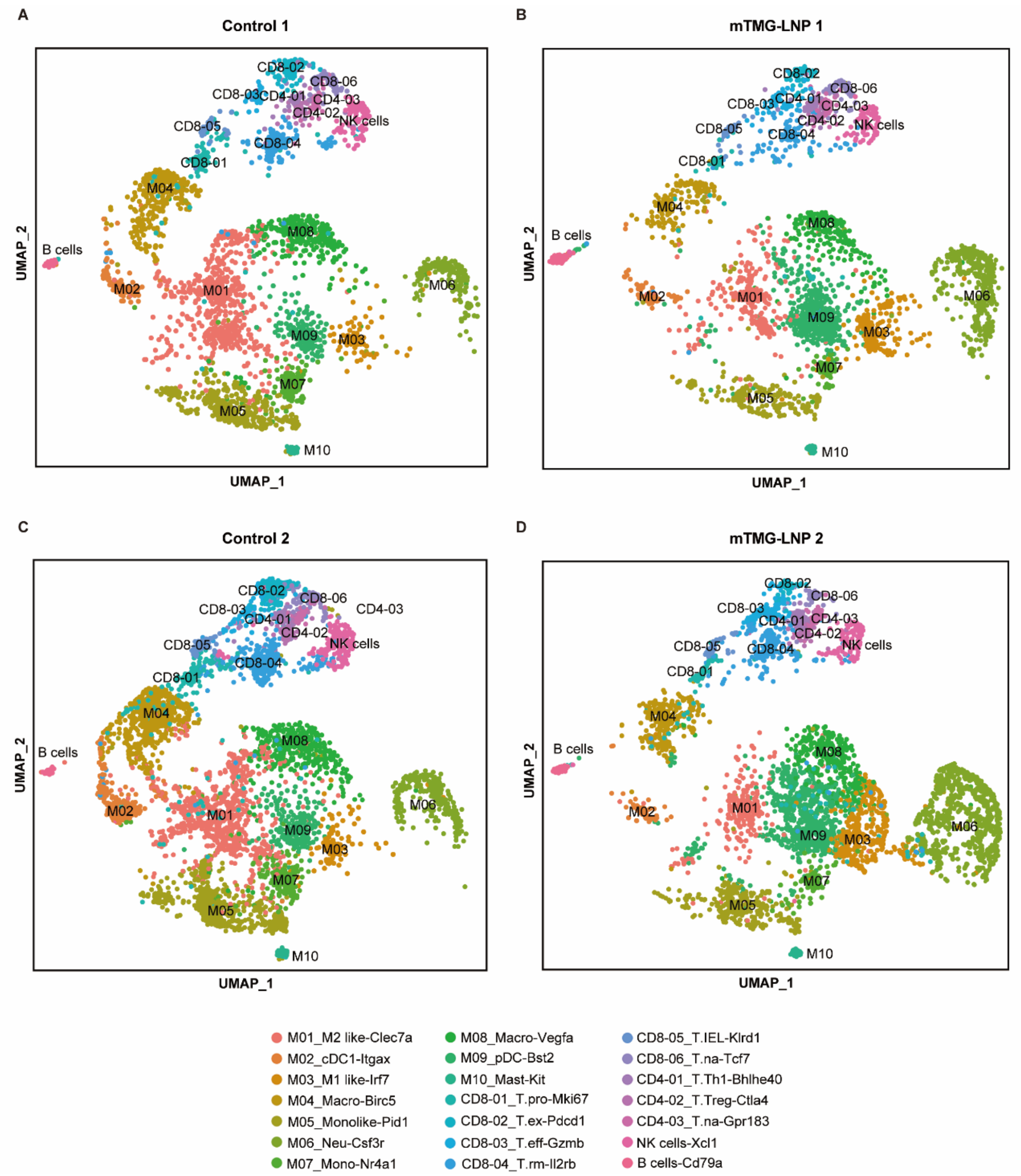

Fig. S12. scRNA-seq analysis revealed remodeling of tumor microenvironment following mRNA vaccine administration. Uniform Manifold Approximation and Projection (UMAP) plot showing respectively Seurat-guided unsupervised clustering and distribution of $CD45^{+}$ cells from the tumor tissue of each one MC38 tumor-burdened mice (n = 4 mice). Each dot denotes an individual cell; same color indicates the same cluster. There are 21 main clusters, including 6 $CD8^{+}$ clusters, 3 $CD4^{+}$ clusters, 10 Myeloid cell clusters and another 2 smaller clusters of immune cells NK and B cells. We selected one representative signature gene to name each cluster and indicate the potential function (bottom).

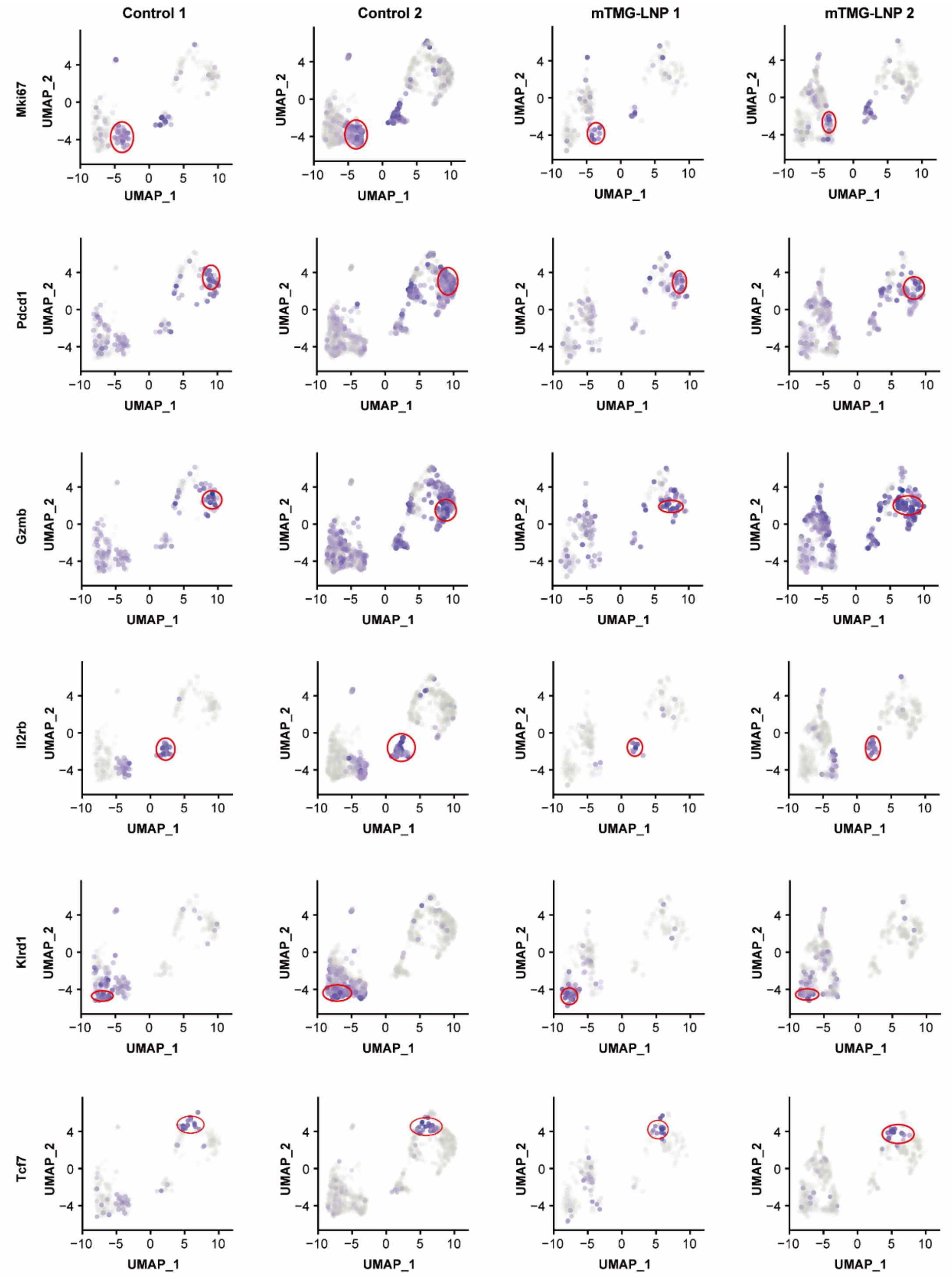

Control 1
Control 2
mTMG-LNP 1
mTMG-LNP 2
Mki67
Pdcd1
Gzmb
Il2rb
Klrd1
Tcf7
UMAP_1
UMAP_2

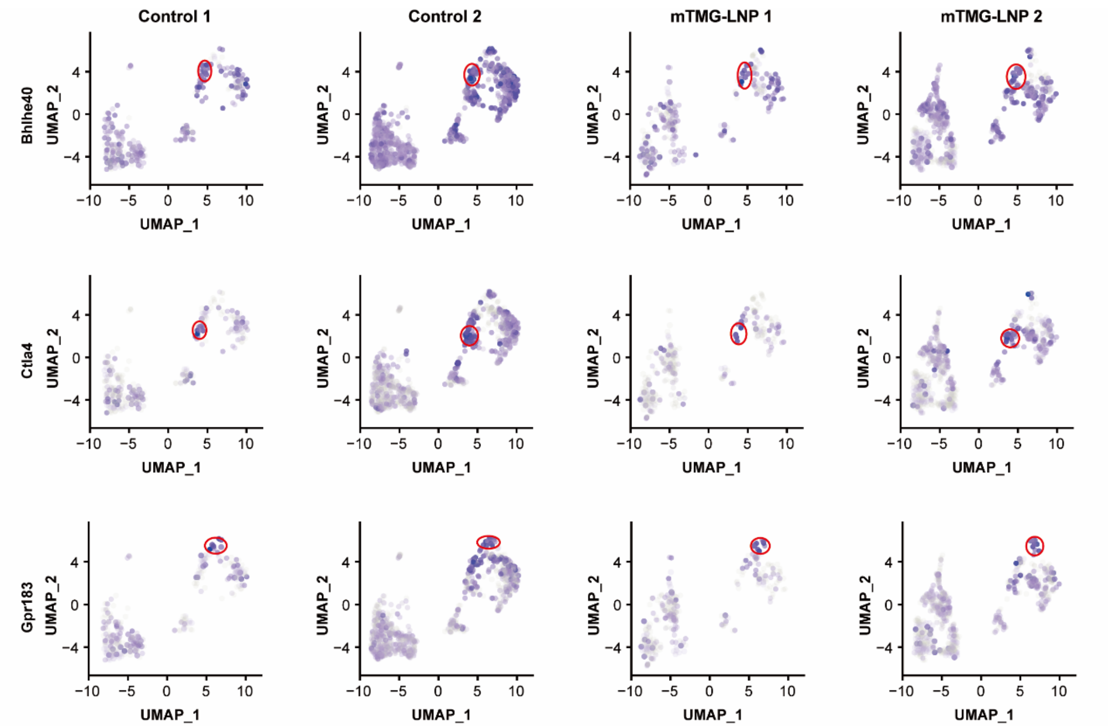

Fig. S13. Expression levels of signature genes in each T cell clusters were shown in each mouse. UMAP plot of expression levels of selected genes in different clusters indicated by the red oval. Each dot denotes an individual cell. Representative 9 T cell clusters, including 6 $CD8^+$ T cell clusters and 3 $CD4^+$ T cell clusters for each control or vaccinated animal separately (n = 4 mice).

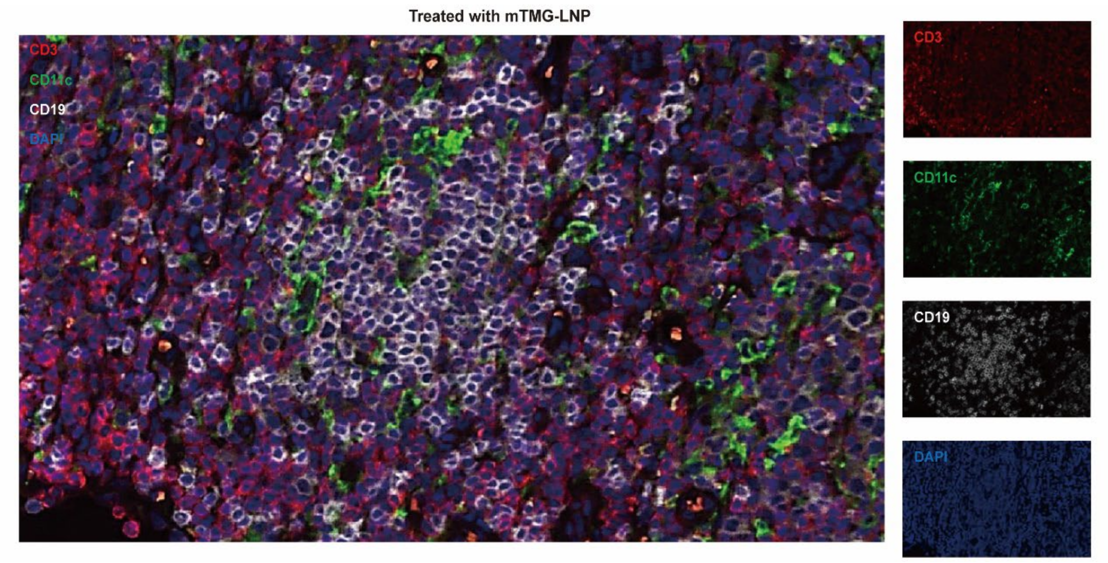

Fig. S14. Observation of tertiary lymphoid structures in tumor microenvironment after MC38 tumor-burdened mice were treated with mRNA vaccine. Multiplex immunohistochemistry (mIHC) investigation of tumor tissues with the following marker: CD3 (T cells), CD11c (DCs), CD19 (B cells) and DAPI (nuclei) (n = 3 mice).

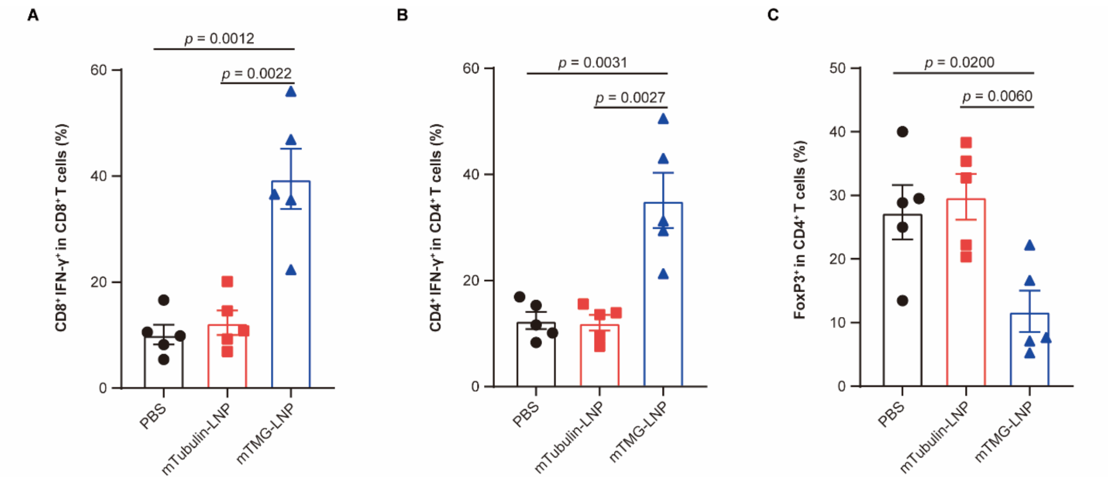

Fig. S15. mRNA vaccines improve the tumor microenvironment. (A to **C)** Proportion of infiltrating cells in sections from different treatment groups. Each section was randomly selected five nonoverlapping vision, the Halo software (3.5, Indica Labs, United States) automatically collecting positive staining cells and the total number of cells (n=5), $CD8^{+}$ IFN-γ^{+} in $CD8^{+}$ T cells (A), $CD4^{+}$ IFN-γ^{+} in $CD4^{+}$ T cells (B), $FoxP3^{+}$ in $CD4^{+}$ T cells (C). Data were shown as mean ± SEM, statistical analysis was performed by one-way ANOVA (H-J).

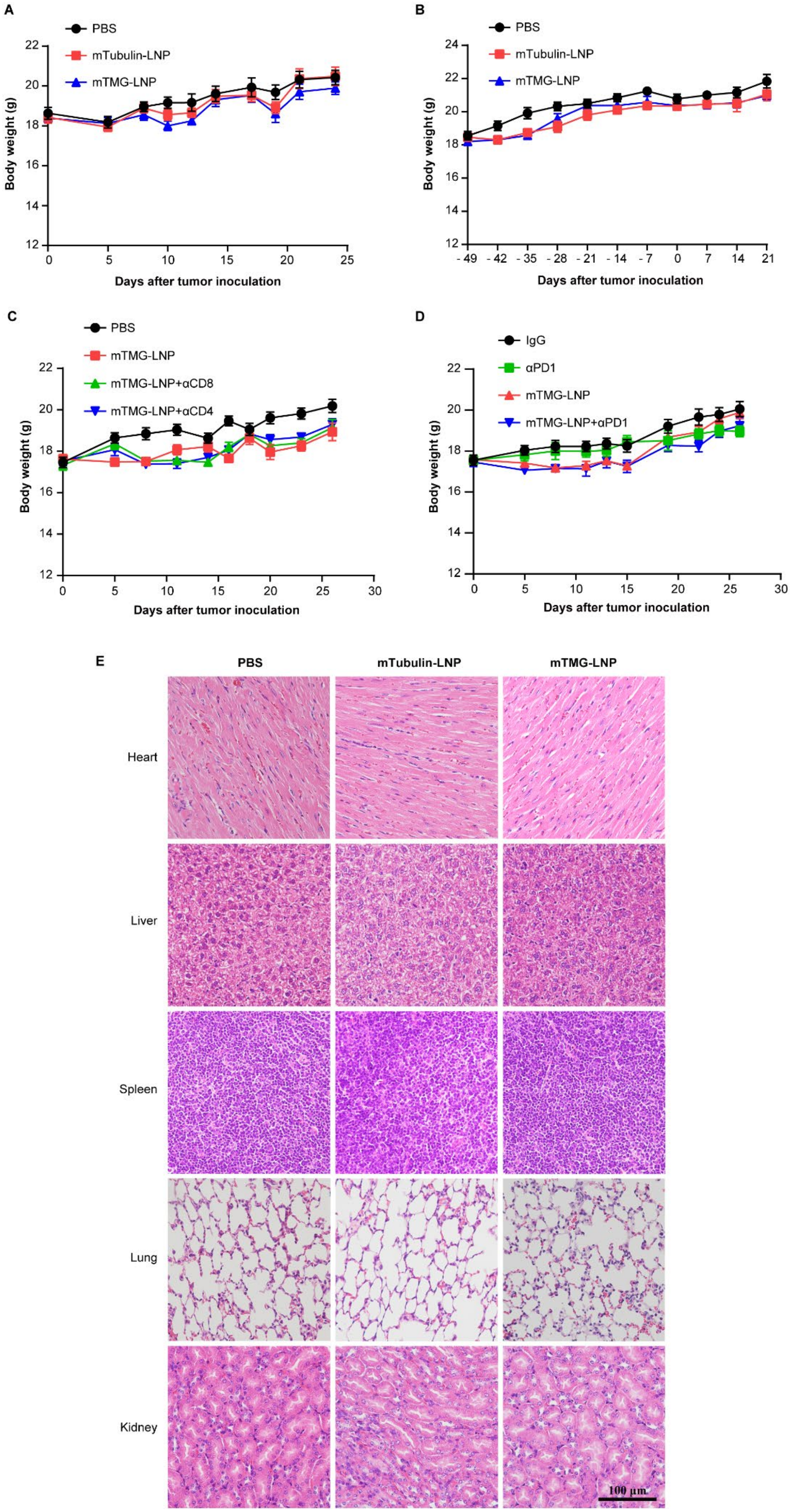
A
PBS
mTubulin-LNP
mTMG-LNP
Body weight (g)
Days after tumor inoculation
B
PBS
mTubulin-LNP
mTMG-LNP
Body weight (g)
Days after tumor inoculation
C
PBS
mTMG-LNP
mTMG-LNP+αCD8
mTMG-LNP+αCD4
Body weight (g)
Days after tumor inoculation
D
IgG
αPD1
mTMG-LNP
mTMG-LNP+αPD1
Body weight (g)
Days after tumor inoculation
E
PBS
mTubulin-LNP
mTMG-LNP
Heart
Liver
Spleen
Lung
Kidney
100 μm

Fig. S16. Safety evaluation of the neoantigen vaccines. (A) Body weight changes when mice were treated prevented with mTMG-LNP vaccines (n =8 mice). **(B)** Body weight changes when mice were prevented with mTMG-LNP vaccines (n =10 mice). **(C)** Body weight changes when mice were treated with mTMG-LNP vaccines while $CD4^+$ or $CD8^+$ T cells were depleted (n = 6 mice). **(D)** Body weight changes when mice were treated synergistically with mTMG-LNP vaccine and aPD-1(n = 7 mice). **(E)** H&E staining of major organs at the end of the experiment in the subcutaneous MC38 tumor model. Scale bar, 100 μm. The data are presented as the mean ± SEM.

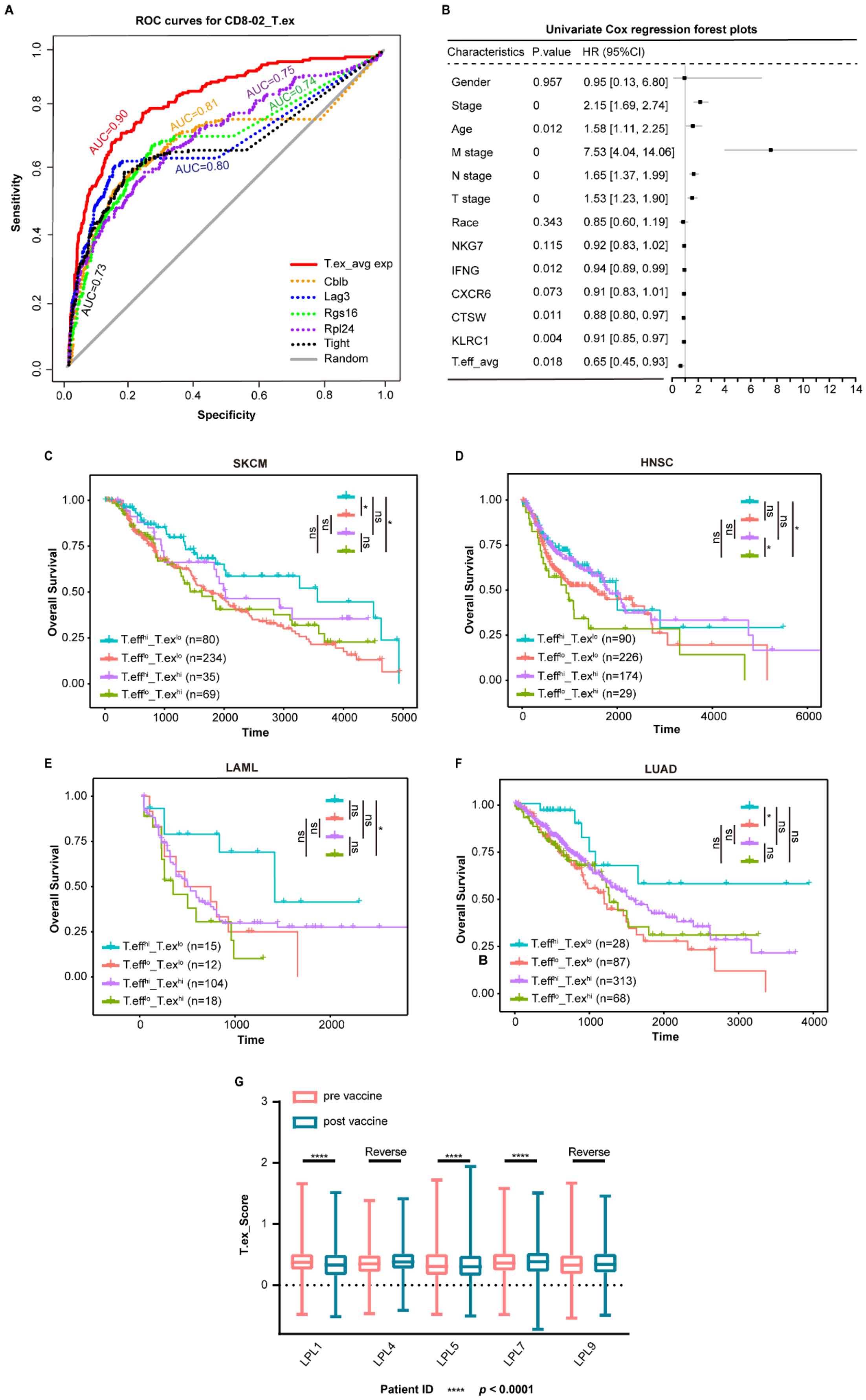

Fig. S17. The differential signature of antigen-specific T cells has been linked to survival of

human tumors. (A) Receiver operating characteristic (ROC) plots showing the performance in prediction of CD8-02 exhausted T cells (AUC=0.90). (**B**) Univariate Cox regression forest plot analysis of the average expression of CD8-03 T.eff cells population representative genes and common gender, age and TNM-staging factors were performed on the BRCA. (**C** to **F**) Kaplan-Meier survival curves of patients from, Skin Cutaneous Melanoma (SKCM, n = 418) (C), Head and Neck squamous cell carcinoma (HNSC, n = 519) (D), Acute Myeloid Leukemia (LAML, n = 149) (E) and Lung adenocarcinoma (LUAD, n = 496) (F) with respect to high or low T.eff/T.ex score within tumor specimens. **(G)** Three of five lymphoplasmacytic lymphoma patients showed lower T.ex scores of $CD8^+$ T cells in post-tumor vaccine samples when comparing to pre-treatment counterparts (GSE243545, one-sided Wilcoxon rank-sum test, LPL1 ($p = 1\times10^{-15}$), LPL4 ($p = 1\times10^{-16}$), LPL5 ($p = 1\times10^{-15}$), LPL7 ($p = 1\times10^{-16}$), LPL9 ($p = 1\times10^{-15}$)). Reverse, Indicates the opposite T.ex score change. Survival distributions were compared using the log-rank test (C-F). * $p < 0.05$, ** $p < 0.01$, **** $p < 0.0001$.

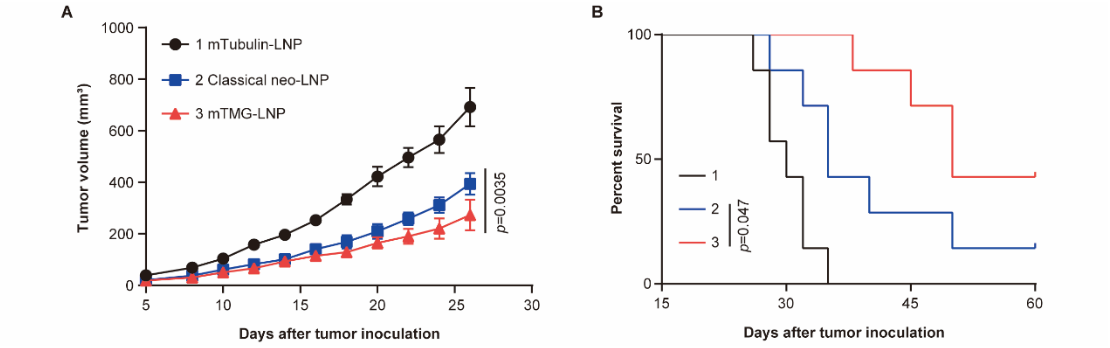

Fig. S18. Comparison of immunogenicity of LSTV-derived epitopes with SNV-derived epitopes. Mice with established MC38 tumors received either a vaccine containing the LSTV-derived epitopes (mTMG-LNP) or a vaccine containing classical SNV-derived epitopes Adpgk, Reps1, and Rpl18 (Classical neo-LNP). Vaccines (20 µg/dose, s.c) were administered subcutaneously every 3 days for a total of 5 immunizations, starting on day 3 after tumor inoculation. Tumor volume was monitored over time and survival analysis was performed. (**A**) MC38 tumor growth curves (n = 7 mice). Values represent mean ± SEM. *p* value was determined by two-way analysis of variance (ANOVA). (**B**) Survival analysis of mice (n = 7 mice). *P* value was determined using a log-rank test (Mantel-Cox).

Supplementary Tables

Table S1. Subtype-specific variants were obtained by using bioinformatics analysis pipeline to analyze the MC38 subcutaneous tumor long-read RNA-seq data

Isoform	Chrom	start	Length	Exons	Strand	Associated.gene	Long reads number	Expression level (TPM)
**PB.349.2**	chr11	119,974,797	4,126	26	-	Cep131	2	2.82
**PB.458.1**	chr13	95,650,591	2,896	3	-	F2rl1	5	9.28
**PB.1517.1**	chr8	41,308,024	2,406	2	-	Gm5345	3	82.79
**PB.1528.1**	chr8	70,905,665	3,136	4	-	Klhl26	2	3.68
**PB.1596.5**	chr8	124,155,108	2,929	2	-	Q1KKYM0	2	39.36

Within the presented table, "Isoform" signifies the unique identifier of these isoform variants, while "Chrom" correspond to the chromosomal identification; "Start", chromosomal position coordinates; "Length", transcript length; "Exons", number of exons within the transcript; "Strand", the gene to which these isoform variants are attributed within the genome; "Associated gene", represents the specific gene information obtained by matching; "Long reads number" quantifies the number of long-length reads that provide support for the identification of these isoform variants, and "Expression level" quantifies the normalized expression level of these variant isoforms as determined through second-generation short-read sequencing analysis.

Table S2. Predict the epitopes in MC38 subcutaneous tumors based on FIONA2 model and verify their immunogenicity

Name	Peptide	Assciated_isoform _variants	MHC	%Rank _EL	Aff(nM)	HomoId	Immunogenicity verification
MC38-M01	IVLLRSYL	PB.458.1-ORF7	H-2-Kb	0.189	57.54	N	
MC38-M02	KTLVYWGL	PB.458.1-ORF7	H-2-Kb	0.4	107.36	N	√
MC38-M03	LVYWGLGL	PB.458.1-ORF7	H-2-Kb	0.56	170.02	N	√
MC38-M04	RSYLRVAKTL	PB.458.1-ORF7	H-2-Kb	1.36	173.88	N	
MC38-M05	VLLRSYLRV	PB.458.1-ORF7	H-2-Kb	0.771	233.5	N	
MC38-M06	ITLLAASV	PB.458.1-ORF7	H-2-Kb	1.077	283.07	N	
MC38-M07	RSYLRVAKT	PB.458.1-ORF7	H-2-Kb	0.92	335.23	N	
MC38-M08	KSPWFTTL	PB.1596.5-ORF5	H-2-Kb	0.013	13.85	N	√
MC38-M09	SSWDYITV	PB.1596.5-ORF5	H-2-Kb	0.049	22.64	N	√
MC38-M10	ATQQFQQL	PB.1596.5-ORF5	H-2-Kb	0.073	158.33	N	
MC38-M11	VSLTLALL	PB.1596.5-ORF5	H-2-Kb	0.081	15.8	N	√
MC38-M12	SGPPYYEGV	PB.1596.5-ORF5	H-2-Kb	0.116	81.5	N	
MC38-M13	LTQQYHQL	PB.1596.5-ORF5	H-2-Kb	0.126	167.35	N	
MC38-M14	VATQQFQQL	PB.1596.5-ORF5	H-2-Kb	0.141	232.98	N	

MC38-M15	LTIRFTSF	PB.1596.5-ORF5	H-2-Kb	0.157	87.91	N	
MC38-M16	KSPWFTTLI	PB.1596.5-ORF5	H-2-Kb	0.264	64.99	N	√
MC38-M17	GAYQALNL	PB.1596.5-ORF5	H-2-Kb	0.297	285.53	N	√
MC38-M18	VTYHSPSYV	PB.1596.5-ORF5	H-2-Kb	0.333	128.5	N	
MC38-M19	VSLTLALLL	PB.1596.5-ORF5	H-2-Kb	0.46	111.14	N	√
MC38-M20	PSYVYHQF	PB.1596.5-ORF5	H-2-Kb	0.474	428.85	N	
MC38-M21	KSITNLEKSL	PB.1596.5-ORF5	H-2-Db	0.012	39.62	N	
MC38-M22	SAPANCSVA	PB.1596.5-ORF5	H-2-Db	0.088	83.11	N	√
MC38-M23	WAITGNHPL	PB.1596.5-ORF5	H-2-Db	0.097	175.03	N	
MC38-M24	SLTIRFTSF	PB.1596.5-ORF5	H-2-Kb	0.641	383.95	N	
MC38-M25	VSGPPYYEGV	PB.1596.5-ORF5	H-2-Kb	1.486	495.91	N	
MC38-M26	LGRYLYNRL	PB.1528.1-ORF4	H-2-Kb	0.651	182.87	N	
MC38-M27	EIWGFHVNL	PB.1517.1-ORF21	H-2-Kb	0.217	160.2	N	√
MC38-M28	IWGFHVNL	PB.1517.1-ORF21	H-2-Kb	0.386	132.96	N	√
MC38-M29	VEIWGFHVNL	PB.1517.1-ORF21	H-2-Kb	1.106	136.45	N	√
MC38-M30	KLVINGKPI	PB.1517.1-ORF12	H-2-Db	0.24	375.87	N	
MC38-M31	LNYMVYMF	PB.1517.1-ORF12	H-2-Kb	0.866	228.36	N	

MC38-M32	SRWFFLASF	PB.1517.1-ORF11	H-2-Kb	0.981	408.06	N	
MC38-M33	RWFFLASFPAAIIRS	PB.1517.1-ORF11	H-2-IAb	1.45	26.1	N	√
MC38-M34	SRWFFLASFPAAIIR	PB.1517.1-ORF11	H-2-IAb	1.62	28.07	N	√
MC38-M35	PSRWFFLASFPAAII	PB.1517.1-ORF11	H-2-IAb	2.95	42.19	N	√
MC38-M36	WFFLASFPAAIIRSI	PB.1517.1-ORF11	H-2-IAb	3.44	43.06	N	√
MC38-M37	WPSRWFFLASFPAAI	PB.1517.1-ORF11	H-2-IAb	6.08	61.02	N	√
MC38-M38	FFLASFPAAIIRSIS	PB.1517.1-ORF11	H-2-IAb	8.35	72.82	N	√
MC38-M39	GPPYYEGVAVLGTYS	PB.1596.5-ORF5	H-2-IAb	0.75	479.56	N	√
MC38-M40	PPYYEGVAVLGTYSN	PB.1596.5-ORF5	H-2-IAb	0.82	483.76	N	√
MC38-M41	SGPPYYEGVAVLGTY	PB.1596.5-ORF5	H-2-IAb	1.04	576.96	N	√
MC38-M42	DGSYYLAAPTGTTWA	PB.1596.5-ORF5	H-2-IAb	1.09	182.23	N	√
MC38-M43	SDGSYYLAAPTGTTW	PB.1596.5-ORF5	H-2-IAb	1.32	230.52	N	√
MC38-M44	TSDGSYYLAAPTGTT	PB.1596.5-ORF5	H-2-IAb	1.47	306.75	N	√
MC38-M45	VSGPPYYEGVAVLGT	PB.1596.5-ORF5	H-2-IAb	1.72	877.51	N	√
MC38-M46	GSYYLAAPTGTTWAC	PB.1596.5-ORF5	H-2-IAb	3.1	253.19	N	√
MC38-M47	SYYLAAPTGTTWACS	PB.1596.5-ORF5	H-2-IAb	9.97	401.98	N	√
MC38-M48	KTSDGSYYLAAPTGT	PB.1596.5-ORF5	H-2-IAb	3.55	544.14	N	√

MC38-M49	PYYEGVAVLGTYSNH	PB.1596.5-ORF5	H-2-IAb	3.41	793.19	N	√

The predicted neoantigen epitopes for tumor neoantigens are presented in the table. Within this table, "Peptide" represents the peptide sequence of the epitope, "Associated.isoform.variants" denotes the isoform variants associated with the expressed epitopes and their corresponding open reading frames (ORF), "MHC" specifies the major histocompatibility complex (MHC) types capable of binding to the epitopes, and "Reactive T cell subtype" identifies the T cell subtypes where these MHC molecules are found. "%Rank.EL", "Aff(nM) ", and "HomoId" respectively indicate the likelihood of these epitopes being predicted as immunogenic (incorporating both FIONA2 and netMHCpan algorithms), the affinity strength of their binding to MHC, and the presence of homologous peptides in the mouse proteome. PB.349.2 has not predicted epitopes and is directly concatenated in the vaccine plasmid.

Table S3. Selected immunogenic epitopes and predicted self-peptides

Name	Epitope	No.	Self-Peptides	Gene symbol	Uniprot ID
MC38-M02	KTLVYWGL	M02_1	KHLVYWSL	S19A3	Q99PL8
		M02_2	KTLRRWGL	5NT1B	Q91YE9
		M02_3	KTLRSWGL	5NT1A	A3KFX0
MC38-M08	KSPWFTTL	M08_1	HSPWFTGL	LMF2	Q8C3X8
		M08_2	QSPGFTTL	GP1BA	O35930
		M08_3	KSPAFTGL	KCD19	Q562E2-2
MC38-M09	SSWDYITV	M09_1	SSWDVPTV	P11928	OAS1A
		M09_2	SSEDKITV	P46656	ADX
		M09_3	SSWDYWTY	Q9D6N1	CAH13

To address the concern regarding potential immunity and cross-reactivity against self-antigens, we selected three peptides with high homology to LSTV-derived epitopes. These peptides were then assessed using in vitro ELISpot assays to evaluate their potential to elicit an immune response.

Table S4. Sample and RNC-seq information

	Sample information			
	Control 1	Vaccine 1	Control 2	Vaccine 2
**Mice counts**	1	1	1	1
**Tissue**	Tumor	Tumor	Tumor	Tumor
**Treatment**	mTubulin-LNP	mTMG-LNP	mTubulin-LNP	mTMG-LNP
**pMHC-1 Tetramer sorting**	No	No	No	No
**Harvest time**	Day 21	Day 21	Day 21	Day 21
**scRNA-seq**	Yes	Yes	Yes	Yes
**scTCR-seq**	Yes	Yes	Yes	Yes
**Sequencing platform**	NovaSeqS4	NovaSeqS4	NovaSeqS4	NovaSeqS4
	**Single cell RNA-seq**			
**Estimated cell number in scRNA-seq**	4,361	4,831	7,367	6,840
**Fraction reads in cells**	90.00%	96.10%	94.00%	96.50%
**Mean reads per cell**	153,470	123,191	50,546	61,449
**Median genes per cell**	2,576	3,580	3,146	3,022
**Total genes detected**	15,727	16,315	16,782	16,945

**Median UMI counts per cell**	7,873	13,153	10,596	10,490
**Number of reads**	299,880,222	336,433,261	372,373,056	420,312,325
**Valid barcodes**	88.94%	89.42%	87.30%	86.70%
	**Single cell TCR-seq**			
**Estimated cell number in scTCR-seq**	214	312	860	384
**Reads mapped to any V(D)J gene**	26.20%	26.10%	40.50%	31.70%
**Reads mapped to TRA**	7.40%	9.00%	14%	11.70%
**Reads mapped to TRB**	18.80%	16.90%	26.00%	19.90%
**Cells with Productive V-J Spaning Pair**	69.80%	79.20%	82.40%	88.50%

Table S5. The summary of 21 cell cluster characteristics

Cell cluster name	Representative genes	Functional properties
CD4-01-T.Th1-Bhlhe40	Bhlhe40, Cxcr6, Tnfsf11, Icos, Skap1, Klrc1, Ifng	$CD4^+$ Tumoral Type1 T helper (T.Th1)
CD4-02-T.Treg-Ctla4	Foxp3, Ctla4, Il2ra, Tnfrsf18, Tnfrsf4, Tnfrsf9, Ikzf2	$CD4^+$ Tumoral regulatory T cells (T.Treg)
CD4-03-T.na-Gpr183	Gpr183, Rora, Cd5, Icos	$CD4^+$ Tumoral naïve (T.na)
CD8-01-T.pro-Mki67	Mki67, Skap1, Ccna2, Rrm2, Cdk1	$CD8^+$ Proliferative T cells (pro)
CD8-02-T.ex-Pdcd1	Pdcd1, Lag3, Rgs16, Tigit, Tnfrsf9	$CD8^+$ Tumoral exhausted (T.ex)
CD8-03-T.eff-Gzmb	Nkg7, Gzmb, Ctsw, Klrc1, Ifng, Cxcr6, Lck	$CD8^+$ Tumoral effector (T.eff)
CD8-04-T.rm-Il2rb	Il2rb, Ctla4, Cd69, Xcl1	$CD8^+$ Tumoral resident memory (T.rm)
CD8-05-T.IEL-Klrd1	Klrc1, Klrd1, Il2rb	$CD8^+$ Tumoral Intraepithelial lymphocytes (T.IEL)
CD8-06-T.na-Tcf7	Tcf7, Skap1, Itk	$CD8^+$ Tumoral naïve (T.na)
M01_M2 like-Clec7a	Clec7a, Ctsc, Fn1, Tgfb1, Mrc1, Mgl2	Cancer-promoting properties
M02_cDC1-Itgax	Itgax, Kmo	Antigen cross-presentation (MHC-I)

M03_M1 like-Irf7	Nos2, Il1a, Il1b, Irf7, Slfn4, Fcgr1	Antitumor properties
M04_Macro-Birc5	Birc5, csf1r	Peripheral tumor-associated macrophages
M05_Mono-like-Pid1	Pid1, Dock4	Peripheral monocyte-like cells
M06_Neu-Csf3r	Retnlg, Csf3r, Mmp9, Cxcr2	Classical neutrophils
M07_Mono-Nr4a1	Nr4a1, Plac8	Peripheral monocytes
M08_Macro-Vegfa	Vegfa, Mmp12, Fabp5, Arg1, Spp1	Peripheral tumor-associated macrophages
M09_pDC-Bst2	Bst2, Siglec1	Secrete Type I IFN
M10_Mast-Kit	Cma1, Mcpt4, Kit	Peripheral tissue-resident cells
NK cells-Xcl1	Xcl1, Txk, Nkg7, Prf1, Fcgr3,	Innate immune lymphocytes
B cells-Cd79a	Cd79a, Cd79b, Cd19	Classical B lymphoid cells

Table S6. List of reagents used in the study

REAGENT or RESOURCE	SOURCE	IDENTIFIER
**Antibodies**		
FITC-CD45 (FC, 30-F11)	BioLegend	Cat# 157214
BV711-F4/80 (FC, BM8)	BioLegend	Cat# 123147
APC-CD11b (FC, M1/70)	BioLegend	Cat# 101211
BV711-CD3 (FC, 17A2)	BioLegend	Cat# 100241
PE-CD8 (FC, 53-6.7)	BioLegend	Cat# 100706
BV605-CD4 (FC, RM4-5)	BioLegend	Cat# 100547
BV421-CD86 (FC, GL-1)	BioLegend	Cat# 105032
PE/Cy7-CD206 (FC, C068C2)	BioLegend	Cat# 141720
BV605-CD11C (FC, N418)	BioLegend	Cat# 117334
FITC-CD25 (FC, 3C7)	BioLegend	Cat# 101907
Alexa Fluor® 647-Foxp3 (FC, MF-14)	BioLegend	Cat# 126407
PE-Ly-6G (FC, S19018G)	BioLegend	Cat# 164503
PE/Cy7-Ly6C (FC, HK1.4)	BioLegend	Cat# 128017
BV510-IFN-γ (FC, XMG1.2)	BioLegend	Cat# 505842

FITC-TNF-a (FC, MP6-XT22)	BioLegend	Cat# 506303
BV711-CD62L (FC, MEL-14)	BioLegend	Cat# 104445
PE/Cy7-CD44 (FC, IM7)	BioLegend	Cat# 103029
Zombie Violet™ Fixable Viability Kit	Biolegend	Cat# 423113
H-2Kb Neoepitope Tetramer-KTLVYWGL-PE	GL	Cat# GL-P18-1
H-2IAd Neoepitope Tetramer-GPPYYEGVAVLGTYS-PE	GL	Cat# GL-P18-2
CD16/CD32 (clone 93)	Biolegend	Cat# 101319
7-AAD Viability Staining Solution	BioLegend	Cat# 420404
Anti-IFN-r (IF, JM10-10)	Novus	Cat# NBP2-66900
Anti-CD3 (IF, SP7)	Abcam	Cat# Ab5690
Anti-CD8 (IF, EPR21769)	Abcam	Cat# Ab217344
Anti-CD4 (IF, EPR19514)	Abcam	Cat# Ab183685
Anti-Foxp3 (IF, EPR22102-37)	Abcam	Cat# Ab215206
Anti-CD8 (In vivo, YTS169.4)	Bio X cell	Cat# BE0117
Anti-CD4 (In vivo, YTS177)	Bio X cell	Cat# BE0003-3
Anti-PD1 (In vivo, RMP1-14)	Bio X cell	Cat# BE0146
InVivoMab rat IgG2a isotype control	Bio X cell	Cat# BE0089

InVivoMab rat IgG2b isotype control	Bio X cell	Cat# BE0090
**Chemicals, Peptides, and Recombinant Proteins**		
Ionizable lipid (SM102)	AVT	Cat# SM211208
1,2-distearoyl-sn-glycero-3-phosphocholine (DSPC)	AVT	Cat# B90536
1,2-dimyristoyl-rac-glycero-3-methoxypolyethylene glycol-2000 (DMG-PEG2K)	AVT	Cat# EDM210824
Cholesterol	AVT	Cat# B90867
Neoantigen peptide (all peptides in Table S2)	Sangon	N/A
Phorbol 12-myristate-13-acetate (PMA)	Sigma-Aldrich	Cat# P8139
Ionomycin	Sigma-Aldrich	Cat# I0634
RPMI-DMEM	Gibco	Cat# 12633012
Collagenase type IV	Sigma	Cat# C0130
DNase I	Roche	Cat# 11284932001
**Critical Commercial Assays**		
Mouse IFN-γ ELISPOT Sets	Dakewe	Cat# 2210005
Leucocyte isolation fluid from tumor infiltrating tissue kit	Solarbio	Cat# P1910
MACS Tumor Dissociation Kit	Miltenyi Biotec	Cat# 130-095-929

Chromium Single Cell 5' Library and Bead Kit	10 x Genomics	Cat# 1000006
Chromium Single Cell 5' Library Construction Kit	10 x Genomics	Cat# 1000020
TNF-α Mouse Uncoated ELISA Kit	eBioscience	Cat# 88-7324-77
IL-6 Mouse Uncoated ELISA Kit	eBioscience	Cat# 88-7064-86
IL-1β Mouse Uncoated ELISA Kit	eBioscience	Cat# 88-7013-86
TGFβ-1 Mouse Uncoated ELISA Kit	eBioscience	Cat# 88-8350-88
**Experimental Models: Cell Lines**		
MC38	ATCC	ENH204-EP
B16-F10	ATCC	CL-0319
**Experimental Models: Organisms/Strains**		
C57BL/6J	Vital River Laboratory	N/A

Table S7. The sequences of the five isoforms

Isoforms	Sequences
PB.349.2_Cep131	AGCCGCCGCCGCCGCCGCTCGCCCCTGCGGACAGCGTTTAT<br>TGTCACCCACGGCGGAGGCCGCGCCAGCCCCGTGGGCCCC<br>GGGTGGCTCTGCAGCCGCGCCCGCCAGGCCGAGCCCACGGG<br>CCCTGCGCCCCAGGCTAGGTCCGCGCAGGTGAGGGGCGTCT<br>CGCGGTGAAGGGTCGGGCCCTGCAGCGAGGTCGGCCTGTGC<br>CCAGTACCCCCAGCCCCGGAAGGTCGCCGTGGGCTCCATTC<br>GGGCGGCCCGGAACCCCTGCCTTAAACCTCAAGAGACCTAG<br>GGG**ATGCGGGTCGCCCGCGGAGGAGCACAGCAGTGCGC**<br>**TGCTCAAGGGCTCTGCCTCTGCGAGTCAGCTGGCCCCCG**<br>**AGGGAGCAGGCTCCTGCCCCTCCGGCTCCTGCGGGAGG**<br>**CGCGGTTTTGTCCAGGGTGA**GGCTGGCCTCCCCAGCCTCC<br>GGCCATCTGGCTTATAGATTTCC**ATGGAGAGGCTATGTTTAC**<br>**CGCCCAGAACCTCACTGGCTGCACGTGGTGCTGGGAAA**<br>**GCTATGTCCACCAGACTTAGGAGCTTAGGATCCACCAAG**<br>**GTGTAG**CCAGTGCCAAGAAGGGCAAGGTATTAGGTTTCACC<br>TACACAACCAGAAATGTGTAACGGGGACACTGGCCGGACTG<br>ATGGCAAAGCAGCCTCAGAGTAGGGACACTGCCCCCTACTA<br>CACACATCTGGATGTCAGTACCAAGGCAGCTTGACTTTGGAC<br>CATGGGTTAGCCTGGTCCACCATGAAAGGTTCACGGACCATC<br>ACTGCCACTCC
PB.458.1_F2rl1	AGTGAAGCTCGTGTTAAGGGAAGGGACCCTGTGCTCAGAGT<br>AGGGCTCCGAGTTTCGAACCACTGGTGGCGGATTGCCCGCC<br>CGCCCCACGTCCGGGG**ATGCGAAGTCTCAGCCTGGCGTG**<br>**GCTGCTGGGAGGTATCACCCTTCTGGCGGCCTCGGTCTC**<br>**CTGCAGCCGGACCGAGAACCTTGCACCGGCTTCCCTATG**<br>**TCTTGTTAAAAGACCATTGTCATTGGACCGAGGGCCTGA**<br>**CTGGATAACCTGGATAGTGTTGTTAAGATCGTACCTTAGG**<br>**GTTGCAAAGACCCTTGTCTACTGGGGATTAGGACTTGAA**<br>**CACCTTCTTTTTGGGACGCAACAACAGTAAAGGAAGAA**<br>**GTCTTATTGGCAGATTAG**AAACCCAGCCTCCAATCACTGGG<br>AAA
PB.1517.1_Gm5345	GTGCTCGCGACCCTCGCCTGGAGCGTTTGTCACGGCTCGGC<br>GGGCGGGCGCGCAGAGACTCCAGGAAGGCGGTGTCTAACTC<br>CAAGCTTCTGAAAGCCTGCTTTAAGGATATCCACGTGTTCAA<br>TATGCTTGCTCGAAGTTTGGCCCACACCAAATGGTTGTTTAA<br>CCCCAAATATCTGAGCAGCAAACTGAGCTGATCCTTCTGGAG<br>AAAGTATAGTTGAACAGCCAGCCAAGACCACTGGGAAGTCG<br>AAGAGATGACCACACATCCTGAGCGCCCCAGTCTGGTGTGA<br>TTGGGGGACAGCTGATAACCAGATATACAGTATTACCAGACA<br>TCACTGGGCCTAGACCGTTGCTTCTGCCCGCCATTGTGACAA<br>ATACAGTAGCTATGCCATCCGCTTCATATTCTGCTTTAATCCTC<br>AAAGCCTCATCTGGTCCTTTATGTGTGGATG**TTACTCTAAGT**

TCACATGGAATCCCCAAATTTCCACAAGCCTTCTTAATTT
TTTTTCACAGTGACCAAGGTCAGAGGTGGAGCCCATCAG
CACTACCACCCTGCACTGAGTGACTGACTTGAGAAGCAA
CTCCACTCGATCTGCAACCCACTCAGAGTCTTTCTTTACC
ATCTGGAGTCCTTCCGGAGTTACTTCTTTGAGATCATGGT
AAGACTGTTTGTCTTTCTGCTGGCTCCGATCTCCCGATG
GCCATAGTCTCCAGGAACCATTATCAATGGCATCAGCAAG
GACAATCTCTTTGGTGGTTACATCAATGCCAAGTTCAATC
TTCATATCAACCAGCATACAGTCCTGGGGCAGCCAGGAT
TTCTCCAGAATTTCAAAAATAGCTTGTGTGGCATGACTCA
TGATGTCCACTTCAGTCTGCCCTATAACAAGGCCAACAAAGC
ACAATTCTGCAGCGATGAGCTGCTCCTCAGGCCACTGTGCAT
CATTACTGGCATCATCCTTGAAGAACGTCTCCACTTTTGGTA
GATAAAACTTACACGCCTCCTTTACCCCAGGGTTCCTTTTGA
GAAAAGATCCAGTTGCTAGTCTTTGCCATACCTATTCAATGG
AATCATTTCACATCGGGGTGCAATGAAAGCAGTCTCCCCAAA
TTTCTTGGTGAAAGCAGTTTTTATACCAGCTTCCTGTCACAA
CTGAAAT**ATGCAGCTGGTGATCTTATTGGCGACGGCAGCT**
TGGCCTTCCAGGTGGTTCTTTCTAGCTTCATTCCCTGCTG
CAATCATTAGAAGTATAAGTGATATTCCTGATCTTCCTTCC
TCAAGAGATCGCAGGGCTGGGTTTGGTGTGCCATCATCC
TTGGGTGGTTTGAGGTTTGATTTCTTAGAAAGATGGAAA
GAAGGCTGAGTCGTTGTCTCTCTGCTGCTCCCTGTTCCAGA
GACAGCCGCATCTTCTTGTGCAGTGCCAGCCTTGTCCTGTAG
ACAAGATGGTGAAGATCGGTGTGAATGGATTTGGCTGAACT
GG**ATGCCTGGTTACCAGGGTTACCTGGTTGCCTTCTGCT**
CTGAACTTGGCAAAATGGAGATTGTTGCCATCAACGACC
CCTTCATTGACCTCAACTACATGGTCTACATGTTCCAGGA
TGACTCCACTCATGGCAAATTCAACGGCACAGTCAAGGC
TGAGAAAGGGAAGCTTGTCATCAATGGGAAGCCCATCAC
CATCTTCCAGGAGCAAGATCCCAATAACATCAAATGGGG
TGATGCTGGTGCTGAGTATATTTGGAGTCTACTGGTGTCT
TCACCATCATGGAGAAGGCCAGGGCCCACCTGAAGGGTG
GAGCCAAAAGGGTCATCATCTCCACCCCTTCTGCCGATGCCC
CCATGTTTGTGATGGGTGTGAACCAGGAGAAATATGACAACT
CACTCAAGATTGTTAGCATGCATCCTGCATCACCAACTGCTT
AGCTTCCCTGGCCAAGGTCATCCATGACAACTTTGGCATTGT
GGAAGGGTTCGTGACCATGGTCCATGCCATCACTGCCACTCA
GAAGAAGACTGTGAATGGCCCCCCTGGAAAGCTGTGTCAAG
ATGACCGTGGGGCTGCCCAGAACATCATCCCTGCATTCACTG
GTGCTGCCAAAGCTGTGGGCAAGGTCATCCCAGAGCTGAAC
GGGAAGCTCACTGGCATGGCCTCCCTTGTTCCTACTCCCAAT
GTATCCATTGTGGATCTGACATAACGCCTGGAGAAACCTGCC
AAGTATGATGACATCAAGAAGGTGGTGAAGACGGGCAGTGG

	TGGCACACGCCTTTAATCCCAGTACTTGGGAGGCAGAGGCA<br>GGCAGATTTCTGAGTTCGAGGCTAGCCTGGTCTACAGCGTGA<br>GTTCCAGGACAGCCAGGGCTACACAGAGAAACCCTGTCTCA<br>AAACAAAAACAAAAAAACAAAAACAAAAACAAAAAA
PB.1528.1<br>_Klhl26	GCCCCATCGCTCGGGCGCGGCGGAAG**ATGGCGGAGTCC**<br>**GGTGGCAGCAGCGGCTCTAGTCAGAGCCCCGAGCGCCC**<br>**GAGCAGACAGTCCCACTGTGTGGGCCTGGCTGCTCTGG**<br>**GACGCTATCTGTACAACAGGCTGGCCTCTGACTCATAG**A<br>AATCCACCTGCCTCTGCCTTCTGAGTGCTGAGATTAAAGCTT<br>GGCCGACAGGAATGGGGCACTTAAGTGCACCTTCTCTGCAG
PB.1596.5<br>_Q1KKY<br>M0	GCGCCAGTCCTCCGATAGACTGAGTCGCCCGGGTACCCGTGT<br>ATCCAATAAAGCCTTTTGCTGTTGCATCCGAATCGTGGTCTCG<br>CTGATCCTTGGGAGGGTCTCCTCAGAGTGATTGACTGCCCAG<br>CTTGGGGGTCTTTCATTTGGGGGCTCGTCCGGGATTTGGAGA<br>CCCCCGCCCAGGGACCACCGACCCACCGTCGGGAGGCCCTC<br>CAAACGGTGCAGCGAGAAATTTGGAAACCACTGGCCGAGGC<br>CTACCGGGACCGACTAGACCAACCAGTGATACCGCACCCCTT<br>CCGGATTGGAGACTCCGTGTGGGTGCGCCGGCACCAGACCA<br>AAAACTTAGAACCTCGCTGGAAGGGACCCTACACCGTCCTA<br>CTGACCACCCCCACCGCTCTCAAGGTAGACGGCATCTCTGC**A**<br>**TGGATACACGCCGCCCACGTCAAGGCAGCGACCACACCC**<br>**CCGATAAAACCATCATGGAGAGTACAACGCTCTCAAAAC**<br>**CCTTTAAAAATCAGGTTAACCCGTGGGGCCCCCTAATTGT**<br>**CCTTCTGATTCTCGGAGGGGTCAACCCCGTTGCGTTGGG**<br>**AAACAGCCCCCACCAGGTTTTTAACCTCTCCTGGGAAGT**<br>**GACTAATGGAGACCGAGAAACGGTGTGGGCAATAACCGG**<br>**CAATCACCCTCTGTGGACTTGGTGGCCTGACCTCACACC**<br>**AGATCTCTGTATGTTGGCCCTCCACGGGCCGTCCTATTG**<br>**GGGCCTAGAATATCGGGCTCCTTTTTCTCCTCCCCCGGG**<br>**GCCCCCCTGCTGTTCAGGAAGCAGCGACTCCACGCCAG**<br>**GCTGTTCCAGAGATTGTGAGGAGCCCCTGACTTCATATA**<br>**CTCCCCGGTGCAATACGGCCTGGAACAGACTTAAGTTAT**<br>**CTAAAGTGACACATGCCCACAATGAAGGATTCTATGTCTG**<br>**CCCCGGGCCACATCGCCCCCGGTGGGCCCGGTCGTGTG**<br>**GTGGTCCAGAATCCTTCTATTGTGCCTCTTGGGGCTGCG**<br>**AAACCACAGGCCGAGCATCCTGGAAACCATCCTCGTCCT**<br>**GGGACTACATCACAGTAAGCAACAATCTAACCTCAGACC**<br>**AGGCAACCCCAGTATGCAAAGGTAATGAGTGGTGCAACT**<br>**CCTTAACTATCCGGTTCACGAGCTTTGGAAAACAGGCCA**<br>**CCTCCTGGGTCACAGGCCATTGGTGGGGATTGCGCCTAT**<br>**ACGTCTCTGGACATGACCCAGGGCTCATCTTTGGGATCC**<br>**GACTTAAAATTACAGACTCGGGGCCCCGGGTCCCAATAG**<br>**GGCCAAACCCCGTCTTGTCAGACCGACGACCACCTTCCC**<br>**GGCCTAGACCCACCAGATCTCCCCCGCCTTCAAACTCCA**

CCCCAACCGAGACACCCCTCACCCTCCCCGAACCCCCGC
CAGCGGGAGTCGAAAACCGATTGTTAAATCTAGTAAAAG
GAGCCTACCAAGCCCTCAACCTCACCAGTCCTGATAAAA
CCCAAGAGTGCTGGTTATGCCTAGTATCGGGACCCCCAT
ACTACGAGGGGGTTGCCGTCCTAGGTACCTACTCCAACC
ATACTTCTGCCCCAGCTAACTGCTCTGTGGCCTCTCAACA
CAAATTGACCTTGTCCGAAGTGACCGGACAGGGACTCTG
CATAGGAGCGGTCCCTAAAACCCATCAAGTCTTGTGTAAT
ACCACCCAAAAGACAAGCGATGGGTCCTACTATTTGGCC
GCTCCCACAGGAACTACCTGGGCTTGTAGTACTGGACTC
ACTCCCTGTATCTCAACCACCATACTTGACCTCACCACCG
ATTACTGTGTCCTGGTCGAGCTTTGGCCAAGGGTGACCT
ACCATTCCCCTAGTTATGTTTACCACCAATTTGAAAGACG
AGCCAAATATAAAAGAGAACCCGTCTCACTAACTCTGGC
CCTACTATTAGGAGGACTCACTATGGGCGGAATTGCCGC
TGGAGTGGGAACAGGGACTACCGCCCTAGTGGCCACTC
AGCAGTTCCAACAACTCCAGGCTGCCATGCACGATGACC
TTAAAGAAGTTGAAAAGTCCATCACTAATCTAGAAAAATC
TTTGACCTCCTTGTCCGAAGTAGTGTTACAGAATCGTAGA
GGCCTAGATCTACTATTCCTAAAAGAGGGAGGTTTGTGT
GCTGCCTTAAAAGAAGAATGCTGTTTCTATGCCGACCAC
ACAGGATTGGTACGGGATAGCATGGCCAAACTTAGAGAA
AGATTGAGTCAGAGACAAAAGCTCTTTGAATCCCAACAA
GGGTGGTTTGAAGGGCTGTTTAATAAGTCCCCTTGGTTC
ACCACCCTGATATCCACCATCATGGGTCCCCTGATAATCC
TCTTGTTAATTTTACTCTTTGGGCCTTGTATTCTCAATCG
CCTGGTCCAGTTTATCAAAGACAGGATTTCGGTAGTGCA
GGCCCTGGTTCTGACTCAACAATATCATCAACTTAAGACA
ATAGGAGATTGTAAATCACGTGAATAAAGATTTTATTCAG
TTTACAGAAAGAGGGGGGAATGAAAGACCCCTTCATAAGGC
TTAGCCAGCTAACTGCAGTAACGCCATCTTGCAAGGCATGGG
AAAATACCAGAGCTGATGTTCTCAGAAAAACAAGAACAAGG
AAGTACAGAGAGGCTGGAAAGTACCGGGACTAGGGCCAAA
CAGGATATCTGTGGTCAAGCACTAGGGCCCCGGCCCAGGGC
CAAGAACAGATGGTCCCCAGAAATAGCTAAAACAACAACAG
TTTCAAGAGACCCAGAAACTGTCTCAAGGTTCCCCAGATGA
CCGGGGATCAACCCCAAGCCTCATTTAAACTAACCAATCAGC
TCGCTTCTCGCTTCTGTACCCGCGCTTATTGCTGCCCAGCTCT
ATAAAAAGGGTAAAAACCCCACACTCGGCGCGCCAGTCCTC
CGATAGACTGAGTCGCCCGGGT

The sequences provided represent five obtained large-scale transcript variants (LSTVs), wherein the highlighted regions denote the predicted open reading frames (ORFs) derived from the aforementioned analysis.

The GMST algorithm (*49*) was employed to predict open reading frames (ORFs) in the obtained large-scale translated variants (LSTVs), with parameters set to consider: 1) only the direct strand of the cDNA; 2) AUGs as the initial codons and UAA, UAG, UGA as stop codons, and 3) a minimum ORF length of 75 bp. The command utilized was "perl " + GMSP_PROG + " -faa-strand direct-fnn-output {o}". Notably, since GMST typically yields a single ORF prediction, relying solely on this output for certain transcripts lacking a notably longer ORF, such as PB.1571.1, could be precarious. Consequently, we supplemented our analysis with the ORFINDER web tool (*50*) and selected the second-longest ORF for such cases. Although this predictive approach entails a degree of arbitrariness, empirical evaluations (*51*) have indicated a relatively high accuracy.

Other Supplementary Material legend

Data S1. Cluster-specific markers. For each cluster, only the genes that were expressed by more than 30% of total cells with *p*-value < 0.05 were considered.

Data S2. The absolute cell counts in each of the cell clusters. Absolute cell counts are given for all 4 animals and 21 main cell clusters (n = 4 mice).

Data S3. TCR_groups. The sequencing data of both scRNA and scTCR were integrated, if at least two cells shared an identical productive Alpha-Beta pair, those Alpha-Beta pairs were defined as clonal TCRs.

Data S4. Clonal fraction of 9 TCR groups. The degree of clonal expansion of T-cell clusters was quantified using clonogenic scoring (CS). CS was calculated on the well-described TCR clonogenic metric using normalized Shannon entropy. The TCR repertoire diversity of 9 major T cell clusters in the vaccine and Control groups was characterized, respectively.

Data S5. KEGG enriched pathways for CD8_03. Differential pathways enriched for the discriminative markers of CD8-03 T cell subset by KEGG.

REFERENCES AND NOTES

1. P. A. Ott, S. Hu-Lieskovan, B. Chmielowski, R. Govindan, A. Naing, N. Bhardwaj, K. Margolin, M. M. Awad, M. D. Hellmann, J. J. Lin, T. Friedlander, M. E. Bushway, K. N. Balogh, T. E. Sciuto, V. Kohler, S. J. Turnbull, R. Besada, R. R. Curran, B. Trapp, J. Scherer, A. Poran, D. Harjanto, D. Barthelme, Y. S. Ting, J. Z. Dong, Y. Ware, Y. Huang, Z. Huang, A. Wanamaker, L. D. Cleary, M. A. Moles, K. Manson, J. Greshock, Z. S. Khondker, E. Fritsch, M. S. Rooney, M. DeMario, R. B. Gaynor, L. Srinivasan, A phase Ib trial of personalized neoantigen therapy plus anti-PD-1 in patients with advanced melanoma, non-small cell lung cancer, or bladder cancer. *Cell* **183**, 347–362.e24 (2020).

2. U. Sahin, E. Derhovanessian, M. Miller, B.P. Kloke, P. Simon, M. Löwer, V. Bukur, A. D. Tadmor, U. Luxemburger, B. Schrörs, T. Omokoko, M. Vormehr, C. Albrecht, A. Paruzynski, A. N. Kuhn, J. Buck, S. Heesch, K. H. Schreeb, F. Müller, I. Ortseifer, I. Vogler, E. Godehardt, S. Attig, R. Rae, A. Breitkreuz, C. Tolliver, M. Suchan, G. Martic, A. Hohberger, P. Sorn, J. Diekmann, J. Ciesla, O. Waksmann, A.K. Brück, M. Witt, M. Zillgen, A. Rothermel, B. Kasemann, D. Langer, S. Bolte, M. Diken, S. Kreiter, R. Nemecek, C. Gebhardt, S. Grabbe, C. Höller, J. Utikal, C. Huber, C. Loquai, Ö. Türeci, Personalized RNA mutanome vaccines mobilize poly-specific therapeutic immunity against cancer. *Nature* **547**, 222–226 (2017).

3. L. A. Rojas, Z. Sethna, K. C. Soares, C. Olcese, N. Pang, E. Patterson, J. Lihm, N. Ceglia, P. Guasp, A. Chu, R. Yu, A. K. Chandra, T. Waters, J. Ruan, M. Amisaki, A. Zebboudj, Z. Odgerel, G. Payne, E. Derhovanessian, F. Müller, I. Rhee, M. Yadav, A. Dobrin, M. Sadelain, M. Łuksza, N. Cohen, L. Tang, O. Basturk, M. Gönen, S. Katz, R. K. do, A. S. Epstein, P. Momtaz, W. Park, R. Sugarman, A. M. Varghese, E. Won, A. Desai, A. C. Wei, M. I. D’Angelica, T. P. Kingham, I. Mellman, T. Merghoub, J. D. Wolchok, U. Sahin, Ö. Türeci, B. D. Greenbaum, W. R. Jarnagin, J. Drebin, E. M. O’Reilly, V. P. Balachandran, Personalized RNA neoantigen vaccines stimulate T cells in pancreatic cancer. *Nature* **618**, 144–150 (2023).

4. N. Xie, G. Shen, W. Gao, Z. Huang, C. Huang, L. Fu, Neoantigens: Promising targets for cancer therapy. *Signal Transduct. Target. Ther.* **8**, 9 (2023).

5. K. I. Hanada, C. Zhao, R. Gil-Hoyos, J. J. Gartner, C. Chow-Parmer, F. J. Lowery, S. Krishna, T. D. Prickett, S. Kivitz, M. R. Parkhurst, N. Wong, Z. Rae, M. C. Kelly, S. L. Goff, P. F. Robbins, S. A. Rosenberg, J. C. Yang, A phenotypic signature that identifies neoantigen-reactive T cells in fresh human lung cancers. *Cancer Cell* **40**, 479–493.e6 (2022).

6. L. De Mattos-Arruda, M. Vazquez, F. Finotello, R. Lepore, E. Porta, J. Hundal, P. Amengual-Rigo, C. K. Y. Ng, A. Valencia, J. Carrillo, T. A. Chan, V. Guallar, N. Mc Granahan, J. Blanco, M. Griffith, Neoantigen prediction and computational perspectives towards clinical benefit: Recommendations from the ESMO Precision Medicine Working Group. *Ann. Oncol.* **31**, 978–990 (2020).

7. T. Carvalho, Personalized anti-cancer vaccine combining mRNA and immunotherapy tested in melanoma trial. *Nat. Med.* **29**, 2379–2380 (2023).

8. D. B. Keskin, A. J. Anandappa, J. Sun, I. Tirosh, N. D. Mathewson, S. Li, G. Oliveira, A. Giobbie-Hurder, K. Felt, E. Gjini, S. A. Shukla, Z. Hu, L. Li, P. M. le, R. L. Allesøe, A. R. Richman, M. S. Kowalczyk, S. Abdelrahman, J. E. Geduldig, S. Charbonneau, K. Pelton, J. B. Iorgulescu, L. Elagina, W. Zhang, O. Olive, C. McCluskey, L. R. Olsen, J. Stevens, W. J. Lane, A. M. Salazar, H. Daley, P. Y. Wen, E. A. Chiocca, M. Harden, N. J. Lennon, S. Gabriel, G. Getz, E. S. Lander, A. Regev, J. Ritz, D. Neuberg, S. J. Rodig, K. L. Ligon, M. L. Suvà, K. W. Wucherpfennig, N. Hacohen, E. F. Fritsch, K. J. Livak, P. A. Ott, C. J. Wu, D. A. Reardon, Neoantigen vaccine generates intratumoral T cell responses in phase Ib glioblastoma trial. *Nature* **565**, 234–239 (2019).

9. M. Yadav, S. Jhunjhunwala, Q. T. Phung, P. Lupardus, J. Tanguay, S. Bumbaca, C. Franci, T. K. Cheung, J. Fritsche, T. Weinschenk, Z. Modrusan, I. Mellman, J. R. Lill, L. Delamarre, Predicting immunogenic tumour mutations by combining mass spectrometry and exome sequencing. *Nature* **515**, 572–576 (2014).

10. M. Peng, Y. Mo, Y. Wang, P. Wu, Y. Zhang, F. Xiong, C. Guo, X. Wu, Y. Li, X. Li, G. Li, W. Xiong, Z. Zeng, Neoantigen vaccine: An emerging tumor immunotherapy. *Mol. Cancer* **18**, 128–142 (2019).

11. T. Ouspenskaia, T. Law, K. R. Clauser, S. Klaeger, S. Sarkizova, F. Aguet, B. Li, E. Christian, B. A. Knisbacher, P. M. le, C. R. Hartigan, H. Keshishian, A. Apffel, G. Oliveira, W. Zhang, S. Chen, Y. T.

Chow, Z. Ji, I. Jungreis, S. A. Shukla, S. Justesen, P. Bachireddy, M. Kellis, G. Getz, N. Hacohen, D. B. Keskin, S. A. Carr, C. J. Wu, A. Regev, Unannotated proteins expand the MHC-I-restricted immunopeptidome in cancer. *Nat. Biotechnol.* **40**, 209–217 (2022).

12. C. C. Smith, S. R. Selitsky, S. Chai, P. M. Armistead, B. G. Vincent, J. S. Serody, Alternative tumour-specific antigens. *Nat. Rev. Cancer* **19**, 465–478 (2019).

13. A. Dondi, U. Lischetti, F. Jacob, F. Singer, N. Borgsmüller, R. Coelho, Tumor Profiler Consortium, R. Aebersold, M. Ak, F. S. al-Quaddoomi, S. I. Albert, J. Albinus, I. Alborelli, S. Andani, P.O. Attinger, M. Bacac, D. Baumhoer, B. Beck-Schimmer, C. Beisel, L. Bernasconi, A. Bertolini, B. Bodenmiller, X. Bonilla, L. Bosshard, B. Calgua, R. Casanova, S. Chevrier, N. Chicherova, M. D'Costa, E. Danenberg, N. Davidson, M.A. Drăgan, R. Dummer, S. Engler, M. Erkens, K. Eschbach, C. Esposito, A. Fedier, P. Ferreira, J. Ficek, A. L. Frei, B. Frey, S. Goetze, L. Grob, G. Gut, D. Günther, M. Haberecker, P. Haeuptle, S. Herter, R. Holtackers, T. Huesser, A. Immer, A. Irmisch, A. Jacobs, T. M. Jaeger, K. Jahn, A. R. James, P. M. Jermann, A. Kahles, A. Kahraman, V. H. Koelzer, W. Kuebler, J. Kuipers, C. P. Kunze, C. Kurzeder, K.V. Lehmann, M. Levesque, U. Lischetti, S. Lugert, G. Maass, M. G. Manz, P. Markolin, M. Mehnert, J. Mena, J. M. Metzler, N. Miglino, E. S. Milani, H. Moch, S. Muenst, R. Murri, C. K. Y. Ng, S. Nicolet, M. Nowak, M. N. Lopez, P. G. A. Pedrioli, L. Pelkmans, S. Piscuoglio, M. Prummer, N. Rimmer, M. Ritter, C. Rommel, M. L. Rosano-González, G. Rätsch, N. Santacroce, J. S. del Castillo, R. Schlenker, P. C. Schwalie, S. Schwan, T. Schär, G. Senti, W. Shao, S. Sivapatham, B. Snijder, B. Sobottka, V. T. Sreedharan, S. Stark, D. J. Stekhoven, T. Tanna, A. P. A. Theocharides, T. M. Thomas, M. Tolnay, V. Tosevski, N. C. Toussaint, M. A. Tuncel, M. Tusup, A. van Drogen, M. Vetter, T. Vlajnic, S. Weber, W. P. Weber, R. Wegmann, M. Weller, F. Wendt, N. Wey, A. Wicki, M. H. E. Wildschut, B. Wollscheid, S. Yu, J. Ziegler, M. Zimmermann, M. Zoche, G. Zuend, V. Heinzelmann-Schwarz, C. Beisel, N. Beerenwinkel, Detection of isoforms and genomic alterations by high-throughput full-length single-cell RNA sequencing in ovarian cancer. *Nat. Commun.* **14**, 7780–7799 (2023).

14. G. Li, S. Mahajan, S. Ma, E. D. Jeffery, X. Zhang, A. Bhattacharjee, M. Venkatasubramanian, M. T. Weirauch, E. R. Miraldi, H. L. Grimes, G. M. Sheynkman, T. Tilburgs, N. Salomonis, Splicing neoantigen discovery with SNAF reveals shared targets for cancer immunotherapy. *Sci. Transl. Med.* **16**, eade2886 (2024).

15. T. Wang, Y. Cui, J. Jin, J. Guo, G. Wang, X. Yin, Q.Y. He, G. Zhang, Translating mRNAs strongly correlate to proteins in a multivariate manner and their translation ratios are phenotype specific. *Nucleic Acids Res.* **41**, 4743–4754 (2013).

16. S. Xu, X. Wang, C. Fei, A highly effective system for predicting MHC-II epitopes with immunogenicity. *Front. Oncol.* **12**, 888556 (2022).

17. Z. Lai, A. Markovets, M. Ahdesmaki, B. Chapman, O. Hofmann, R. McEwen, J. Johnson, B. Dougherty, J. C. Barrett, J. R. Dry, VarDict: A novel and versatile variant caller for next-generation sequencing in cancer research. *Nucleic Acids Res.* **44**, e108 (2016).

18. C. H. Lee, J. Huh, P. R. Buckley, M. Jang, M. P. Pinho, R. A. Fernandes, A. Antanaviciute, A. Simmons, H. Koohy, A robust deep learning workflow to predict $CD8^+$ T-cell epitopes. *Genome Med.* **15**, 70–94 (2023).

19. B. Chen, M.S. Khodadoust, N. Olsson, L.E. Wagar, E. Fast, C.L. Liu, Y. Muftuoglu, B.J. Sworder, M. Diehn, R. Levy, M.M. Davis, J.E. Elias, R.B. Altman, A.A. Alizadeh, Predicting HLA class II antigen presentation through integrated deep learning. *Nat. Biotechnol.* **37**, 1332–1343 (2019).

20. B. Reynisson, B. Alvarez, S. Paul, B. Peters, M. Nielsen, NetMHCpan-4.1 and NetMHCIIpan-4.0: Improved predictions of MHC antigen presentation by concurrent motif deconvolution and integration of MS MHC eluted ligand data. *Nucleic Acids Res.* **48**, W449–W454 (2020).

21. J. Cheng, K. Bendjama, K. Rittner, B. Malone, BERTMHC: Improved MHC-peptide class II interaction prediction with transformer and multiple instance learning. *Bioinformatics* **37**, 4172–4179 (2021).

22. T. V. Moore, M. I. Nishimura, Improved MHC II epitope prediction—A step towards personalized medicine. *Nat. Rev. Clin. Oncol.* **17**, 71–72 (2020).

23. A. Marcu, L. Bichmann, L. Kuchenbecker, D. J. Kowalewski, L. K. Freudenmann, L. Backert, L. Mühlenbruch, A. Szolek, M. Lübke, P. Wagner, T. Engler, S. Matovina, J. Wang, M. Hauri-Hohl, R. Martin, K. Kapolou, J. S. Walz, J. Velz, H. Moch, L. Regli, M. Silginer, M. Weller, M. W. Löffler, F. Erhard, A. Schlosser, O. Kohlbacher, S. Stevanović, H.G. Rammensee, M. C. Neidert, HLA Ligand

Atlas: A benign reference of HLA-presented peptides to improve T-cell-based cancer immunotherapy. *J. Immunother. Cancer* **9**, e002071 (2021).

24. S. E. Brightman, A. Becker, R. R. Thota, M. S. Naradikian, L. Chihab, K. S. Zavala, A. L. Ramamoorthy Premlal, R. Q. Griswold, J. S. Dolina, E. E. W. Cohen, A. M. Miller, B. Peters, S. P. Schoenberger, Neoantigen-specific stem cell memory-like $CD4^+$ T cells mediate $CD8^+$ T cell-dependent immunotherapy of MHC class II-negative solid tumors. *Nat. Immunol.* **24**, 1345–1357 (2023).

25. E. Alspach, D. M. Lussier, A. P. Miceli, I. Kizhvatov, M. DuPage, A. M. Luoma, W. Meng, C. F. Lichti, E. Esaulova, A. N. Vomund, D. Runci, J. P. Ward, M. M. Gubin, R. F. V. Medrano, C. D. Arthur, J. M. White, K. C. F. Sheehan, A. Chen, K. W. Wucherpfennig, T. Jacks, E. R. Unanue, M. N. Artyomov, R. D. Schreiber, MHC-II neoantigens shape tumour immunity and response to immunotherapy. *Nature* **574**, 696–701 (2019).

26. A. Butler, P. Hoffman, P. Smibert, E. Papalexi, R. Satija, Integrating single-cell transcriptomic data across different conditions, technologies, and species. *Nat. Biotechnol.* **36**, 411–420 (2018).

27. J. T. Hung, I.J. Chen, S.H. Ueng, C.S. Huang, S.C. Chen, M.Y. Chen, Y.C. Lin, C.Y. Lin, M. J. Campbell, H. S. Rugo, A. L. Yu, The clinical relevance of humoral immune responses to Globo H-KLH vaccine adagloxad simolenin (OBI-822)/OBI-821 and expression of Globo H in metastatic breast cancer. *J. Immunother. Cancer* **10**, e004312 (2022).

28. R. Bill, P. Wirapati, M. Messemaker, W. Roh, B. Zitti, F. Duval, M. Kiss, J. C. Park, T. M. Saal, J. Hoelzl, D. Tarussio, F. Benedetti, S. Tissot, L. Kandalaft, M. Varrone, G. Ciriello, T. A. McKee, Y. Monnier, M. Mermod, E. M. Blaum, I. Gushterova, A. L. K. Gonye, N. Hacohen, G. Getz, T. R. Mempel, A. M. Klein, R. Weissleder, W. C. Faquin, P. M. Sadow, D. Lin, S. I. Pai, M. Sade-Feldman, M. J. Pittet, *CXCL9:SPP1* macrophage polarity identifies a network of cellular programs that control human cancers. *Science* **381**, 515–524 (2023).

29. Z. Zhang, Z. Pan, Q. Li, Q. Huang, L. Shi, Y. Liu, Rational design of ICD-inducing nanoparticles for cancer immunotherapy. *Sci. Adv.* **10**, eadk0716 (2024).

30. J. Gungabeesoon, N. A. Gort-Freitas, M. Kiss, E. Bolli, M. Messemaker, M. Siwicki, M. Hicham, R. Bill, P. Koch, C. Cianciaruso, F. Duval, C. Pfirschke, M. Mazzola, S. Peters, K. Homicsko, C. Garris, R. Weissleder, A. M. Klein, M. J. Pittet, A neutrophil response linked to tumor control in immunotherapy. *Cell* **186**, 1448–1464.e20 (2023).

31. M. Casarrubios, M. Provencio, E. Nadal, A. Insa, M. del Rosario García-Campelo, M. Lázaro-Quintela, M. Dómine, M. Majem, D. Rodriguez-Abreu, A. Martinez-Marti, J. de Castro Carpeño, M. Cobo, G. López Vivanco, E. del Barco, R. Bernabé, N. Viñolas, I. Barneto Aranda, B. Massuti, B. Sierra-Rodero, C. Martinez-Toledo, I. Fernández-Miranda, R. Serna-Blanco, A. Romero, V. Calvo, A. Cruz-Bermúdez, Tumor microenvironment gene expression profiles associated to complete pathological response and disease progression in resectable NSCLC patients treated with neoadjuvant chemoimmunotherapy. *J. Immunother. Cancer* **10**, e005320 (2022).

32. M. J. Lin, J. Svensson-Arvelund, G. S. Lubitz, A. Marabelle, I. Melero, B. D. Brown, J. D. Brody, Cancer vaccines: The next immunotherapy frontier. *Nat. Cancer* **3**, 911–926 (2022).

33. E. Blass, P. A. Ott, Advances in the development of personalized neoantigen-based therapeutic cancer vaccines. *Nat. Rev. Clin. Oncol.* **18**, 215–229 (2021).

34. T. Fan, M. Zhang, J. Yang, Z. Zhu, W. Cao, C. Dong, Therapeutic cancer vaccines: Advancements, challenges, and prospects. *Signal Transduct. Target. Ther.* **8**, 450–473 (2023).

35. C. M. Laumont, K. Vincent, L. Hesnard, É. Audemard, É. Bonneil, J.P. Laverdure, P. Gendron, M. Courcelles, M.P. Hardy, C. Côté, C. Durette, C. St-Pierre, M. Benhammadi, J. Lanoix, S. Vobecky, E. Haddad, S. Lemieux, P. Thibault, C. Perreault, Noncoding regions are the main source of targetable tumor-specific antigens. *Sci. Transl. Med.* **10**, eaau5516 (2018).

36. D. Weber, J. ibn-Salem, P. Sorn, M. Suchan, C. Holtsträter, U. Lahrmann, I. Vogler, K. Schmoldt, F. Lang, B. Schrörs, M. Löwer, U. Sahin, Accurate detection of tumor-specific gene fusions reveals strongly immunogenic personal neo-antigens. *Nat. Biotechnol.* **40**, 1276–1284 (2022).

37. L. Liu, J. Chen, H. Zhang, J. Ye, C. Moore, C. Lu, Y. Fang, Y.X. Fu, B. Li, Concurrent delivery of immune checkpoint blockade modulates T cell dynamics to enhance neoantigen vaccine-generated antitumor immunity. *Nat. Cancer* **3**, 437–452 (2022).

38. J. Borst, T. Ahrends, N. Babala, C. J. M. Melief, W. Kastenmuller, $CD4^{+}$ T cell help in cancer immunology and immunotherapy. *Nat. Rev. Immunol.* **18**, 635–647 (2018).

39. S. Kreiter, M. Vormehr, N. van de Roemer, M. Diken, M. Löwer, J. Diekmann, S. Boegel, B. Schrörs, F. Vascotto, J. C. Castle, A. D. Tadmor, S. P. Schoenberger, C. Huber, Ö. Türeci, U. Sahin, Mutant MHC class II epitopes drive therapeutic immune responses to cancer. *Nature* **520**, 692–696 (2015).

40. Z. Li, X. Lai, S. Fu, L. Ren, H. Cai, H. Zhang, Z. Gu, X. Ma, K. Luo, Immunogenic cell death activates the tumor immune microenvironment to boost the immunotherapy efficiency. *Adv. Sci.* **9**, e2201734 (2022).

41. J. Chang, MHC multimer: A molecular toolbox for immunologists. *Mol. Cells* **44**, 328–334 (2021).

42. D. K. Wells, M. M. van Buuren, K. K. Dang, V. M. Hubbard-Lucey, K. C. F. Sheehan, K. M. Campbell, A. Lamb, J. P. Ward, J. Sidney, A. B. Blazquez, A. J. Rech, J. M. Zaretsky, B. Comin-Anduix, A. H. C. Ng, W. Chour, T. V. Yu, H. Rizvi, J. M. Chen, P. Manning, G. M. Steiner, X. C. Doan, Tumor Neoantigen Selection Alliance, T. Merghoub, J. Guinney, A. Kolom, C. Selinsky, A. Ribas, M. D. Hellmann, N. Hacohen, A. Sette, J. R. Heath, N. Bhardwaj, F. Ramsdell, R. D. Schreiber, T. N. Schumacher, P. Kvistborg, N. A. Defranoux, Key parameters of tumor epitope immunogenicity revealed through a consortium approach improve neoantigen prediction. *Cell* **183**, 818–834.e13 (2020).

43. A. B. Vogel, I. Kanevsky, Y. Che, K. A. Swanson, A. Muik, M. Vormehr, L. M. Kranz, K. C. Walzer, S. Hein, A. Güler, J. Loschko, M. S. Maddur, A. Ota-Setlik, K. Tompkins, J. Cole, B. G. Lui, T. Ziegenhals, A. Plaschke, D. Eisel, S. C. Dany, S. Fesser, S. Erbar, F. Bates, D. Schneider, B. Jesionek, B. Sänger, A.K. Wallisch, Y. Feuchter, H. Junginger, S. A. Krumm, A. P. Heinen, P. Adams-Quack, J. Schlereth, S. Schille, C. Kröner, R. de la Caridad Güimil Garcia, T. Hiller, L. Fischer, R. S. Sellers, S. Choudhary, O. Gonzalez, F. Vascotto, M. R. Gutman, J. A. Fontenot, S. Hall-Ursone, K. Brasky, M. C. Griffor, S. Han, A. A. H. Su, J. A. Lees, N. L. Nedoma, E. H. Mashalidis, P. V. Sahasrabudhe, C. Y. Tan, D. Pavliakova,

G. Singh, C. Fontes-Garfias, M. Pride, I. L. Scully, T. Ciolino, J. Obregon, M. Gazi, R. Carrion Jr, K. J. Alfson, W. V. Kalina, D. Kaushal, P.Y. Shi, T. Klamp, C. Rosenbaum, A. N. Kuhn, Ö. Türeci, P. R. Dormitzer, K. U. Jansen, U. Sahin, BNT162b vaccines protect rhesus macaques from SARS-CoV-2. *Nature* **592**, 283–289 (2021).

44. L. Zheng, S. Qin, W. Si, A. Wang, B. Xing, R. Gao, X. Ren, L. Wang, X. Wu, J. Zhang, N. Wu, N. Zhang, H. Zheng, H. Ouyang, K. Chen, Z. Bu, X. Hu, J. Ji, Z. Zhang, Pan-cancer single-cell landscape of tumor-infiltrating T cells. *Science* **374**, abe6474 (2021).

45. L. Zhang, X. Yu, L. Zheng, Y. Zhang, Y. Li, Q. Fang, R. Gao, B. Kang, Q. Zhang, J. Y. Huang, H. Konno, X. Guo, Y. Ye, S. Gao, S. Wang, X. Hu, X. Ren, Z. Shen, W. Ouyang, Z. Zhang, Lineage tracking reveals dynamic relationships of T cells in colorectal cancer. *Nature* **564**, 268–272 (2018).

46. G. P. Linette, M. Becker-Hapak, Z. L. Skidmore, M. L. Baroja, C. Xu, J. Hundal, D. H. Spencer, W. Fu, C. Cummins, M. Robnett, S. Kaabinejadian, W. H. Hildebrand, V. Magrini, R. Demeter, A. S. Krupnick, O. L. Griffith, M. Griffith, E. R. Mardis, B. M. Carreno, Immunological ignorance is an enabling feature of the oligo-clonal T cell response to melanoma neoantigens. *Proc. Natl. Acad. Sci. U.S.A.* **116**, 23662–23670 (2019).

47. X. Qiu, Q. Mao, Y. Tang, L. Wang, R. Chawla, H. A. Pliner, C. Trapnell, Reversed graph embedding resolves complex single-cell trajectories. *Nat. Methods* **14**, 979–982 (2017).

48. A. Liberzon, C. Birger, H. Thorvaldsdóttir, M. Ghandi, J. P. Mesirov, P. Tamayo, The Molecular Signatures Database (MSigDB) hallmark gene set collection. *Cell Syst.* **1**, 417–425 (2015).

49. S. Tang, A. Lomsadze, M. Borodovsky, Identification of protein coding regions in RNA transcripts. *Nucleic Acids Res.* **43**, e78 (2015).

50. I. T. Rombel, K. F. Sykes, S. Rayner, S. A. Johnston, ORF-FINDER: A vector for high-throughput gene identification. *Gene* **282**, 33–41 (2002).

51. M. Tardaguila, L. de la Fuente, C. Marti, C. Pereira, F. J. Pardo-Palacios, H. del Risco, M. Ferrell, M. Mellado, M. Macchietto, K. Verheggen, M. Edelmann, I. Ezkurdia, J. Vazquez, M. Tress, A. Mortazavi,

L. Martens, S. Rodriguez-Navarro, V. Moreno-Manzano, A. Conesa, SQANTI: Extensive characterization of long-read transcript sequences for quality control in full-length transcriptome identification and quantification. *Genome Res.* **28**, 396–411 (2018).
